# Dual Photo- and pH-Responsive Foldamer Metallogels

**DOI:** 10.1021/jacs.6c07355

**Published:** 2026-07-30

**Authors:** Moosa Wasim, Benjamin M. Gallant, Neha Yadav, Craig Fraser, Sena Öztürk, Louise Male, Dominik J. Kubicki, Maria C. Arno, Sarah J. Pike

**Affiliations:** † School of Chemistry, 1724 University of Birmingham, Edgbaston, Birmingham B15 2TT, U.K.; ‡ Department of Cancer and Genomic Sciences, University of Birmingham, Edgbaston, Birmingham B15 2TT, U.K.

## Abstract

Foldamers have been widely used as responsive motifs within discrete supramolecular systems on account of their programmable stimuli-sensitivity. However, translation of this functionality into bulk matter and toward the creation of smart materials, such as switchable metallogels, remains largely unexplored. Further, characterization of local structure in metallogel networks is inherently challenging because of their semisolid nature, precluding the use of solution phase NMR spectroscopy. We report a dual light- and pH-responsive foldamer metallogel, comprised of aromatic oligoamide foldamers coordinated to palladium­(II) centers. We show reversible gel–sol–gel behavior using UV illumination and subsequent heat treatment. Exposure to aqueous acid also induces a gel–sol transition. We determine the local structure of the gel using magic angle spinning (MAS) dynamic nuclear polarization (DNP) NMR spectroscopy. We thus identify square planar 4-fold coordination of Pd (II) by terminal pyridines in the foldamer as the primary structural motif comprising the gel backbone. Finally, successful exchange of the organic solvent with water, without compromising the gel structure, leads to the first example of a metallo-foldamer hydrogel.

## Graphical abstract

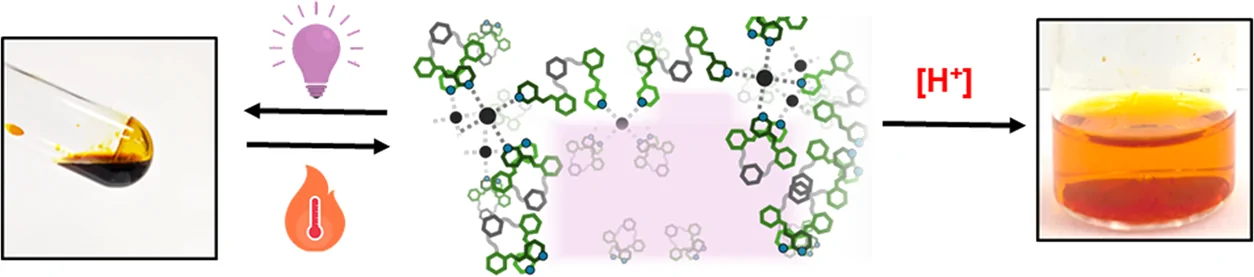

## Introduction

Foldamers are typically defined as oligomers with a strong tendency to adopt a specific, compact conformation, often resulting from intramolecular interactions such as hydrogen bonding or π-stacking.^1^ Traditionally, foldamer research has primarily sought to investigate functionality arising from molecular-level interactions with the foldamer, including host–guest binding, catalysis or antimicrobial activity.^2–7^ The conformational flexibility of foldamers has also led to their use as stimuli-sensitive components in supramolecular architectures. For example, there are several reports of light-responsive foldamers with proposed applications including chiral guest release and anion regulation.^8–11^ Meanwhile, pH-responsive and redox-responsive foldamers have been used in dynamic supramolecular systems to convert between alternate conformations and topologies.^12^

The application of foldamers within materials science has also been explored. Previous research showcases the self-assembly of foldamers into arrangements mimicking secondary protein structure as well as fibrous metal–organic networks.^13–15^ In crystal engineering, foldamers have been shown to selectively influence the morphologies of growing crystals or mimic the design of virus nanoparticles.^16,17^ Li and co-workers reported that hydrazide foldamers form organogels that demonstrate glucose-mediated dynamic helicity induction and amplification.^18^ Amphiphilic shape-persistent cyclo^6^-aramides and diethylammonium chloride have been shown to generate a two-component gel that can discriminate native arginine from 19 other amino acids.^19^ However, to date, the responsive behavior of foldamer scaffolds has remained largely underexplored. In polymer chemistry, mechano-responsive materials have been developed that integrate flexible foldamer motifs into polymer networks.^20^ Shu et al. have reported phosphorescent Pt­(II) foldamer complexes as anticounterfeit inks,^21^ while Kwon et al. demonstrated magnetotactic foldamer assemblies as a platform for the creation of micromachines.^22^ More recently, Ikeda has reported a reversibly expandable foldamer gel responsive to changes in redox potential.^23^ To the best of our knowledge, Ikeda’s work remains the only example of a stimulus-sensitive foldamer gel.

Gels are a frequent choice in the design of responsive materials. The ability to alternate between semisolid and liquid states on application of an external stimulus enables applications in environmental sensors or guest carriers.^24,25^ An intriguing and quickly growing class of gels are metallogels which, unlike covalently cross-linked gels, are constructed via weaker metal–ligand coordination, which can provide increased sensitivity to external stimuli.^26^ We hypothesized that the reversible nature of coordination within metallogel networks might enable disassembly through different stimuli, enabling orthogonal control over gel–sol transitions. Coupling this structural reversibility with stimuli-sensitive foldamer motifs make foldamer metallogels an attractive option for the design of multi stimuli-responsive materials. However, despite their enhanced functionality, no foldamer metallogels exhibiting responsive behavior have been proposed to date, limiting their application as smart materials.^27,28^ Although macroscopic gel behavior is widely explored through testing of the critical gelation concentration, gelation time, thermal response, in addition to rheology, microscopy and fourier transform infrared spectroscopy (FTIR) and UV–vis spectroscopy detailed atomic- and molecular-level characterization of metallogels, notably the nature of linker-metal coordination at the metal center, is notoriously challenging due to the high degree of disorder and dilution of these species in the gel.^29^ The ability to relate local structure to responsivity will enable rational design of responsive metallogels, which is one of the key challenges in the field today.^27–32^ Finally, metallofoldamer gels that are compatible with aqueous media would enable encapsulation of water-soluble cargos, but no hydrogels of this type have been reported as of yet (Figure 1).^33,34^

**Figure 1:**
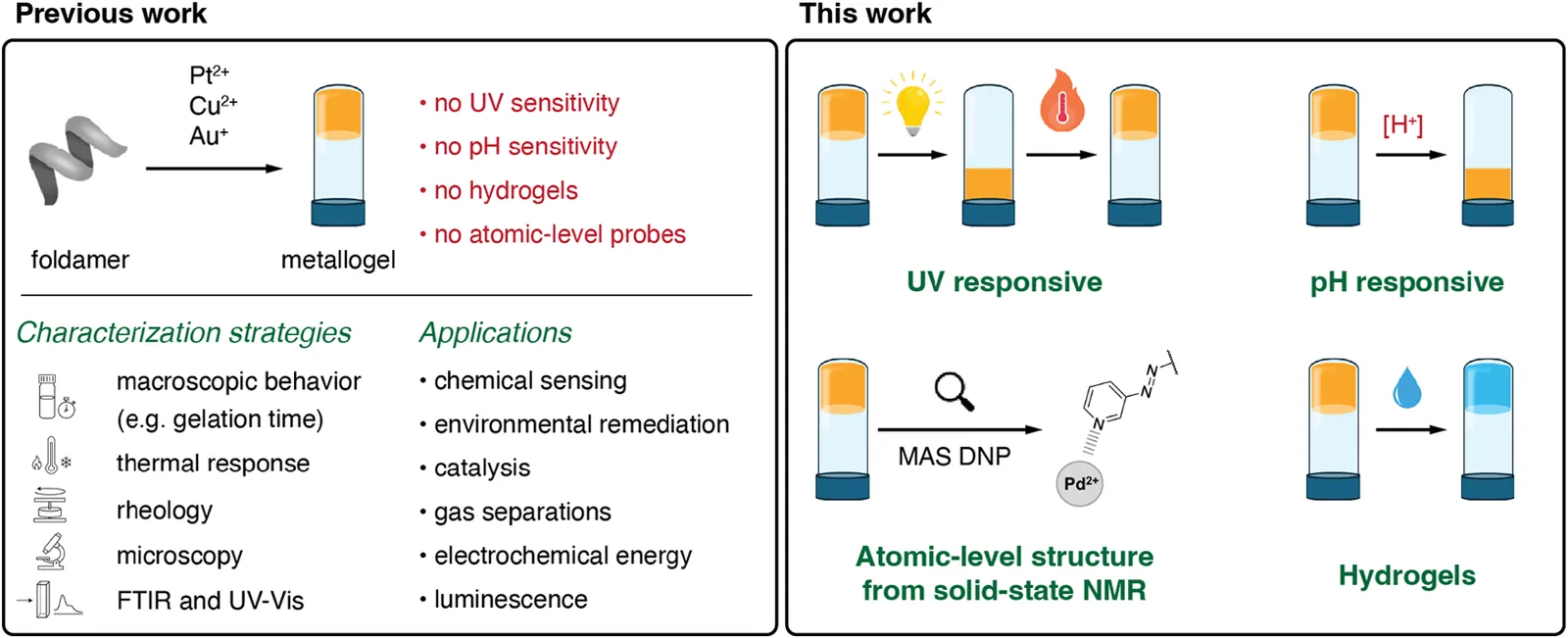
Overview of previous work on foldamer metallogels and the distinct aspects of this work.^25,26^

Here, we report the first example of a multistimuli-responsive foldamer gel, comprising an oligoazobenzene dicarboxamidefoldamer and palladium­(II), which demonstrates sol–gel responsivity to light and pH. Ultraviolet (UV) light is used to induce isomerization of the foldamer azobenzene groups, driving a gel–sol transition that can then be reversed using heat. Orthogonally, the gel can also be disassembled using a pH trigger to recover the foldamer. Using ^15^N MAS DNP NMR spectroscopy, we first investigate the local structure of the metallofoldamer gel backbone, including determining the coordination geometry of the metal center. Then, we establish the structural changes resulting in response to external stimuli. Finally, we demonstrate that the organogel can be converted completely into a hydrogel via solvent exchange with water.

## Results and Discussion

To enable the self-assembly of a dual-stimuli-responsive metallofoldamer gel, we first consider the design criteria of the two building blocks: the foldamer ligand and the metal center. First, the foldamer must adopt a stable helical conformation, function as a good ligand for a suitable metal ion and exhibit sensitivity to light and pH. Accordingly, we developed ligand **1**, which is based on the stimuli-responsive oligoazobenzene dicarboxamide foldamers previously reported by our group.^12^ **1** adopts a stable helical conformation due to the presence of (a) the bifurcated, intramolecular hydrogen-bonding interaction expected to occur between amide protons and the nitrogen atom of the adjacent pyridine unit, leading to a *syn-syn* conformation, and (b) the presence of *E*-configuration about the azobenzene units (Figure 2a). The incorporation of terminal pyridines permits **1** to act as a ligand for square-planar metal centers (e.g., Pd­(II)), circumventing challenges of foldamer steric bulk hindering complexation. Simultaneously, since the terminal pyridines can be protonated, they enable orthogonal control over metal coordination using pH.^35^ Finally, incorporation of photoresponsive azobenzene units into the foldamer backbone enables photoinduced isomerization of the azo bonds. We use [Pd­(CH_3_CN)_4_]­(BF_4_)_2_ to introduce Pd^2+^ into the gel, as the square-planar geometry at the metal center generates four binding sites for pyridine when combined with the weakly coordinating [BF_4_]^−^ counterions. Pd-pyridine binding promotes the coordination of multiple foldamer units to the metal center to enable self-assembly of the metallogel network.^36,37^ Pd­(II)-based metallogels were selected to be the focus of this study due several design consideration factors, including the well-established ability of Pd­(II) to form directional coordinate Pd­(II)-N bonds with pyridine which has been widely used in supramolecular chemistry^36–39^ to assemble structures as well as for achieving controlled molecular switching (i.e., stimuli-responsive behavior) in supramolecular systems.^36,40,36,40^ Additionally, the predictable, square-planar coordination geometry adopted by the Pd­(II) ion permits up to four pyridine-capped foldamers to complex to the same metal center, thus, enabling the formation of the desired coordination networks and facilitate gelation. Finally, given the recent reports of Pd­(II) metallogels finding prospective uses in catalysis^41,42^ and in biomedical applications,^43^ which could be of longer-term interest to Pd­(II)-based foldamer metallogels, it was decided that Pd­(II) would be used as the metal source for the metallogels generated in this study.

**Figure 2:**
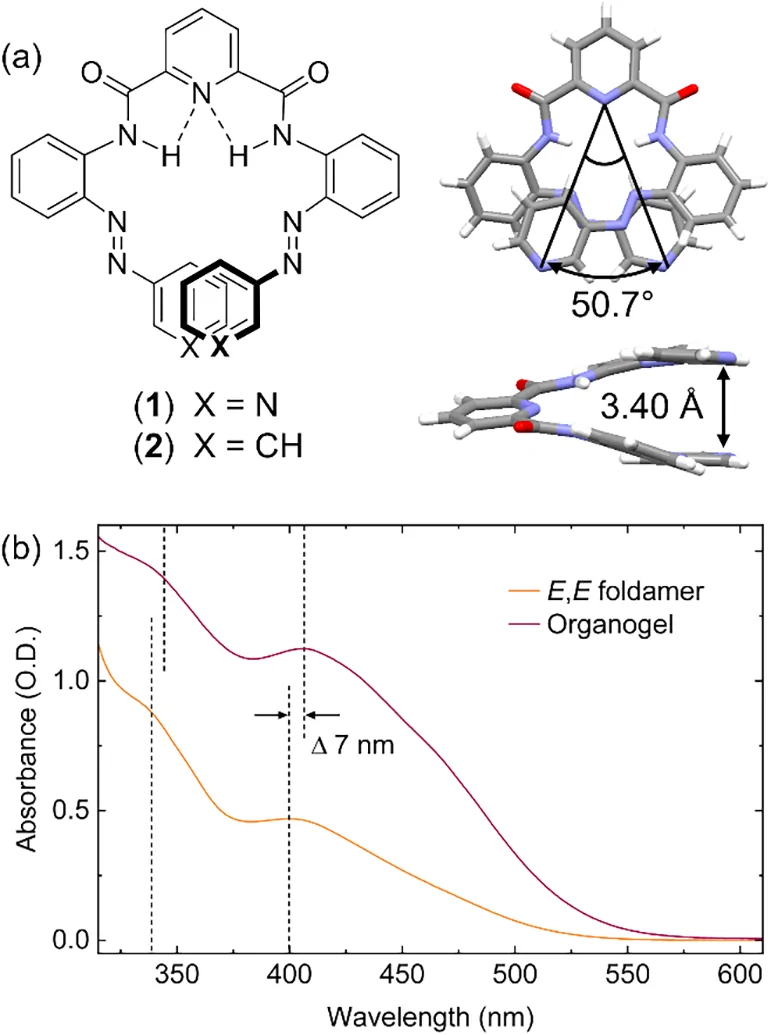
(a) Structure of foldamers **1** and **2** used in this study. (b) Crystal structure of foldamer **1** showing the helical conformation in the solid state, derived from single crystal X-ray diffraction. The bite angle between pyridyl nitrogen atoms (50.7°) and helical pitch (∼3.40 Å) are shown. (c) UV–visible absorbance spectra of foldamer **1** in 3:7 acetonitrile:TCE (6 × 10^–5^ M) and thin layer (<100 μm) of pristine metallogel at 25 °C.

The pyridine-terminated foldamer **1** was synthesized in a five step procedure from commercially available starting materials in accordance with known or modified literature procedures (Scheme S1, Figures S1,S2).^8,12,44–47^ **1** has been fully characterized by solution phase ^1^H and ^13^C NMR spectroscopy (Supporting Information), including 2D ^1^H–^1^H nuclear Overhauser effect spectroscopy (NOESY) and ^1^H–^1^H correlation spectroscopy (COSY) (Figures S3,S4), mass spectrometry, IR spectroscopy, melting point analysis (Supporting Information), single crystal X-ray diffraction analysis (Figures 2a, S5–S7), and UV–visible spectroscopy (Figures 2b, S8,S9). Notably, the adoption of a stable helical conformation by **1** in solution was confirmed through the observation of cross peaks between the NH amide proton (H_*c*_) and the pyridyl proton (H_*h*_) by NOESY. The presence of a helical conformation was identified in the solid state via single crystal X-ray diffraction analysis of **1** crystals grown by slow evaporation of solvent from a solution of **1** in tetrachloroethane (Figure 2a). The *syn-syn* conformation adopted about the central pyridylcarboxamide unit and *E*-geometry of the azobenzene units lead to a stable helical conformation of this foldamer in the solid state.

UV–visible absorbance spectroscopy was used to investigate the photo- and thermoresponsive *E-Z* isomerization of the azo bonds in the foldamer. A 6 × 10^–5^ M solution of **1** in acetonitrile displayed a strong band at 383 nm, corresponding to the π → π* transition of the *E*,*E*-isomer of **1**.^8^ 30 s of ex-situ illumination of this solution with 395 nm light led to a photostationary state (PSS) equilibrium (Figure S8). Subsequently, thermal *Z*-to-*E* relaxation of the azo bonds in the foldamer was observed to occur over 10 h at 40 °C, as shown by the UV–visible spectrum returning to that of the original *E*,*E*-isomer of **1**. Thus, the photo- and thermo-responsivity of **1** have been established. (Figure S9).

To explore the self-assembly of the metallogel from the photoresponsive foldamer ligand and transition metals, 1 eq. of **1** was added to acetonitrile followed by addition of 0.5 eq. of palladium tetrakisacetonitrile tetrafluoroborate. After 16 h at ambient temperature in the dark, the mixture was found to have produced an opaque, orange gel with a small amount of precipitate suspended within the bulk gel (Figure S12). We attribute the precipitation to the foldamer’s poor solubility in acetonitrile. Hence, to achieve a

homogeneous gel, we optimized the formulation to acetonitrile/tetrachloroethane (TCE) 3:7 (v/v) (Figure S13). The critical gel concentration was found to be 2 wt % (w/w), in the expected range for either a low molecular weight organic gelator (LMOG) or coordination polymer gel (Table S1, Figures S14–S16).^29^ While **1** has molecular weight >3000 Da, we note that classification as a LMOG is better suited for molecules which form gels primarily through self-assembly rather than metal ion coordination..^48^ Further studies into the effect of other solvents, foldamer:metal stoichiometry, temperature and metal counterions were undertaken, providing insight into the influence of these variables on gel formation (Tables S1–S3, Figures S17–S28). To establish the importance of metal–ligand complexation on enabling gel formation, we conducted a control experiment where **1** was replaced by a phenyl-terminated foldamer analogue under the optimized gelation conditions (Scheme S2). Full characterization of the phenyl-terminated foldamer was undertaken as for **1**, and its ability to adopt a stable helical conformation in solution and the solid state was confirmed (Supporting Information, Figures S29–S34). No gel formation was observed in this case, which we attribute to the inability of the phenyl-capped foldamer to bind the metal center. This result implies that Pd-pyridine coordination is a prerequisite for self-assembly of the gel network (Figure S35). It should also be noted that the varying intrinsic polarity of the pyridine- and phenyl-capped foldamers, **1** and **2** respectively, could also influence the ability of the system to gelate. **1** is composed of achiral monomers and, consequently, demonstrates no helical bias for the preferential adoption of either left- or right-handed screw-sense preference.^49,50^ **1** possesses an equal numbers of foldamer molecules displaying *M*- or *P*-helicity, as evidenced in solution using chiral dichroism (CD) spectroscopy and in the solid-state using X-ray crystallography (Supporting Information, Figures S5–S7 and S10).^51,52^ Furthermore, CD spectroscopy was also used to assess the behavior of the metallofoldamer. Hence, a 3.3 × 10^–5^ M^–1^ 3:7 acetonitrile/tetrachloroethane solution of **1** and palladium tetrakisacetonitrile tetrafluoroborate was monitored for 36 h in the dark at 295 K using CD spectroscopy. Over this time, no bands appeared in the CD spectrum between 350 and 550 nm, which indicates that under these tested conditions, that no chirality is induced into the system (Supporting Information, Figure S11).^12^ Given the achiral nature of **1**, the observed lack of induced helicity in the system by CD spectroscopy is as expected.

Having successfully formed the metallogel with **1**, we characterized its structure and properties using UV–visible, IR and NMR spectroscopies, and mass spectrometry. Figure 2b shows the UV–visible absorbance spectra of the neat foldamer and the metallogel. A small red shift from 400 nm in the neat foldamer to 407 nm in the neat metallogel is evident for the *E* azobenzene absorption. Red shifting of π-π* transitions in azobenzenes with the introduction of electron-withdrawing groups is well-known.^53^ Since palladium­(II) is an electron-deficient species, this result gave us a further indication that complexation of the foldamer with Pd^2+^ takes place in the gel. To further establish palladium-foldamer coordination, electrospray ionization mass spectrometry on a dissolved gel sample was conducted. Pd­(foldamer) fragments were observed (Figure S36), consistent with the formation of strong coordinate bonding between Pd^2+^ and the foldamer. IR spectroscopy of **1** showed several characteristic vibrations originating from aromatic C–H stretches at around 2996 cm^–1^. Contrasting this with the infrared spectrum of the metallogel, there is a distinct shift in the aromatic C–H signals which are now centered around 3054 cm^–1^ (Figure S37), which may be attributed to disruption of foldamer π-π stacking interactions upon gelation.

While these results are informative about the gel structure, they do not unequivocally identify either the site of Pd^2+^ coordination or geometry of the foldamer around the palladium center. Accordingly, we attempted to use solution phase ^1^H NMR spectroscopy, but these solution-based NMR experiments proved challenging to interpret due to the extreme broadening of signals (Figure S38). This broadening effect arises from the mixed solid–liquid nature of the gel at room temperature, which slows down molecular reorientation leading to a substantial shortening of the transverse relaxation time, *T*_2_.^54^

To overcome this challenge, we used DNP-enhanced ^13^C and ^15^N solid-state MAS NMR. Owing to the high dilution of the gel, we anticipated that DNP would be essential to record high-quality data.^55^ MAS NMR spectroscopy has proven powerful in establishing the atomic-level structure of amorphous materials, particularly when enhanced by DNP.^56–60^ However, to date, analysis of local structure via MAS NMR spectroscopy in intact metallogels has not been reported.^61,62^ Figure 3a shows DNP-enhanced ^1^H–^15^N CP MAS NMR spectra of the foldamer and organogel, each impregnated with a 16 mM solution of TEKPol in TCE, with a DNP enhancement factor (ε) of 72 for the organogel. This DNP enhancement results in a *ca*. 5200-fold reduction in experimental time, enabling data acquisition in just 12 h with a signal-to-noise ratio that would otherwise require *ca*. 7 years without DNP. (Table S4, Figures S40–S46). The line shape of the ^13^C signal of TCE in the gel confirms that the gel forms a glass when flash-frozen at 100 K, and we found that the material produces the same spectra when frozen and thawed multiple times. We have also attempted a room-temperature MAS NMR experiment, which confirmed that the amount of ^13^C signal after 14 h of acquisition without the benefit of DNP is insufficient for structural characterization (Figure S39).

**Figure 3:**
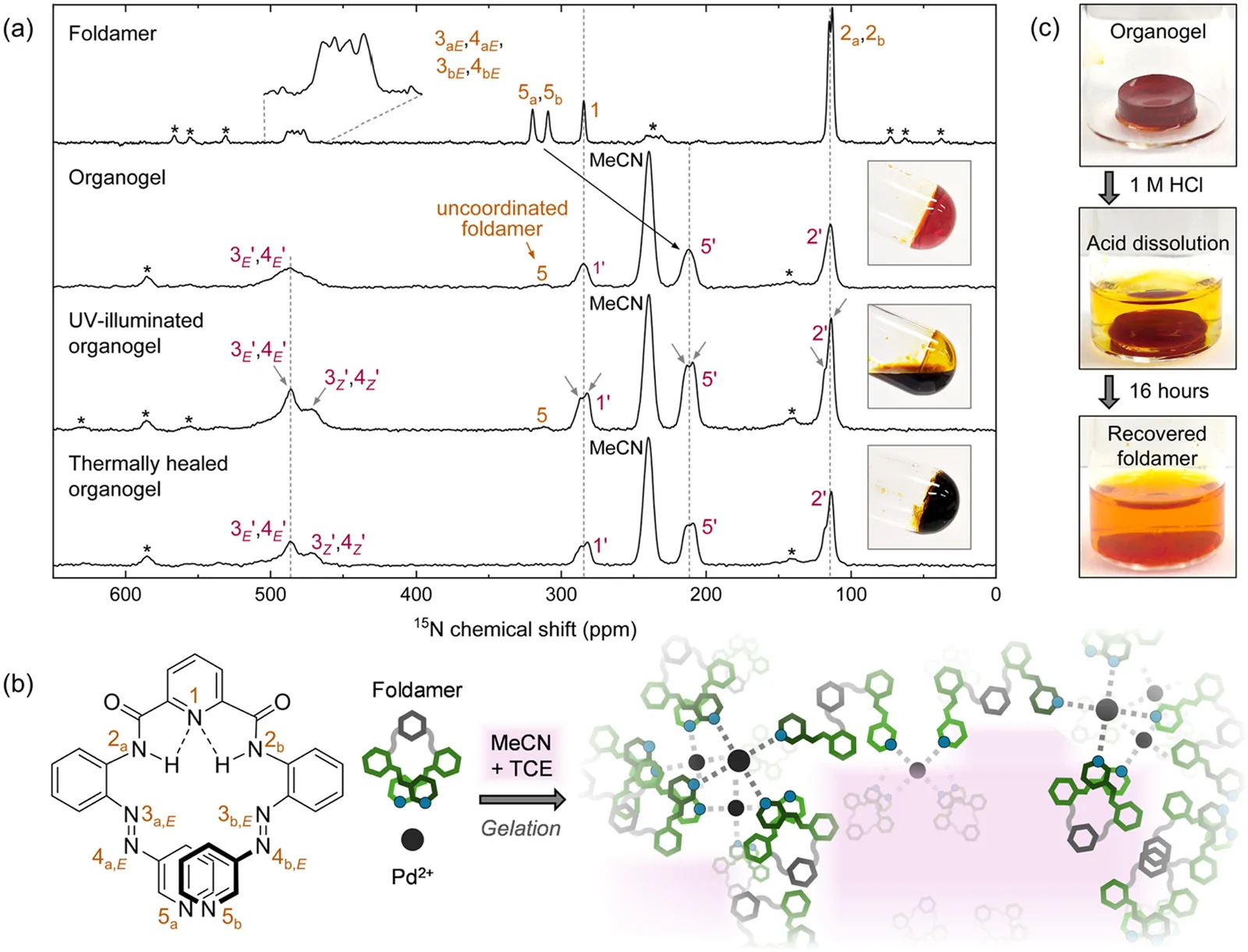
(a) ^1^H–^15^N CP MAS DNP NMR spectroscopy of the foldamer, metallogel, UV-illuminated metallogel and the thermally healed metallnogel at 9.4 T, 100 K, and 10 kHz MAS (foldamer) and 14 kHz MAS (all metallogel materials). Asterisks denote spinning sidebands. Insets show photographs of the metallogel at each stage of formation, dissolution and reformation. (b) Schematic diagram of a proposed structure of the gel network. (c) An metallogel disk: pristine (top), immediately after immersion in aqueous 1 M HCl (middle), after 16 h in aqueous 1 M HCl (bottom). Note the formation of an orange precipitate.

Substantial broadening of all ^15^N signals was observed in the organogel relative to the pristine foldamer, consistent with increased static disorder in the local environment of the foldamer and confirming incorporation of the foldamer into the gel structure. Moreover, a marked decrease in ^15^N chemical shift is observed for the terminal pyridine signals of the foldamer (labeled 5′, Figure 3a) In the neat foldamer, the two terminal pyridines are crystallographically distinct, producing two ^15^N signals at 319.8 and 309.2 ppm (5_a_ and 5_b_ although we do not assign these two environments to their specific signal). By contrast, in the gel these signals are not only strongly shifted to lower frequencies but merged to a single broad resonance at 212.0 ppm (5′), with other signals largely unchanged. These findings are consistent with coordination of the terminal pyridine groups to palladium­(II) in the gel backbone, with accompanying disorder in the local environment of the foldamer leading to signal broadening and overlap.^63,64^ Given the 2:1 foldamer:palladium­(II) stoichiometry used during metallogel synthesis, the absence of any substantial ^15^N signal corresponding to uncoordinated terminal pyridine groups (labeled 5) indicates an average 4-fold coordination of Pd^2+^ by the foldamer pyridines. Moreover, observation of ^15^N resonances from Pd^2+^-coordinated pyridine supports a square-planar geometry at the metal center, since Pd­(II) is generally paramagnetic in other coordination geometries. Paramagnetism of Pd­(II) would lead to substantial paramagnetic relaxation and hyperfine shifts on the directly coordinated ^15^N. Since these effects are not observed, we conclude that the metal center is diamagnetic square-planar Pd (II). The absence of any ^15^N signal in the organogel corresponding to acetonitrile coordinated to Pd­(II), which would lead to a relative shift of Δδ = −70 ppm, further confirms 4-coordinate square-planar geometry.^65^ Together, these results indicate that the gel backbone is composed of square-planar palladium­(II) centers, each coordinated by four different foldamer molecules. Notably, the possibility of bidentate coordination of one Pd^2+^ by a single foldamer molecule is excluded as the bite angle of the two terminal pyridines is too small for a square planar arrangement in this conformation (Figure 2a). The absence of ^15^N signal splitting upon foldamer incorporation into the gel suggests that both terminal pyridine groups of each foldamer molecule are coordinated to metal centers, generating a gel backbone likely composed of an extended cross-linked network structure, as illustrated schematically in Figure 3b. This allows us to identify the gel as coordination polymer, rather than a LMOG gel. The conformational flexibility required to achieve this is reflected in our previous work where the helical pitch of analogous foldamer scaffolds is shown to vary between 3.4 and 7.3 Å in relation to end group size.^66^ The rotatable bonds within the foldamer further facilitate foldamer-Pd­(II) cross-linking by further increasing the number of available conformations.

To investigate if the photoresponsive behavior of the neat foldamer is retained in the metallogel, we illuminated a thin sample with 395 nm light for 16 h. A complete gel–sol transition was observed, alongside a color change from red-orange to brown, consistent with *Z-*azobenzene formation (Figure 3a).^8 1^H–^15^N CP MAS DNP NMR spectroscopy of the pristine metallgel shows signals at 475–490 ppm, corresponding to the four inequivalent azobenzene environments expected of the foldamer in its *E-E* isomer (3_*E*_ and 4_*E*_; Figure 3b). Upon gelation, these signals broaden but remain centered at 485 ppm. After UV illumination, this azobenzene signature evolves, with two broad overlapping resonances observed and centered at 485 and 473 ppm. We attribute the lower frequency signal to foldamer containing *Z-*azobenzene units (3_*Z*_ and 4_*Z*_), indicating partial isomerization of foldamer molecules inside the gel network. The correlation between *Z-*isomer formation and the observed sol–gel transition suggests that isomerization drives network breakdown. Notably, no appreciable increase in uncoordinated foldamer is detected, implying both that minimal network cleavage is achieved by even substantial *E-Z* isomerization but that minimal cleavage is still sufficient to induce the sol state. Heating the sol at 60 °C for 2 h led to complete reformation of the gel, although the brown coloration remained, suggesting incomplete *Z-E* thermal relaxation of the azobenzene. ^15^N DNP NMR spectroscopy confirms this observation, showing that the reformed metallogel has undergone minimal reverse isomerism, with the two signals at 485 and 473 ppm remaining unchanged. Photoreversible gels based on azobenzene gelators usually display reversal of the *E*-azobenzene gel reformation, in contrast to our observations for this metallogel.^67–70^ Attempts were made to quantify the *E-Z* isomerism within the gel using liquid-state ^1^H NMR and UV–visible spectroscopy (Figures S49,S50). However, insufficient resolution in these spectra, likely a result of inhomogeneous broadening arising from slow molecular dynamics, prevented analysis of individual isomer signals.

Having established the local structure of the gel network and its response to environmental (light) stimuli, we next investigated the metallogel’s response to pH to establish if it can function as a multistimuli-responsive gel with orthogonal control over its physical properties. As the gel network is made up of interacting Pd-pyridine moieties, we hypothesized that the introduction of acid and a competitive ligand for palladium­(II) would result in rapid network breakdown due to protonation of the foldamer. To explore this, we immersed the metallogel in 1 M aqueous HCl at room temperature in the dark. After 16 h the gel was no longer discernible and crumbled to form an orange powder (Figure 3c). Upon filtration and washing with 1 M NaHCO_3_, this precipitate was found to be the original foldamer. Following attempted purification, the foldamer was recovered in a crude yield of 42% (Figure S51), confirming substantial deconstruction of the gel network.

To further investigate the versatility of the foldamer metallogel, we next explored solvent exchange to form a hydrogel analogue. Hydrogels are widely used for the encapsulation and delivery of water-soluble guest species.^71^ Gel backbone structures that are suitable for use in both organogels and hydrogels are thus highly desirable. Due to the insolubility of the foldamer in water, a gel could not be formed directly from foldamer **1** and [Pd­(CH_3_CN)_4_]­(BF_4_)_2_ in water. Furthermore, the immiscibility of water with the apolar organic solvents capable of solubilizing the foldamer, such as tetrachloroethane, prevented the use of a hybrid solvent system for gel formation. Therefore, following the formation of the organogel, we used a solvent exchange procedure to access a hydrogel.^72^ The organogel was first immersed in methanol for 3 days, followed by another 3 days of immersion in water, with the solvent bath replaced each day. ^1^H–^15^N CP MAS DNP NMR spectroscopy revealed that the local structure inside the gel backbone is unchanged by this solvent exchange process, with a minimal quantity of uncoordinated foldamer observed in both gels (Figure 4a). To better understand the microstructure of the hydrogel, we imaged it using cryo-scanning electron microscopy. As shown in Figure 4b, a dense, porous network structure with pore sizes on the μm scale was observed is seen with an average fiber thickness of 0.49 μm (Figures S52,S53).^73^

**Figure 4:**
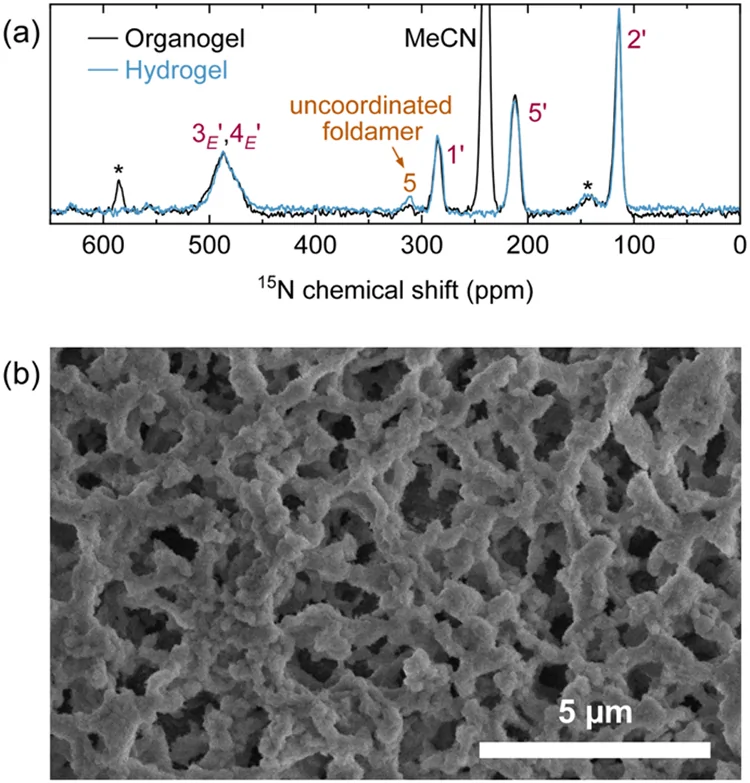
(a) ^1^H–^15^N CP MAS DNP NMR spectra of the organogel and the hydrogel formed via sequential solvent exchange. Signal assignments correspond to the structure shown in Figure 3b. (b) Cryo-scanning electron microscopy image of the hydrogel.

Oscillatory rheology was used as supporting characterization to assess the mechanical properties of the hydrogels (Figure S54). Amplitude-sweep measurements showed a clear linear viscoelastic region for the hydrogel formulations, with the storage modulus (*G′*) exceeding the loss modulus (*G″*), confirming predominantly elastic, gel-like behavior. Increasing foldamer loading from 3 to 5 wt % led to higher plateau G′ values, consistent with increased hydrogel stiffness at higher solid content. The onset of strain-induced network breakdown occurred at several percent strain, while the 5 wt % hydrogel did not show a *G′*/*G″* crossover within the investigated strain range. The large *G′* values, approaching 10^5^ Pa, indicate unusually stiff gels compared with many supramolecular gel systems. This high stiffness is consistent with the formation of an extended coordination-polymer-like network, in which metal–ligand connectivity and the relatively high foldamer loading contribute to mechanical reinforcement. Additionally, the relatively high G’ values approaching 100,000 Pa further demonstrate the dative nature of the coordination polymer network.

## Conclusion

We have established the first example of a stimuli-responsive reformable foldamer metallogel, which can be converted to a hydrogel. Using DNP NMR spectroscopy as an atomic-level probe, we establish the local structure of the gel, determining the nature and geometry of coordination around the Pd­(II) centers, and confirm that the gel owes its structural rigidity to a cross-linked metal–organic network based on this coordination motif. A reversible gel–sol–gel transition can be induced in this metallogel using sequential exposure to UV light followed by thermal healing, demonstrating the potential suitability of such gels for future use in the targeted uptake/release of small molecules. Further, we have determined the pH responsivity of the gel, achieving complete gel–sol transition with facile exposure to acidic conditions. These results establish foldamer metallogels as a platform for multistimuli-responsive gels with independent control over their programmed gelation and disassembly using light and pH. We anticipate that our work will open up research in the area of rationally designed switchable metallogels, including tapping into the catalytic potential of Pd­(II) and stimuli-triggered guest capture/release for use in smart sensing and targeted small-molecule delivery vehicles.

## Supplementary Material



## Notes

Synthetic details; experimental procedures; data analysis of ^1^H and ^13^C NMR spectroscopy; mass spectrometry; melting point analysis and IR spectroscopy for all new compounds as a PDF; additional DNP spectra; UV–visible spectra; and details on gel optimization are also provided (PDF)

## §

Department of Solution-Processing of Hybrid Materials and Devices Helmholtz-Zentrum Berlin für Materialien and Energie GmbH, Kekuléstraße 5, 12489 Berlin, Germany
