# Supplementary material for: Dual Photo- and pH-Responsive Foldamer Metallogels
Source: J Am Chem Soc. 2026 Jul 30;148(32):34373–81. doi: 10.1021/jacs.6c07355 (PMC13495757; doi:10.1021/jacs.6c07355)
Supplement: Supplementary file 1 [file ja6c07355_si_001.pdf]

<u>Supporting Information</u>

Dual Photo- and pH-Responsive Foldamer Metallogels

Moosa Wasim[1], Benjamin M. Gallant[1,2], Neha Yadav,[1] Craig Fraser,[1] Sena Öztürk[1], Louise Male[1], Dominik J. Kubicki[1*], Maria C. Arno[1,3*], Sarah J. Pike[1*]

[1]School of Chemistry, University of Birmingham, Edgbaston, Birmingham, B15 2TT, UK

[2]Department of Solution-Processing of Hybrid Materials and Devices Helmholtz-Zentrum Berlin für Materialien und Energie GmbH, Kekuléstraße 5, 12489 Berlin, Germany

[3]Department of Cancer and Genomic Sciences, University of Birmingham, Edgbaston, Birmingham, B15 2TT

Email: s.j.pike@bham.ac.uk, m.c.arno@bham.ac.uk, d.j.kubicki@bham.ac.uk

Contents

Materials & Experimental methods 2
Abbreviations 2
Materials 2
Experimental procedures 3
Crystallographic details 12
General method for metallogel formation 13
Further investigation of gelation variables 13
Gel-sol-gel photo-switching and thermal healing 13
Hydrogel formation 13
Characterization methods 13
Supplementary Figures and Tables 16
Appendix A: Liquid-state ^{1}H, ^{13}C NMR spectra 54
References 70

Materials & Experimental methods

Abbreviations

The following abbreviations are employed: Ar = aromatic, Boc = *tert*-butyloxycarbonyl calcd. = calculated, CSA = chemical shift anisotropy, CP = cross polarisation, cryo-SEM = cryogenic scanning electron microscopy, decomp. = decomposed, DMF = dimethyl formamide, DMSO = dimethyl sulfoxide d = doublet, DNP = dynamic nuclear polarisation eq. = equivalent(s), g = gram(s), h = hour(s), Hz = Hertz, IR = infrared, LVER = linear viscoelastic region, MAS = magic angle spinning, m.p. = melting point, min = minute(s), MS = molecular sieves, nm = nano meters, ppm = parts per million, rt = room temperature, s = seconds, sat. = saturated, tetrachloroethane = 1,1,2,2-tetrachloroethane, wt. = weight percentage

Materials

O-phenylenediamine, trifluoroacetic acid, di-*tert*-butyl dicarbonate, pyridine, acetonitrile, methanol, dimethyl formamide, tetrachloroethane, acetone and chloroform were purchased from Thermofisher Scientific. Dichloromethane, dichloroethane, Oxone, pyridine,2,6-dicarbonyl chloride, tetramethyl ammonium hydroxide solution, DMSO, nitromethane, palladium tetrakisacetonitrile tetrafluoroborate, palladium tetrakisacetonitrile triflate and palladium bisacetonitrile dichloride were purchased from Sigma Aldrich. Ethyl acetate, petroleum ether and tetrahydrofuran were purchased from VWR. 4 Å molecular sieves and 3-amino pyridine were purchased from Acros Organics. Aniline was purchased from Alfa Aesar. All chemicals were used as received.

Experimental procedures

The synthetic procedure for foldamer synthesis is reported in detail in this section. Flash column chromatography was carried out using Fluorochem 40-63 µm 60Å silica gel. Melting points were obtained on a Gallenkemp melting point apparatus and are uncorrected. Mass spectra were obtained using a Waters Synapt (ES) spectrometer, fitted with a TOF detector. Infrared spectroscopy was carried out on a PerkinElmer Spectrum Two FT-IR spectrometer.

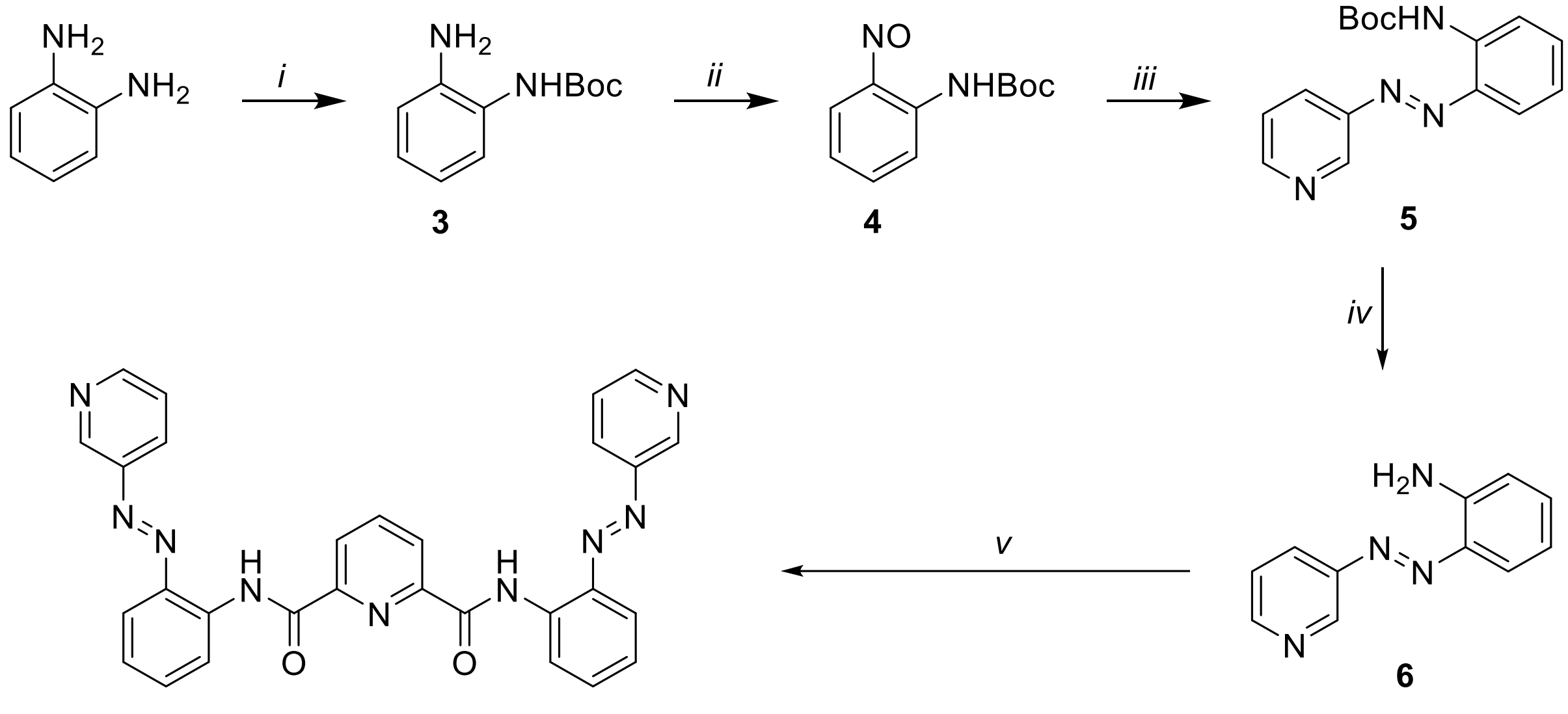

Scheme S1: Reagents and conditions. *i)* di-*tert*-butyl carbonate, tetrahydrofuran, rt, 16 h, 73 %; *ii)* Oxone, dichloromethane/water, rt, 1 h, 67 %; *iii)* 3-aminopyridine, 25 wt.% aqueous tetramethyl ammonium hydroxide, pyridine, 80 °C, 1 h, 84 %; *iv)* trifluoroacetic acid, dichloromethane, rt, 16 h, 87 %; *v)* 2,6-pyridine dicarbonyl chloride, pyridine, 4 Å MS, rt, 48 h, 85 %.

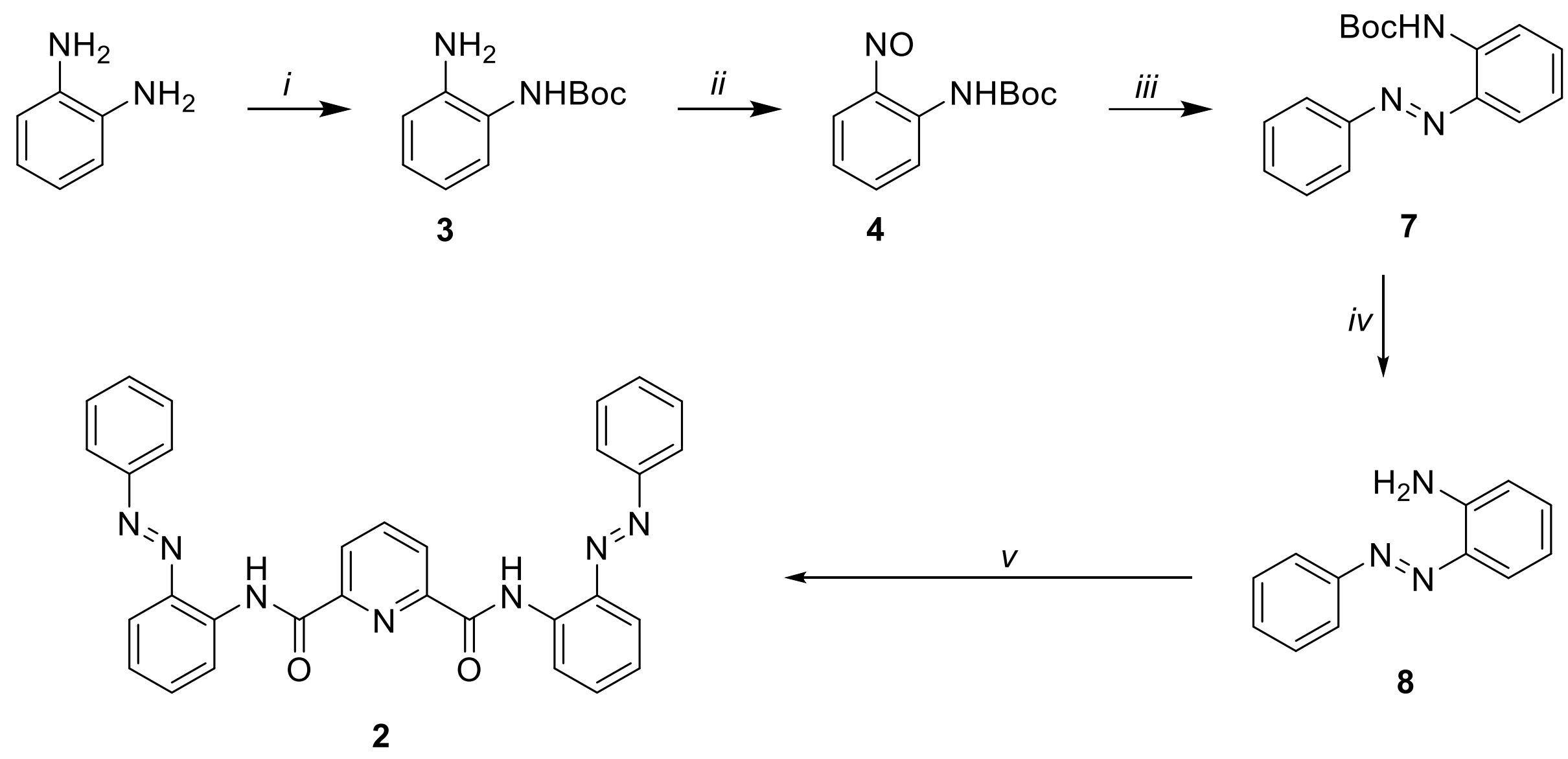

Scheme S2: Reagents and conditions. *i)* di-*tert*-butyl carbonate, tetrahydrofuran, rt, 16 h, 73 %; *ii)* Oxone, dichloromethane/water, rt, 1 h, 67 %; *iii)* aniline, 25 wt.% aqueous tetramethyl ammonium hydroxide, pyridine, 80 °C, 1 h, 36 %; *iv)* trifluoroacetic acid, dichloromethane, rt, 16 h, 98 %; *v)* 2,6-pyridine dicarbonyl chloride, pyridine, 4 Å MS, rt, 48 h, 49 %

3

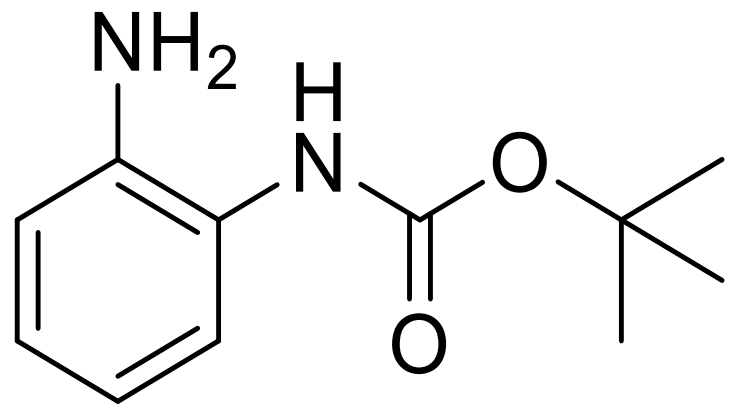

In a round-bottomed flask, *o*-phenylenediamine (5.0 g, 46 mmol) was added to tetrahydrofuran (50 mL) resulting in a pale-yellow solution. To this, di-*tert*-butyl dicarbonate (10 g, 46 mmol) was added dropwise in tetrahydrofuran (20 mL) over 16 h. The resultant pale-yellow solution was concentrated under reduced pressure to give crude solid product. The solid was dissolved in a minimum amount of ethyl acetate (20 mL), before the slow addition of petroleum ether (60 mL) in a layered crystallisation. The mixture was cooled to 5.0 °C and left for 16 h to give pale-yellow crystals. The crystals were washed with petroleum ether (3 × 10 mL) and dried *via* Buchner filtration to give **3** as pale-yellow crystals (7.0 g, 73%).
^{1}H NMR (400 MHz, $CDCl_3$, 298 K): δ 7.27 (d, *J* = 7.5 Hz, 1H, Ar*H*), 7.00 (td , *J* = 7.6, 1.5 Hz, 1H, Ar*H*), 6.82-6.74 (m, 2H, Ar*H*), 6.20 (s, 1H, N*H*), 3.73 (s, 2H, NH_2), 1.51 (s, 9H, CH_3); ^{13}C NMR (101 MHz, $CDCl_3$, 298 K) δ 153.6, 139.8, 126.0, 124.6, 119.5, 117.5, 80.4, 28.2; LR-ESIMS (+ve): 231.11 $[M+Na]^+$; HRESI(+ve): calcd. 231.1109 found 231.1113 for $C_{11}H_{16}N_2O_2Na$.

Characterisation data consistent with values reported in literature.[1]

4

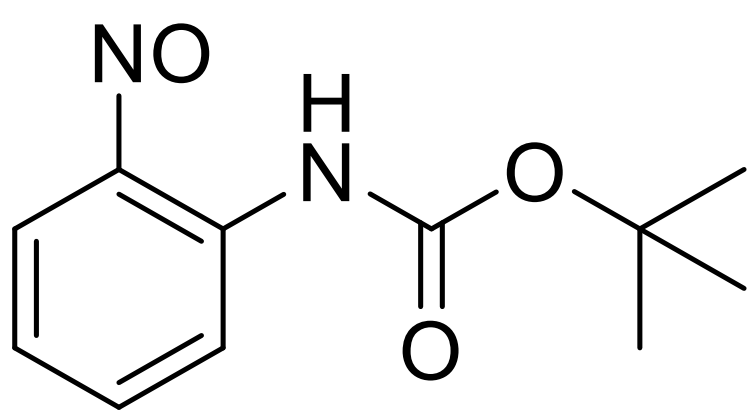

In a round-bottomed flask, **3** (2.0 g, 9.6 mmol) was added to dichloromethane (40 mL) resulting in a pale-yellow solution. To this, Oxone (12 g, 19 mmol) was added in water (100 mL) and left to stir for 1 h. The resultant greenish-black mixture was extracted with dichloromethane (3 × 30 mL) before being washed with 1 M aqueous HCl (2 × 30 mL) and sat. aqueous $NaHCO_3$ (1 × 15 mL). The organic layer was dried using $MgSO_4$, filtered and concentrated under reduced pressure to give the crude product as a brown oil. Purification by silica gel column chromatography (petroleum ether/dichloromethane, 70:30) gave **4** as a green oil (1.4 g, 67%).
^{1}H NMR (400 MHz, $CDCl_3$) δ 10.20 (s, 1H, N*H*), 8.59 (d, *J* = 8.6, 1H, Ar*H*), 7.66 (t, *J* = 8.7, 1H, Ar*H*), 7.37 (s, 1H, Ar*H*), 7.07 (t, *J* = 8.3, 1H, Ar*H*), 1.57 (s, 9H, CH_3); ^{13}C NMR (101 MHz, $CDCl_3$) δ 156.5, 152.5, 139.0, 121.4, 119.8, 82.0, 28.4.
LR-ESIMS (+ve): 222.10 $[M]^+$; HRESI(+ve): calcd. 222.0999 found 222.1003 for $C_{11}H_{14}O_3N_2$.

Characterisation data consistent with values reported in literature.[2]

5

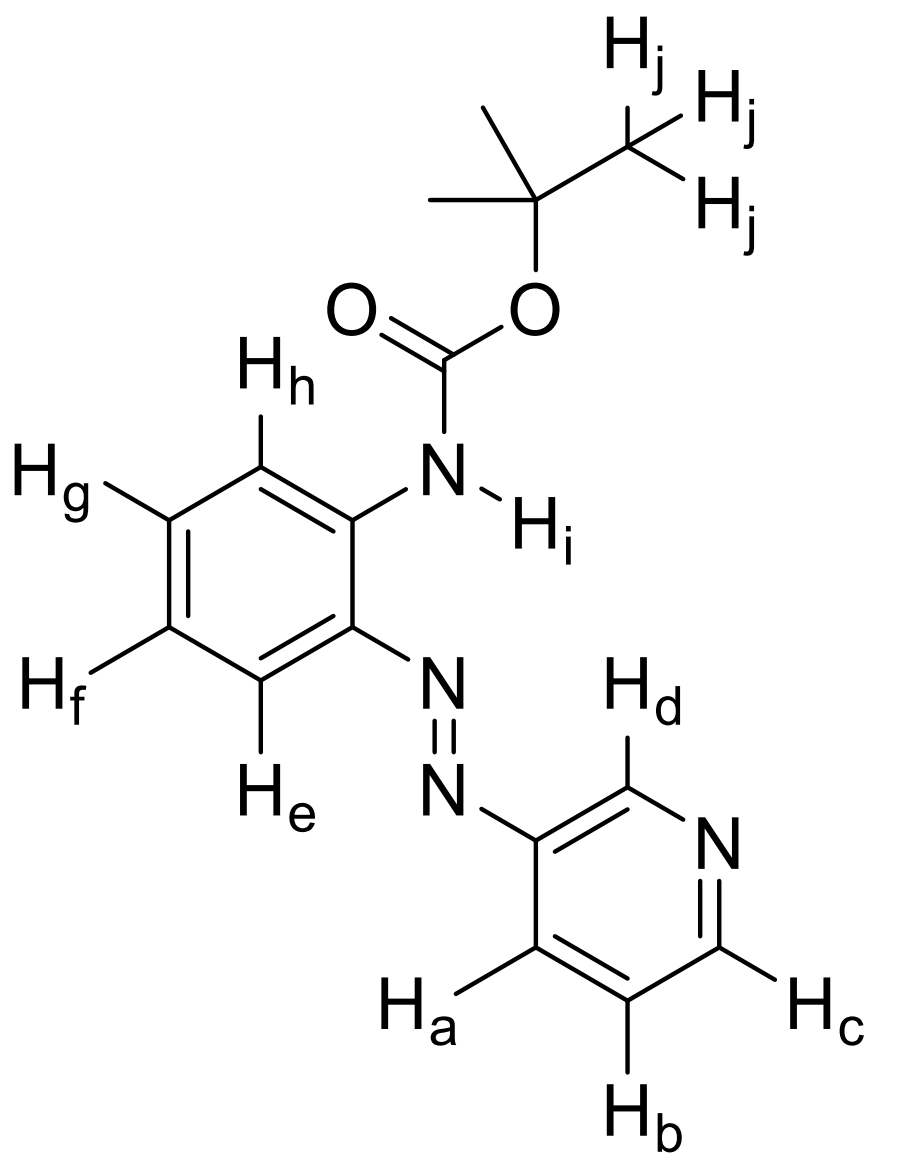

In a round-bottomed flask, 3-aminopyridine (0.30 g, 3.4 mmol) was added to pyridine (4.4 mL) resulting in a dark-yellow solution. To this was added 25 wt.% aqueous tetramethyl ammonium hydroxide (4.4 mL) to give a purple solution. To this, **4** (1.0 g, 4.5 mmol) in pyridine (7.0 mL) was added dropwise at 80 °C and left to stir for 1 h. The resultant brownish-black mixture was allowed to cool to room temperature and concentrated under reduced pressure before being extracted with dichloromethane (3 × 20 mL) and subsequently washed with brine (1 × 30 mL). The organic layer was dried using $MgSO_4$, filtered and concentrated under reduced pressure to give the crude product. Purification by silica gel column chromatography (petroleum ether/ethyl acetate, 50:50) gave **5** as a red oil (0.8 g, 84%).

^{1}H NMR (400 MHz, $CDCl_3$) δ 9.14 (2H, m, H_i and H_d), 8.71 (1H, dd, ^3J = 4.7, ^4J = 1.6 Hz, H_c), 8.41 (1H, dd, ^3J = 8.5, ^4J = 1.3 Hz, H_h), 8.11 (1H, ddd, ^3J = 8.2, ^4J = 2.4, 1.6 Hz, H_a), 7.83 (1H, ddd, ^3J = 8.2, ^4J = 1.6, H_e), 7.53 – 7.41 (2H, m, H_{b+g}), 7.09 (1H, ddd, ^3J = 8.4, 7.2, ^4J = 1.3 Hz, H_f), 1.57 (9H, s, H_j); ^{13}C NMR (101 MHz, $CDCl_3$) δ 152.5, 151.8, 147.9, 146.7, 138.9, 137.5, 133.9, 127.5, 124.2, 122.3, 120.4, 119.0, 28.5; IR (solid) ν_{max} 3409, 2977, 2930, 1728, 1590, 1509 cm^{-1}; LR-ESIMS (+ve): 299.15 $[M+H]^+$; HRESI(+ve): calcd. 299.1508 found 299.1512 for $C_{16}H_{19}N_4O_2$.

6

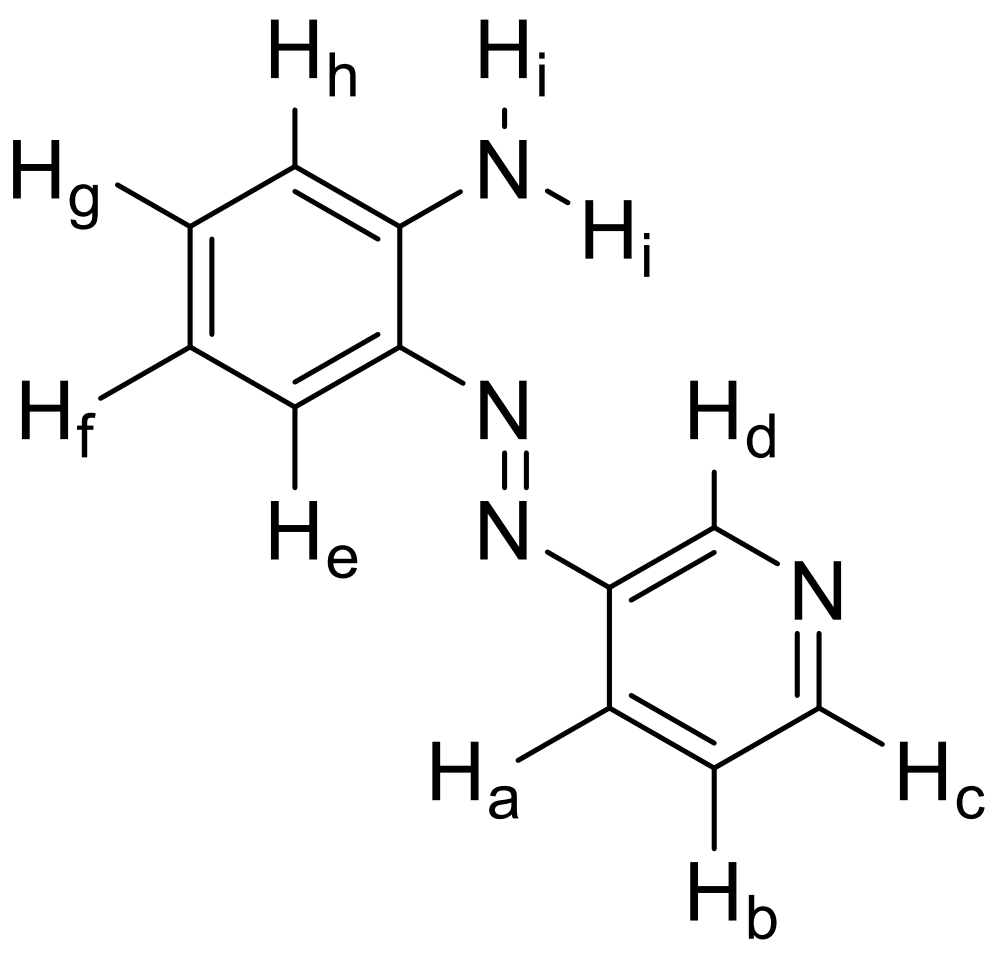

In a round-bottomed flask, **5** (1.3 g, 4.4 mmol) was added to dichloromethane (17 mL) resulting in an orange-red solution. To this was added trifluoroacetic acid (3.3 mL) and the mixture left to stir for 16 h. The resultant dark brown mixture was neutralised with 1 M aqueous sodium hydroxide (40 mL) and left to stir for a further 1 h to give an orange organic layer and a clear-orange aqueous layer. The organic layer was washed with water (1 × 50 mL) and brine (1 × 50 mL), dried using $MgSO_4$, filtered and concentrated under reduced pressure to give the product as a red solid (0.75 g, 87%).

m.p. 107-108 ºC (decomp.), ^{1}H NMR (400 MHz, $CDCl_3$, 298 K): δ 9.09 (1H, dd, ^4J = 2.5, 0.8 Hz, H_d), 8.64 (1H, dd, ^3J = 4.7, ^4J = 1.6 Hz, H_c), 8.08 (1H, ddd, ^3J = 8.2, ^4J = 2.4, 1.6 Hz, H_a), 7.84 (1H, dd, ^3J = 8.1 Hz, ^4J = 1.6 Hz, H_e), 7.42 (1H, ddd, ^3J = 8.2, ^4J = 4.7, ^5J = 0.8 Hz, H_b), 7.27 – 7.22 (1H, m, H_h), 6.83 (1H, ddd, ^3J = 8.3, 7.1, 1.3 Hz, H_g), 6.78 (1H, dd, ^3J = 8.2, 1.3 Hz, H_f), 6.00 (2H, s, H_i).^{13}C NMR (101 MHz, $CDCl_3$) δ 150.8, 148.4, 146.7, 143.1, 137.2, 133.2, 128.7, 126.5, 124.1, 117.6, 117.3; IR (solid) v_{max} 3363, 3126, 1617, 1582, 1550, cm^{-1}; LR-ESIMS (+ve): 198.09 $[M]^+$; HRESI(+ve): calcd. 198.0900 found 198.0901 for $C_{11}H_{10}N_4$.

1

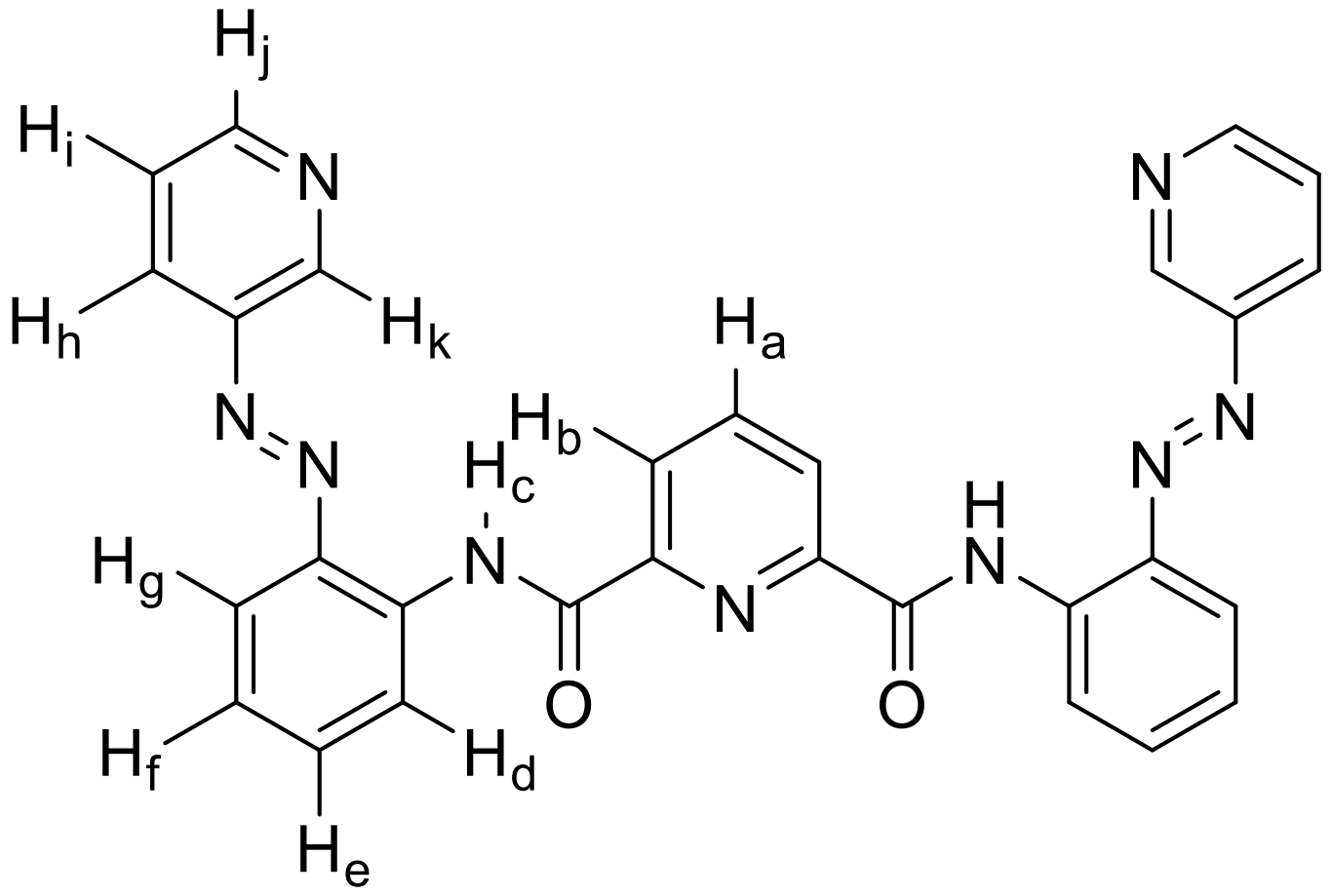

In a round-bottomed flask, **6** (0.75 g, 3.8 mmol) was added to dichloromethane (15 mL) resulting in an orange-red solution. To this was added pyridine (4.0 mL) and molecular sieves (0.25 g) and the solution left to stir for 0.5 h. 2,6-pyridine dicarbonyl chloride (0.37 g, 1.8 mmol) was then added, and the mixture left to stir for 66 h. Molecular sieves were removed via filtration, and the resulting orange mixture was diluted with dichloromethane (30 mL) before being washed with water (1 × 20 mL) and brine (1 × 20 mL). The organic layer was dried using $MgSO_4$, filtered and concentrated under reduced pressure to give the crude product as an orange solid. Purification by silica gel column chromatography (petroleum ether/ethyl acetate, 50:50) gave **1** as an orange solid (0.81 g, 85%).

210 - 211 ºC (decomp.), ^{1}H NMR (400 MHz, $CDCl_3$) δ 11.73 (s, 2H, H_c), 8.95 – 8.81 (m, 4H, H_k, H_j), 8.58 (d, ^3J = 7.8 Hz, 2H, H_b), 8.25 (t, ^3J = 7.7 Hz, 1H, H_a), 8.09 (dd, J = 4.7, 1.6 Hz, 2H, H_g), 7.67 (ddd, J = 8.2, 2.4, 1.6 Hz, 2H, H_h), 7.62 – 7.55 (m, 2H, H_d), 7.43 (dd, J = 8.1, 1.5, 2H, H_i), 7.12 (ddd, J = 8.1, 7.3, 1.3 Hz, 2H, H_f), 6.67 (ddd, J = 8.2, 4.8, 0.8 Hz, 2H, H_e); ^{13}C NMR (101 MHz, $CDCl_3$) δ 161.5, 151.7, 149.8, 148.3, 147.7, 140.1, 139.7, 136.8, 133.9, 125.9, 124.3, 124.1, 123.4, 120.2, 117.8; IR (solid) v_{max} 3326, 3050, 2937, 1686, 1510 cm^{-1}; LR-ESIMS (+ve): 528.19 [M+H]$^+$; HRESI(+ve): calcd. 528.1896 found 528.1896 for $C_{29}H_{22}N_9O_2$.

7

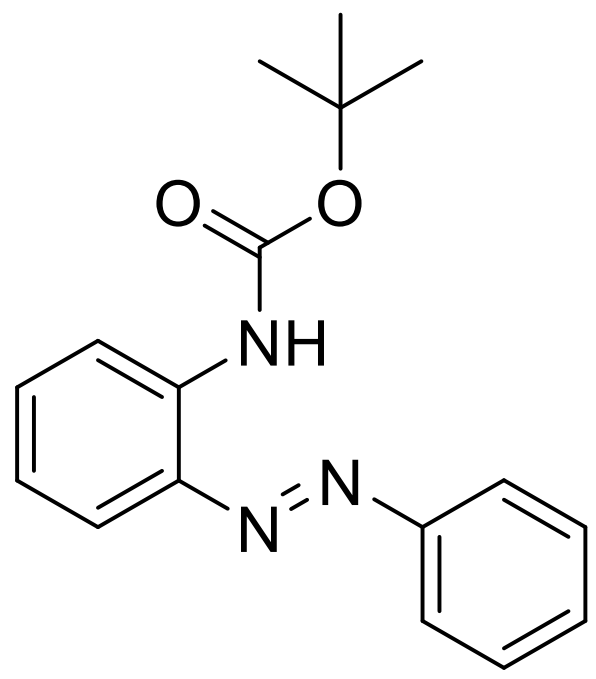

In a round-bottomed flask, aniline (0.16 g, 1.7 mmol) was added to pyridine (2.2 mL) resulting in a dark-yellow solution. To this was added 25 wt.% aqueous tetramethyl ammonium hydroxide (2.1 mL). To this, **4** (0.50 g, 2.3 mmol) in pyridine (3.5 mL) was added dropwise at 80 °C and left to stir for 1 h. The resultant brownish-black mixture was allowed to cool to room temperature and concentrated under reduced pressure before being extracted with dichloromethane (3 × 20 mL) and subsequently washed with brine (1 × 30 mL). The organic layer was dried using $MgSO_4$, filtered and concentrated under reduced pressure to give the crude product. Purification by silica gel column chromatography (petroleum ether/ethyl acetate, 95:5) gave **7** as an orange solid (0.18 g, 36%).
^{1}H NMR (400 MHz, $CDCl_3$) δ 9.25 (s, 1H, N*H*), 8.42 – 8.37 (m, 1H, Ar*H*), 7.91 – 7.85 (m, 2H, Ar*H*), 7.81 (dd, *J* = 8.1, 1.5 Hz, 1H, Ar*H*), 7.57 – 7.42 (m, 4H, Ar*H*), 7.09 (ddd, *J* = 8.5, 7.3, 1.3 Hz, 1H, Ar*H*), 1.57 (s, 9H, C*H*$_3$); ^{13}C NMR (101 MHz, $CDCl_3$) δ 152.7, 152.6, 138.9, 137.1, 132.9, 131.3, 129.4, 122.9, 122.2, 120.5, 118.9, 80.9, 28.5; LR-ESIMS (+ve): 198.10 [M-Boc+H]$^+$; HRESI(+ve): calcd. 198.1031 found 198.1048 for $C_{12}H_{12}N_3$.

Characterisation data consistent with values reported in literature.[3]

8

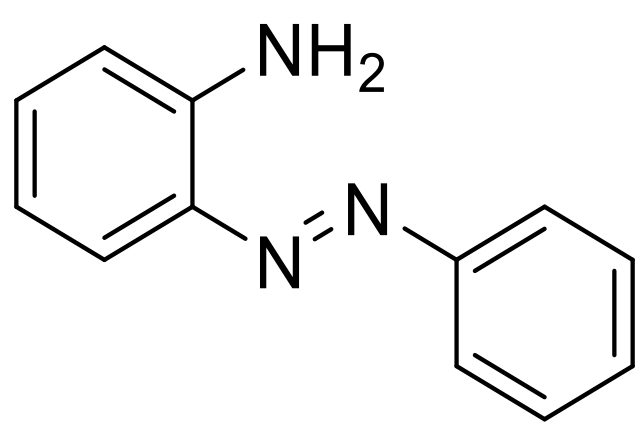

In a round-bottomed flask, **7** (0.14 g, 0.47 mmol) was added to dichloromethane (1.8 mL) resulting in an orange-red solution. To this was added trifluoroacetic acid (0.36 mL) and the mixture left to stir for 16 h. The resultant dark brown mixture was neutralised with 1 M aqueous sodium hydroxide (7 mL) and left to stir for a further 1 h to give an orange organic layer and a clear-orange aqueous layer. The organic layer was washed with water (1 × 20 mL) and brine (1 × 20 mL), dried using $MgSO_4$, filtered and concentrated under reduced pressure to give the product as a red solid (0.093 g, 98%).
^{1}H NMR (400 MHz, $CDCl_3$) δ 7.87 – 7.83 (m, 3H, Ar*H*), 7.53 – 7.47 (m, 2H, Ar*H*), 7.45 – 7.40 (m, 1H, Ar*H*), 7.25 – 7.19 (m, 1H, Ar*H*), 6.83 (ddd, *J* = 8.2, 7.1, 1.3 Hz, 1H, Ar*H*), 6.77 (dd, *J* = 8.2, 1.3 Hz, 1H, Ar*H*), 5.89 (s, 2H, N*H*); ^{13}C NMR (101 MHz, $CDCl_3$) δ 153.1, 143.1, 137.2,

132.4, 130.1, 129.2, 127.8, 122.3, 117.5, 117.1; LR-ESIMS (+ve): 198.10 $[M+H]^+$; HRESI(+ve): calcd. 198.1031 found 198.1109 for $C_{12}H_{12}N_3$.

Characterisation data consistent with values reported in literature.[4]

2

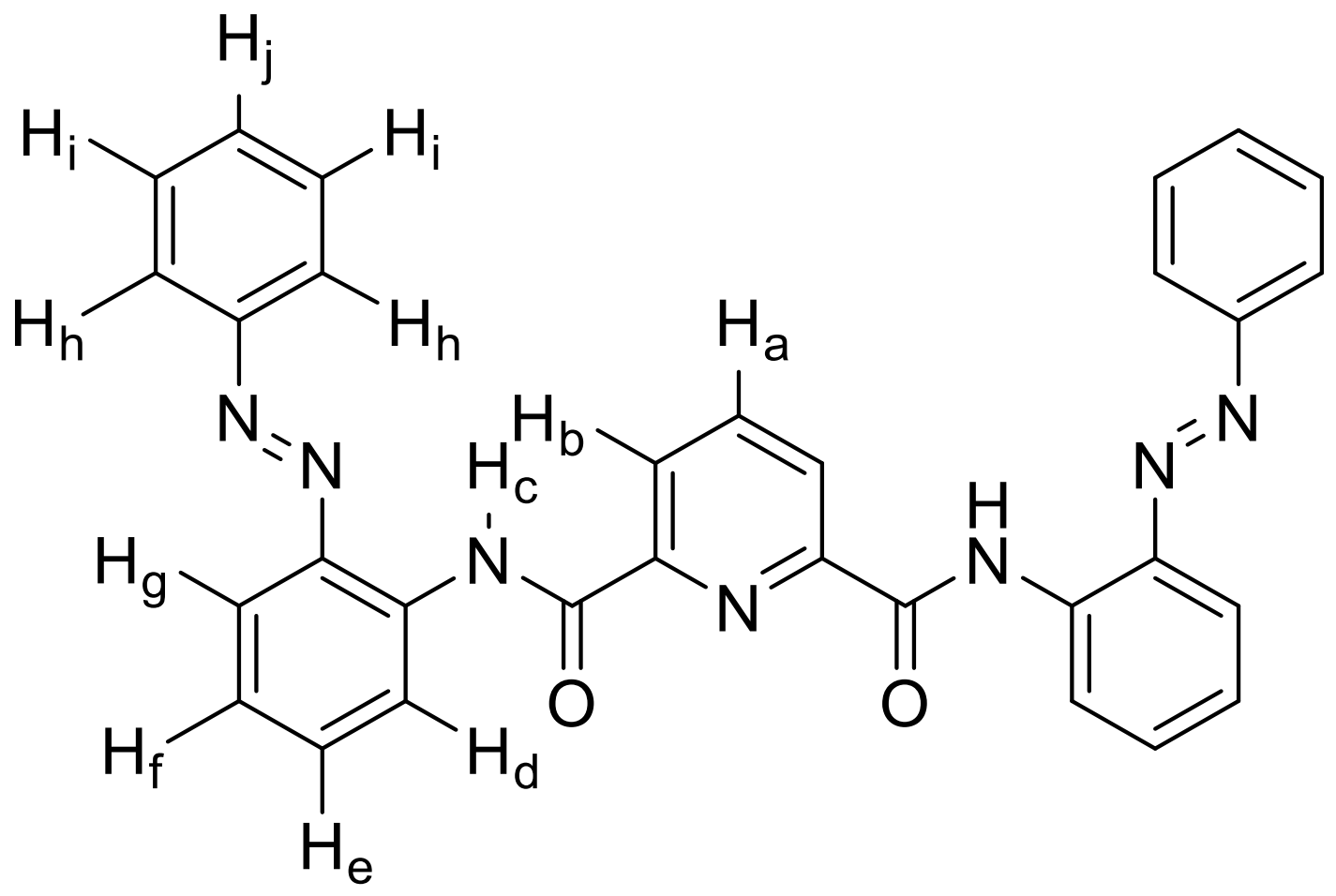

In a round-bottomed flask, **8** (0.093 g, 0.47 mmol) was added to dichloromethane (2 mL) resulting in an orange-red solution. To this was added pyridine (0.5 mL) and molecular sieves (0.1 g) and the solution left to stir for 0.5 h. 2,6-pyridine-dicarbonyl chloride (0.046 g, 0.23 mmol) was then added, and the mixture left to stir for 66 h. Molecular sieves were removed via filtration, and the resulting orange mixture was diluted with dichloromethane (10 mL) before being washed with water (1 × 10 mL) and brine (1 × 10 mL). The organic layer was dried using $MgSO_4$, filtered and concentrated under reduced pressure to give the crude product as an orange solid. Purification by silica gel column chromatography (petroleum ether/ethyl acetate, 80:20) gave **2** as an orange solid (0.058 g, 49%).

204 - 205 ºC (decomp.), ^{1}H NMR (400 MHz, $CDCl_3$) δ 11.88 (s, 2H, H_c), 8.87 (2H, dd, *J* = 8.3, 1.3 Hz, H_d), 8.56 (2H, d, *J* = 7.8 Hz, H_b), 8.22 (1H, t, *J* = 7.8 Hz, H_a), 7.57 – 7.50 (6H, m, H_g, H_h), 7.44 (2H, dd, *J* = 8.1, 1.5 Hz, H_e), 7.09 (2H, ddd, *J* = 8.3, 7.3, 1.3 Hz, H_f), 6.97 – 6.85 (6H, m, H_i, H_j); ^{13}C NMR (101 MHz, $CDCl_3$) δ 161.7, 152.3, 150.1, 139.9, 139.8, 136.3, 132.7, 130.9, 128.7, 125.8, 123.9, 122.3, 120.1, 118.3; IR (solid) ν_{max} 3350, 3331, 2920, 1687, 1587, 1516 cm^{-1}; LR-ESIMS (+ve): 528.19 $[M+H]^+$; HRESI(+ve): calcd. 526.1992 found 526.1962 for $C_{29}H_{24}N_9O_2$.

Crystallographic details

The datasets for **1** and **2** were measured on a Rigaku XtaLAB Synergy diffractometer using a HyPix detector. The data collections were driven and processed and absorption corrections were applied using CrysAlisPro.[5] Crystals of **1** were grown via slow evaporation of a solution of tetrachloroethane. Crystals of **2** were grown via slow evaporation of a solution of dichloromethane.
Using OLEX2, the structures were solved using ShelXT and were refined by a full-matrix least-squares procedure on F^2 in ShelXL.[6–8] All non-hydrogen atoms were refined with anisotropic displacement parameters. Hydrogen atom treatment is discussed for each structure individually. CCDC 2494187 (**1**) and 2494186 (**2**) contain the supplementary crystallographic data for this paper. These data can be obtained free of charge from The Cambridge Crystallographic Data Centre via www.ccdc.cam.ac.uk/data_request/cif.

Details for **1**: $C_{31}H_{23}Cl_4N_9O_2$ (*M* =695.38 g/mol): monoclinic, space group $P2_1/c$ (no. 14), *a* = 12.84570(10) Å, *b* = 10.30800(10) Å, *c* = 23.5404(2) Å, *β* = 97.2280(10)°, *V* = 3092.30(5) Å^3, *Z* = 4, *T* = 100.00(10) K, μ(Cu Kα) = 3.871 mm^{-1}, *Dcalc* = 1.494 g/cm^3, 29071 reflections measured (6.936° ≤ 2Θ ≤ 156.528°), 6034 unique (R_{int} = 0.0312, R_{sigma} = 0.0257) which were used in all calculations. The final R_1 was 0.0319 (I > 2σ(I)) and wR_2 was 0.0845 (all data).
The ring system C(25)-C(26)-C(27)-C(28)-N(9)-C(29) / C(25)-C(26A)-C(27A)-C(28A)-N(9A)-C(29A) is disordered over two positions at a refined percentage occupancy ratio of 82.0 (10): 18.0 (10).
The structure contains a molecule of solvent.
The hydrogen atoms bonded to N(2) and N(6) were located in the electron density and refined freely. All remaining hydrogen atoms were fixed as riding models with the isotropic thermal parameters (U_{iso}) based on the U_{eq} of the parent atom.

Details for **2**: $C_{31}H_{23}N_7O_2$ (*M* =525.56 g/mol): triclinic, space group P-1 (no. 2), *a* = 10.3628(2) Å, *b* = 12.1885(3) Å, *c* = 12.2370(3) Å, *α* = 108.610(2)°, *β* = 114.041(2)°, *γ* = 98.826(2)°, *V* = 1263.97(6) Å^3, *Z* = 2, *T* = 100.00(10) K, μ(Cu Kα) = 0.731 mm^{-1}, *Dcalc* = 1.381 g/cm^3, 23482 reflections measured (8.1° ≤ 2Θ ≤ 156.356°), 4990 unique (R_{int} = 0.0322, R_{sigma} = 0.0235) which were used in all calculations. The final R_1 was 0.0450 (I > 2σ(I)) and wR_2 was 0.1187 (all data).
The hydrogen atoms bonded to N(1) and N(5) were located in the electron density and refined freely. All remaining hydrogen atoms were fixed as riding models with the isotropic thermal parameters (U_{iso}) based on the U_{eq} of the parent atom.

General method for metallogel formation

1 (15 mg, 0.028 mmol) was weighed out into a glass vial and dissolved in 350 µL tetrachloroethane. To this was added palladium tetrakisacetonitrile tetrafluoroborate (6.3 mg, 0.014 mmol) in 150 µL acetonitrile. The mixture is shaken vigorously before being left in the dark to form a gel.

Further investigation of gelation variables

A 1:1 ratio of the foldamer to palladium tetrakisacetonitrile tetrafluoroborate produced a highly viscous precipitate while a 3:1 ratio was found to form a metallogel overnight. Ratios of 4:1, 5:1 and 6:1 failed to produce gels entirely (**Table S2, Figure S22**). It can be inferred that there exists some sensitivity of the metallogel to foldamer: metal stoichiometry, suggesting that network formation is driven by a particular mode of complexation between the foldamer and palladium(II) from which significant deviation no longer results in gelation.[9,10] Higher temperatures were found to result in quicker gelation, highlighting a simple kinetic dependency in the complexation of the foldamer to palladium(II) (**Table S3, Figure S21**).Varying the counter-ion for palladium tetrakisacetonitrile to either triflate or hexafluoroantimonate anions resulted in gels visually identical to their tetrafluoroborate analogs (**Figures S22-S23**). However, an attempt to form a metallogel with a hexafluorophosphate anion was unsuccessful (**Figure S24**). The use of DMSO was necessitated for the hexafluoroantimonate and hexafluorophosphate trials due to the insolubility of these salts in acetonitrile. The hexafluorophosphate salt was found to be only sparingly soluble in DMSO, and so the failure of this salt to form an metallogel in comparison to its tetrafluoroborate equivalent may therefore be attributed to solubility effects which prevent effective nucleation and growth of the network due to a lack of available Pd(II) in solution. Syntheses of palladium tetrakisacetonitrile hexafluoro antimonate and palladium tetrakisacetonitrile hexafluoro phosphate were performed according to a known literature protocol.[9]

Gel-sol-gel photo-switching and thermal healing

An metallogel sample was formed in a test tube and lowered into a glass dish wrapped in foil, with the inside lined with 395 nm LED lights. The setup was cooled using a desk fan and covered with an opaque box. To reform the metallogel, the test tube is placed in a sand bath at 60 °C for 2 h.

Hydrogel formation

An metallogel sample was formed in a 5 mL plastic syringe. The syringe plunger was removed, and the ends of the syringe were cut off with scissors. The metallogel disk was then pushed out into a glass beaker with a spatula before being immersed in methanol. The methanol is replenished twice a day for three days, followed by immersion of the metallogel disk in water for a further three days, with the water again replenished twice a day to give a hydrogel.

Characterization methods

Liquid-state NMR spectroscopy

All liquid-state NMR spectroscopy was carried out on a Bruker AVIII400 or Bruker NEO400 NMR spectrometer using the residual solvent as the internal standard. All $CDCl_3$ used contains 0.03% TMS. All the chemical shifts (δ) are quoted in ppm and coupling constants are given in

Hz and are rounded to 0.1 Hz. Splitting patterns are abbreviated as follows: s (singlet), d (doublet), t (triplet), m (multiplet) or some combination of these.

MAS DNP NMR Spectroscopy

Solid-state magic angle spinning (MAS) dynamic nuclear polarization (DNP) nuclear magnetic resonance (NMR) spectroscopy was carried out using a commercial Bruker Avance III 400 MHz spectrometer equipped with a 3.2 mm Bruker LTMAS HXY probe. All measurements were conducted at approximately 100 K. For both ^{13}C and ^{15}N measurements a ^{1}H-X cross-polarisation (CP) MAS pulse sequence was used. A solid sample of neat ^{13}C,^{15}N-enriched γ-glycine at 100 K was used to calibrate the ^{1}H, ^{13}C and ^{15}N radiofrequency field amplitudes (60, 40 and 35 kHz, respectively) and CP contact time (2 ms and 8 ms for ^{1}H-^{13}C and ^{1}H-^{15}N, respectively), and to reference ^{13}C and ^{15}N chemical shifts (174.9 ppm for ^{13}C; 32.9 ppm for ^{15}N). ^{1}H decoupling at an RF field of 60 kHz was used during acquisition in all measurements. Immediately prior to each DNP NMR measurement, approximately 10 µl of a 16 mM solution of TEKPol (Cortecnet, C020P005) in 1,1,2,2-tetrachloroethane was introduced into the metallogel sample (approximately 60 µl) using incipient wetness impregnation (IWI).[10] DNP enhancement factors ($\varepsilon_{C\ CP}$) were calculated by taking the intensity ratio of the ^{13}C CP MAS foldamer signals in the 115-125 ppm range with microwaves on and off, accounting for the difference in the number of scans (**Figure *S33-S37***).

We also attempted to directly incorporate TEKPol into the gel network at 16 mM concentration (optimal for polarization transfer using TEKPol/TCE) during initial gel synthesis.[10,11] This proved successful but provided DNP enhancements that were somewhat lower ($\varepsilon_{C\ CP}$ = 5-10) than those achievable *via* IWI. We therefore proceeded with the IWI approach throughout.[10,11]

To assess whether the structure of the gel is affected by freezing, we have made the following observations: (a) the gel freezes very rapidly, within less than 1 second, resulting in vitrification of the solvent matrix, as evidenced by the lineshape of the ^{13}C signal of TCE in fig. S39 (microwave ON spectrum); it shows a single broad peak, with no discernible ^{13}C-$^{35/37}Cl$ residual dipolar coupling that appears when TCE crystallizes (ref. 10.1039/D3CP04271E). This is important because crystallization could conceivably disrupt the gel, whereas amorphous glass is less likely to do so; (b) the gel appears intact after thawing at RT; and (c) the gel can be frozen and thawed multiple times and the ^{13}C DNP-enhanced spectrum remains unchanged (fig. S44). This observation is consistent with the high stability of pyridine-Pd^{2+} coordination, with a bond dissociation enthalpy of ca. 100 kJ/mol, ref. 10.1016/j.molstruc.2023.136678.
To demonstrate this point further, we show ^{1}H-^{13}C CP MAS DNP NMR spectra of a sample of pristine organogel frozen to 100 K, thawed, and frozen at 100 K again (fig. S44).[12]

UV/Vis spectroscopy

UV/Vis spectroscopic studies were carried out Shimadzu UV3600i UV/Vis photo-spectrometer fitted with a temperature control unit. For metallogel samples, an metallogel was spread thinly on a glass slide and covered with a small plastic sheet before being placed inside the spectrometer for analysis. These were recorded in triplicate and plotted as a mean to account for experimental variability.

Rheology

Oscillatory rheological measurements both frequency sweep and amplitude sweeps were performed on an Anton Paar MCR302 rheometer equipped with an 8 mm parallel plate geometry and a Peltier-controlled heating system for precise temperature regulation. Hydrogel samples (3 wt%, 4 wt%, and 5 wt%) were prepared as discs with a height of 1 mm and loaded onto the base plate for analysis. To determine the Linear Viscoelastic Region (LVER), amplitude sweep tests were conducted where the storage modulus (*G'*) and loss modulus (*G''*) were measured as a function of shear strain ranging from 0.1% to 10% at 25 °C temperature.

Following the identification of the LVER, frequency sweep analyses were performed to evaluate the stability and elasticity of the network. These tests were conducted over an angular frequency range of 0.1 to 100 rad/s at a constant strain (1%) within the LVR, ensuring the structural integrity of the gels remained intact throughout the measurement. Gelation was confirmed for all concentrations by the observation of storage and loss moduli across the entire frequency range.

Scanning Electron Microscopy

Cryogenic scanning electron microscopy (cryo-SEM) was performed on a FEI FIB Quanta 3D SEM equipped with a cold stage and a Quorum PP3010 sample preparation chamber. The hydrogel sample packed into a shuttle rivet and shuttle was frozen in liquid nitrogen (-196 °C). The shuttle was then transferred to the sample preparation chamber (at -140 °C) connected to the SEM chamber. The frozen sample was surface fractured and sublimed at -110 °C over 10 min to reveal the cross-sectional surface. The temperature was then brought back down to -140 °C and the sample was sputter coated with platinum before being transferred under vacuum into the SEM chamber, which was kept at −140 °C for imaging. The accelerating voltage was set at 5 kV.

Supplementary Figures and Tables

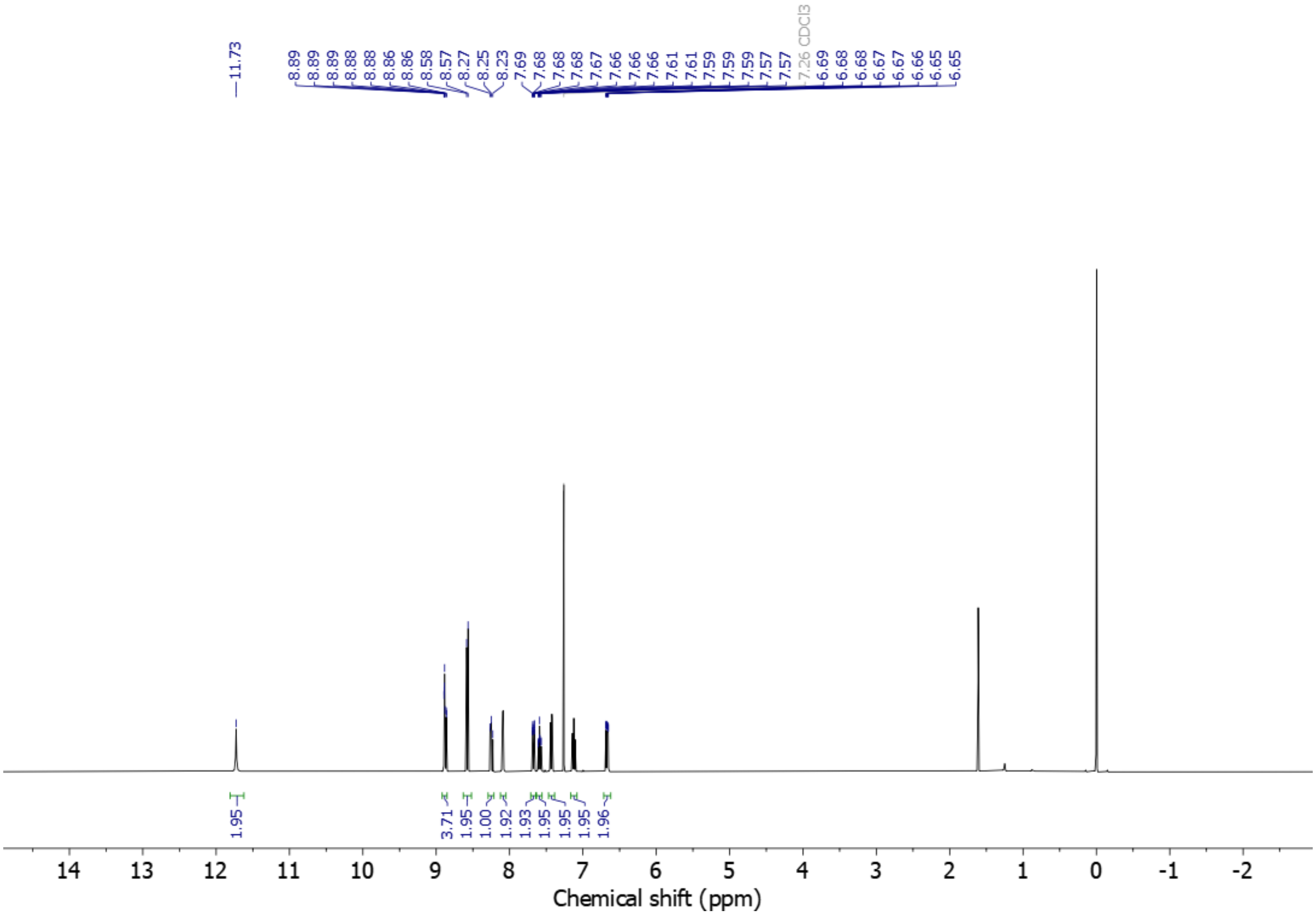

Figure S1: ^{1}H NMR spectrum of **1** (400 MHz, $CDCl_3$, 298 K).

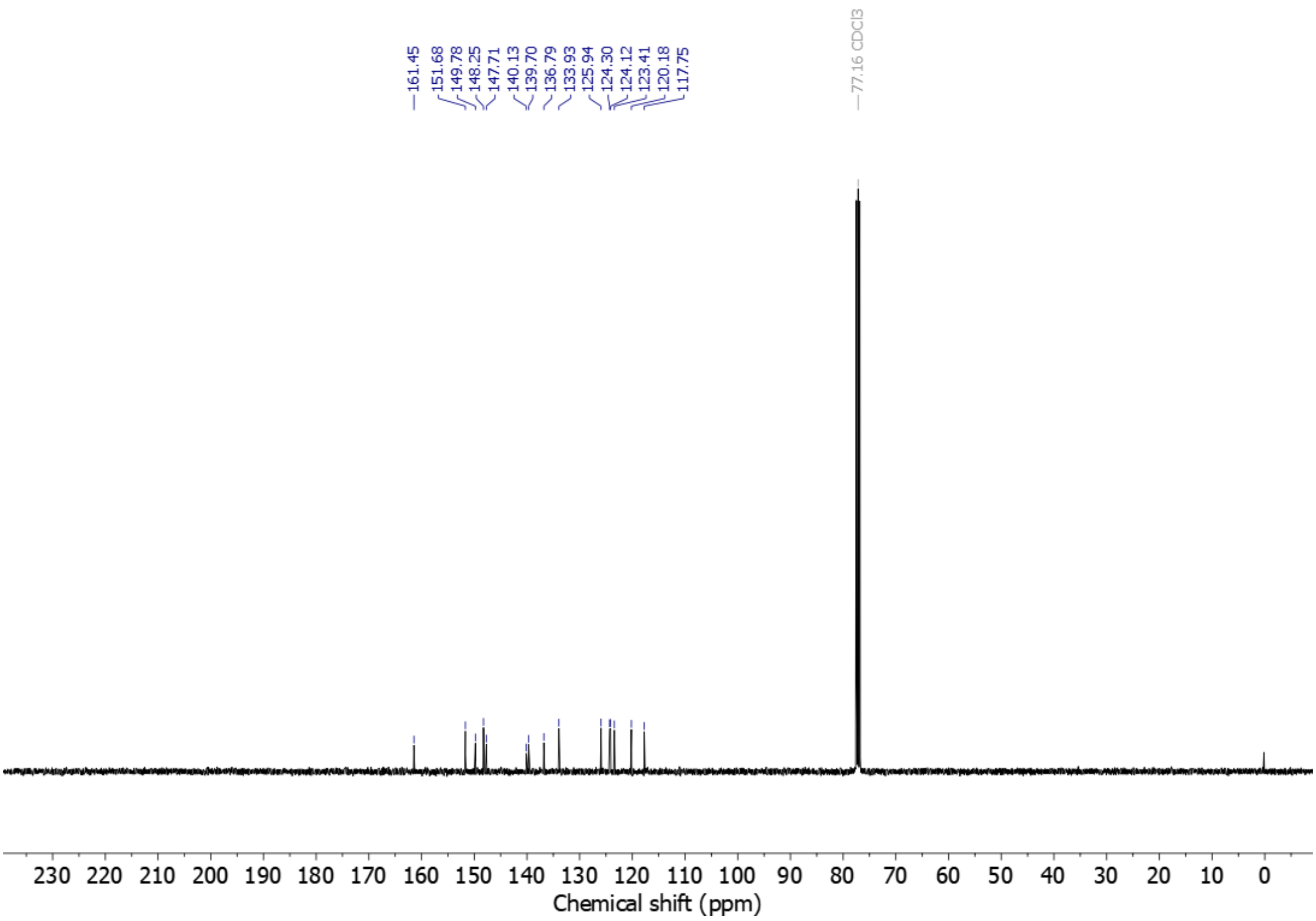

Figure S2: ^{13}C NMR spectrum of **1** (400 MHz, $CDCl_3$, 298 K).

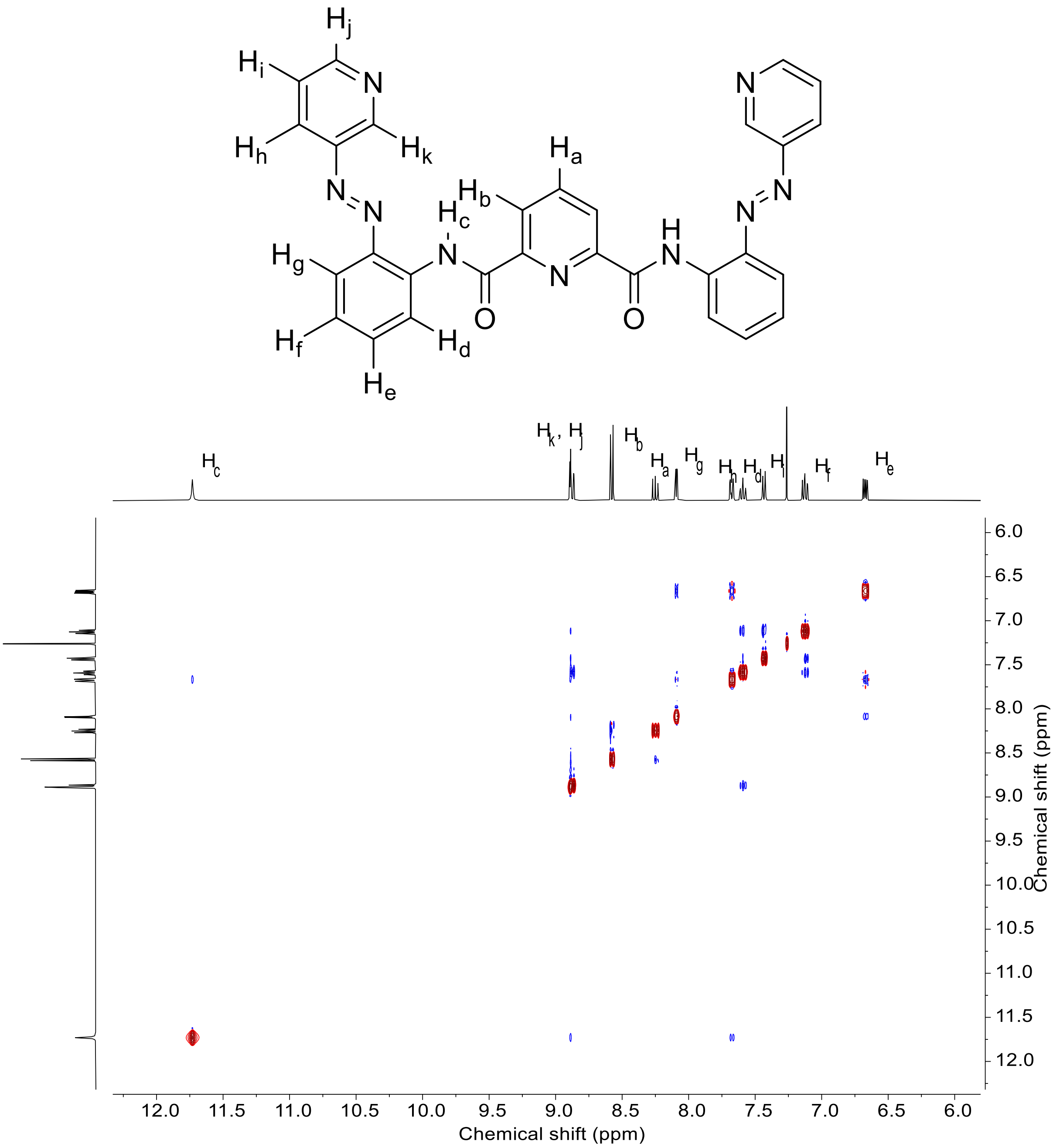

Figure S3: Partial NOESY NMR spectrum of **1** (400 MHz, $CDCl_3$, 298 K). Correlation between protons H_c and H_h indicate the helical *syn*-*syn* conformation.

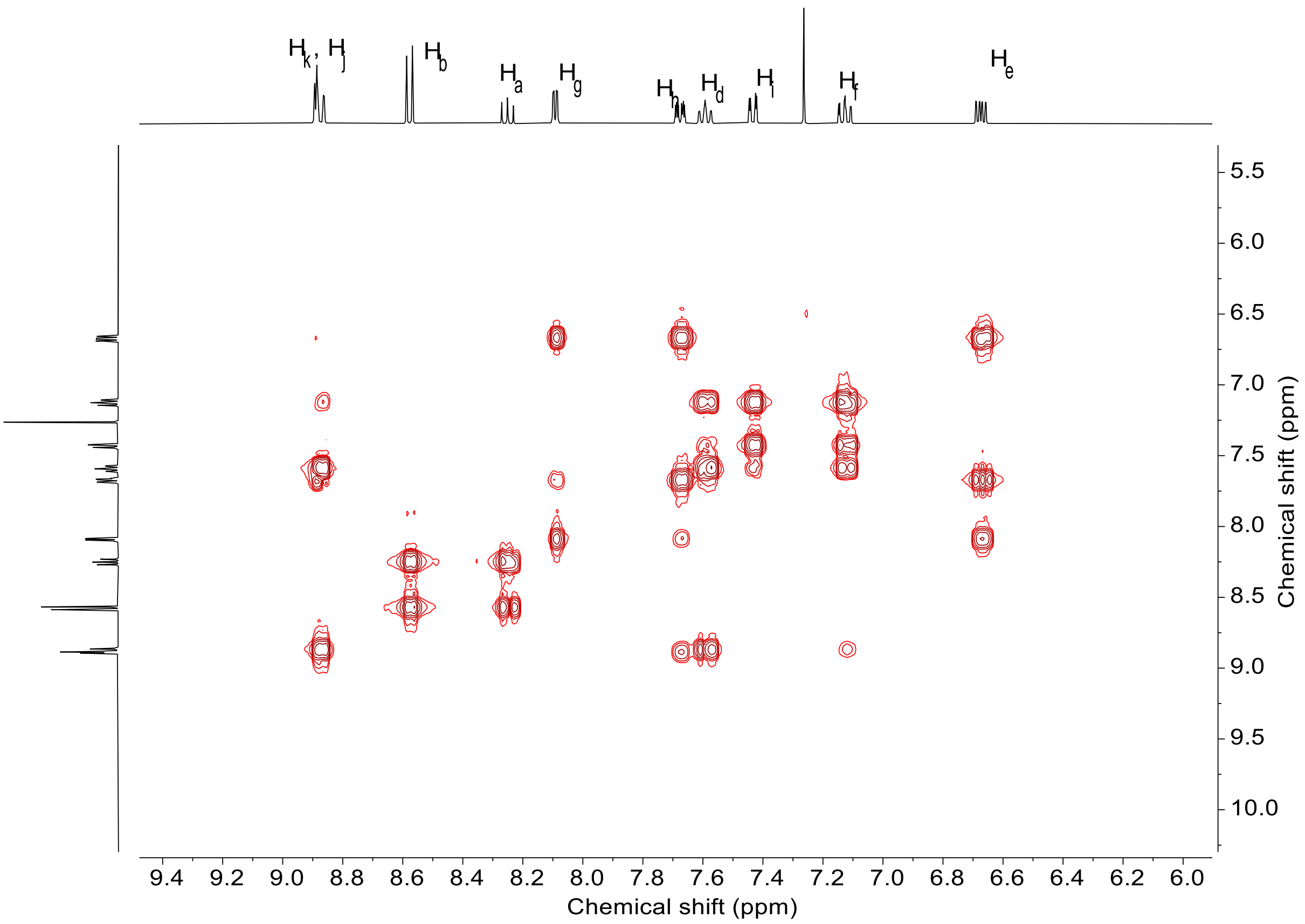

Figure S4: Partial COSY NMR spectrum of **1** (400 MHz, $CDCl_3$, 298 K).

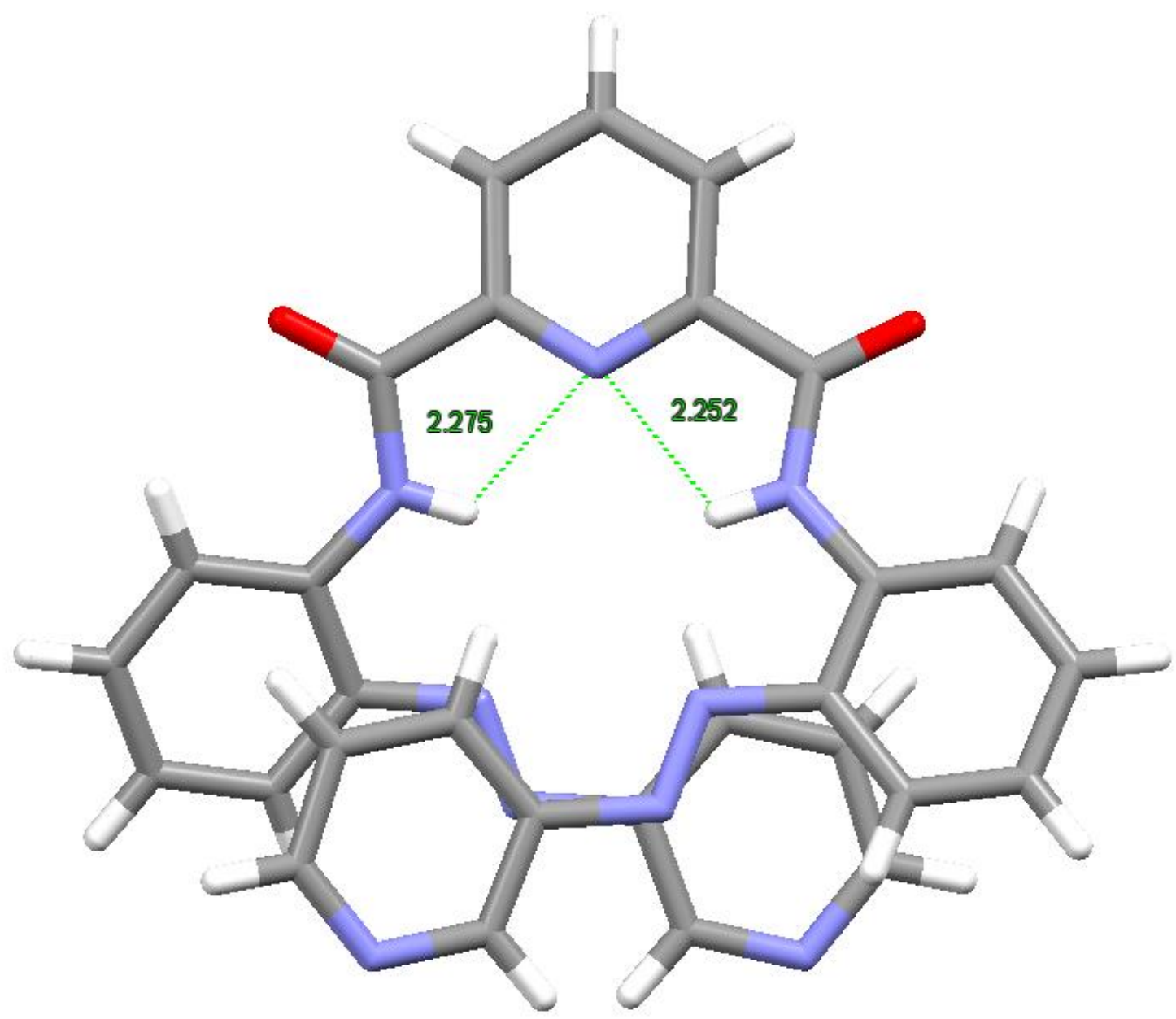

Figure S5: Crystal structure of **1** showing the intramolecular bifurcated hydrogen bonding motif between the N atom of the central pyridine unit and adjacent amide N-H's.

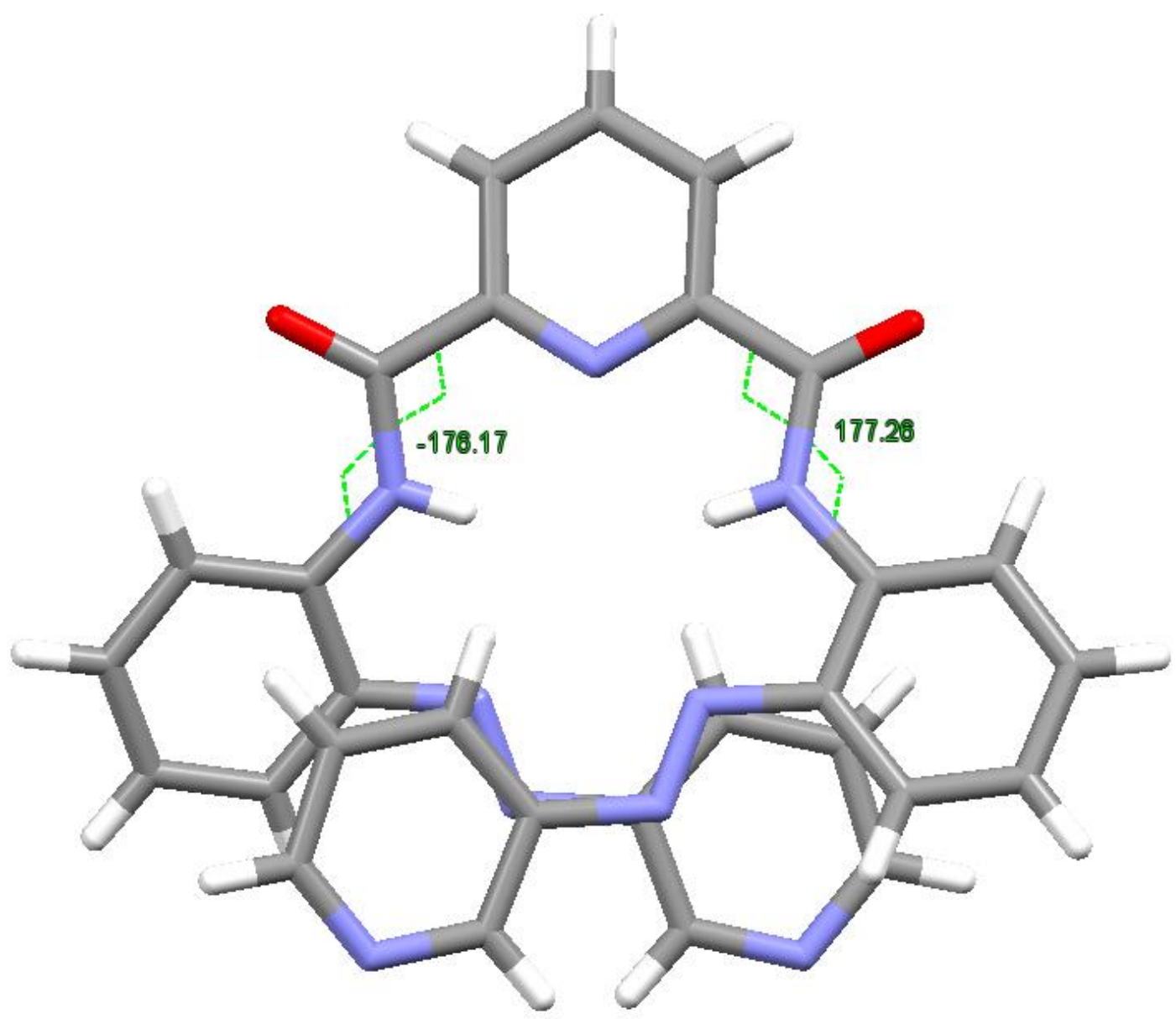

Figure S6: Crystal structure of **1** showing the torsion angles of the internal peptide bonds.

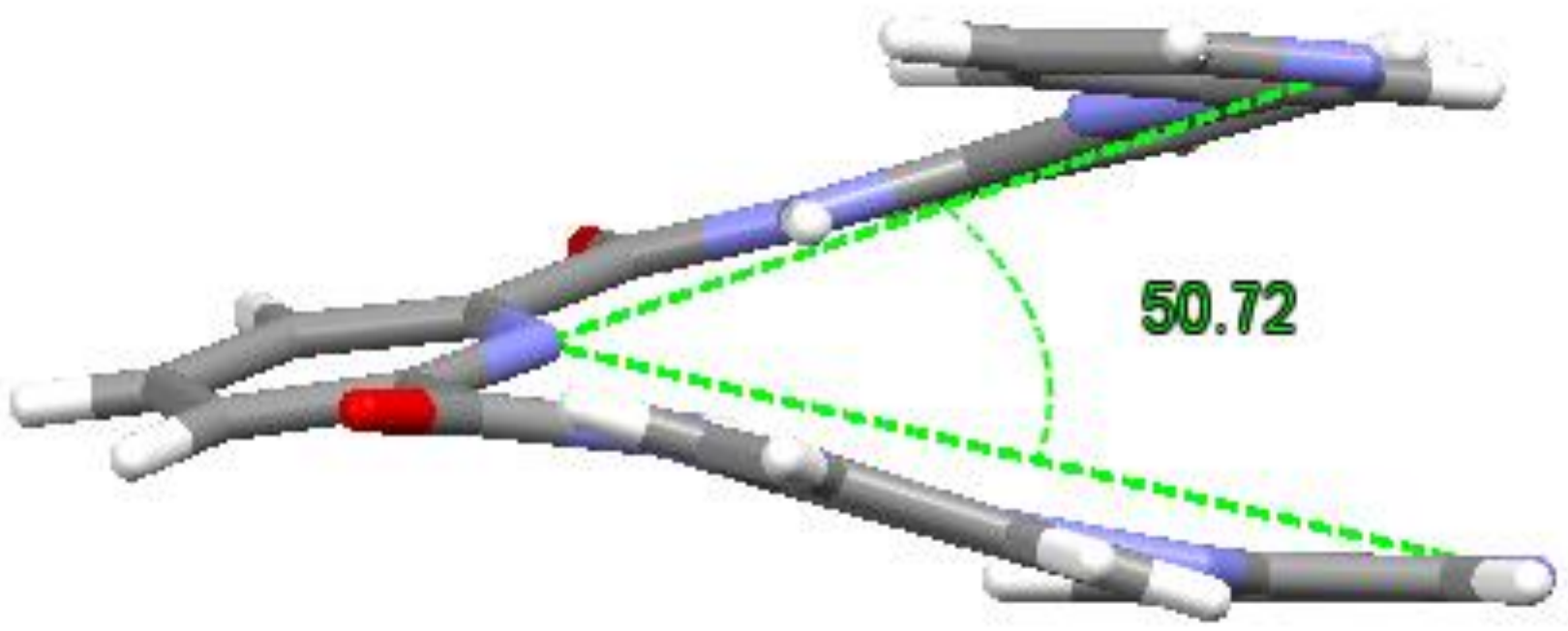

Figure S7: Crystal structure of **1** showing an approximate bite angle of 50 ° between the pyridyl nitrogen atoms in the solid state. While other conformations are theoretically possible upon palladium(II) coordination, none may reasonably reach the 90 ° required for four-fold monodentate coordination of two foldamers to a single palladium(II) center.

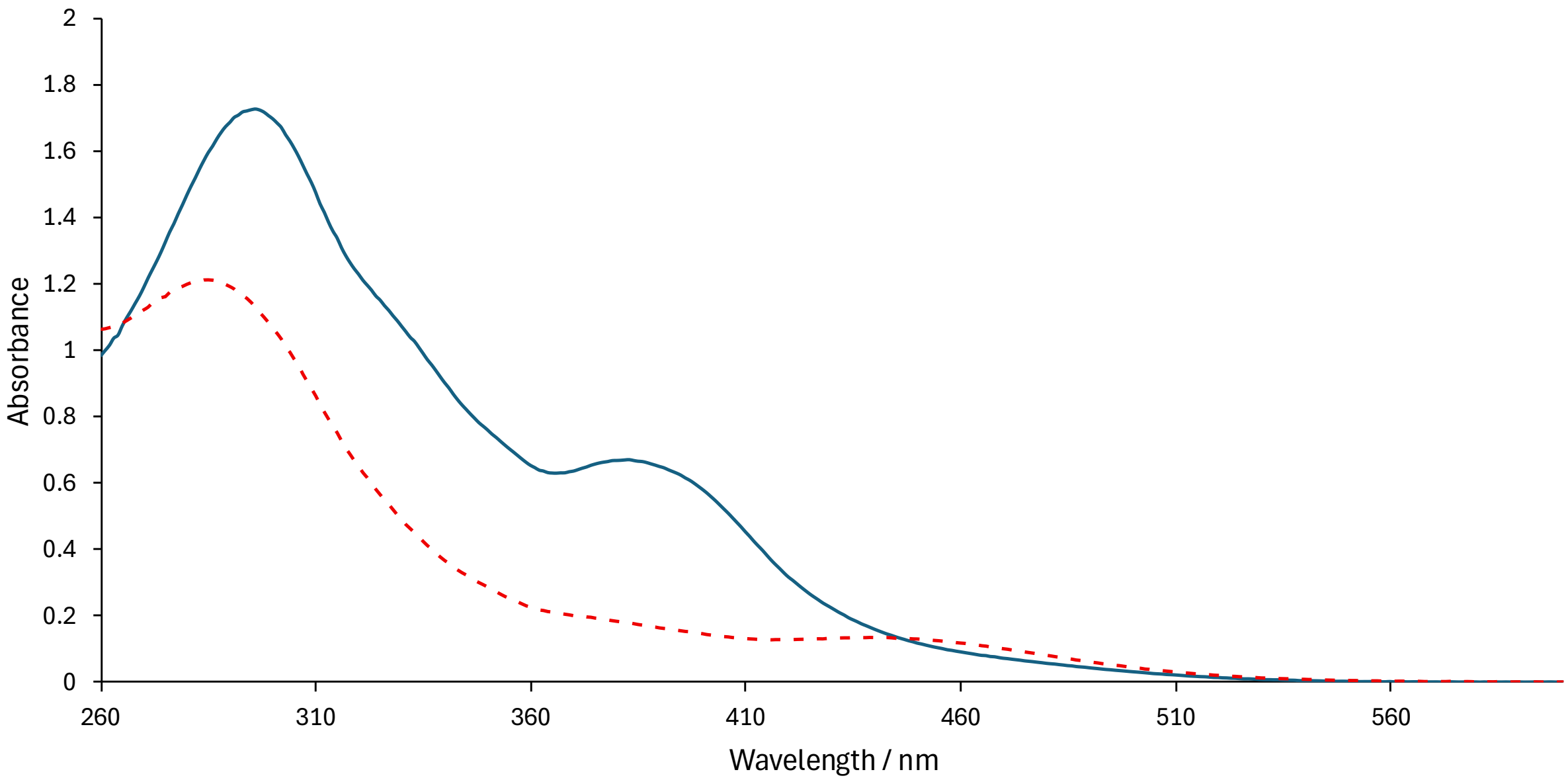

Figure S8: UV/Vis spectrum of a 6 × 10^{-5} M 3:7 v/v acetonitrile:tetrachloroethane solution of **1** before UV illumination (shown in blue line) and at the photostationary state (shown in the dashed red line) after UV illumination at 395 nm for 25 s.

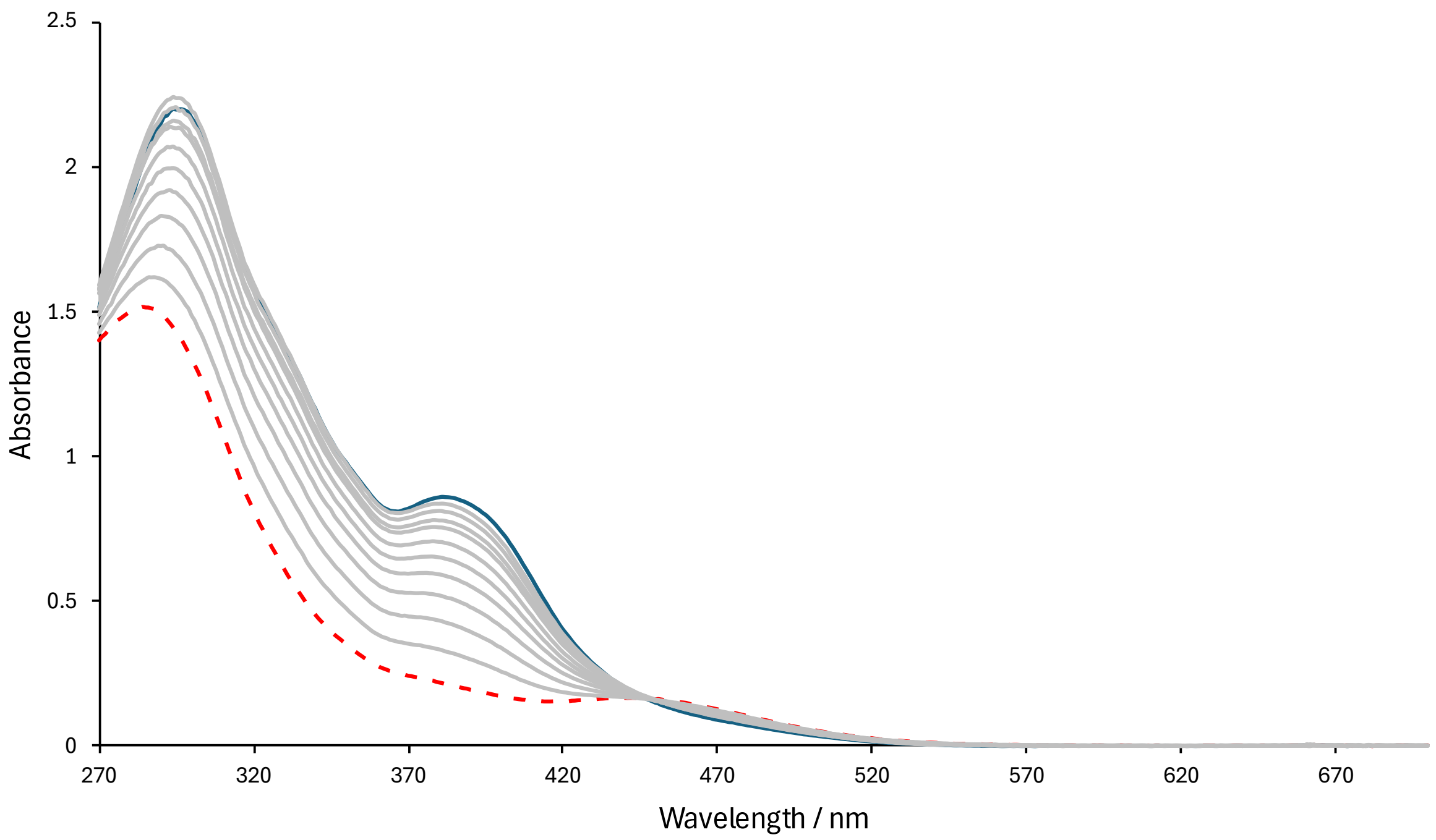

Figure S9: UV/Vis spectrum of a 6 × 10^{-5} M acetonitrile solution of **1** before UV illumination (shown in blue line) and at the photostationary state (shown in the dashed red line) after UV illumination at 395 nm for 25 s. Grey lines represent spectra recorded up to 10 h after UV illumination at 40 °C.

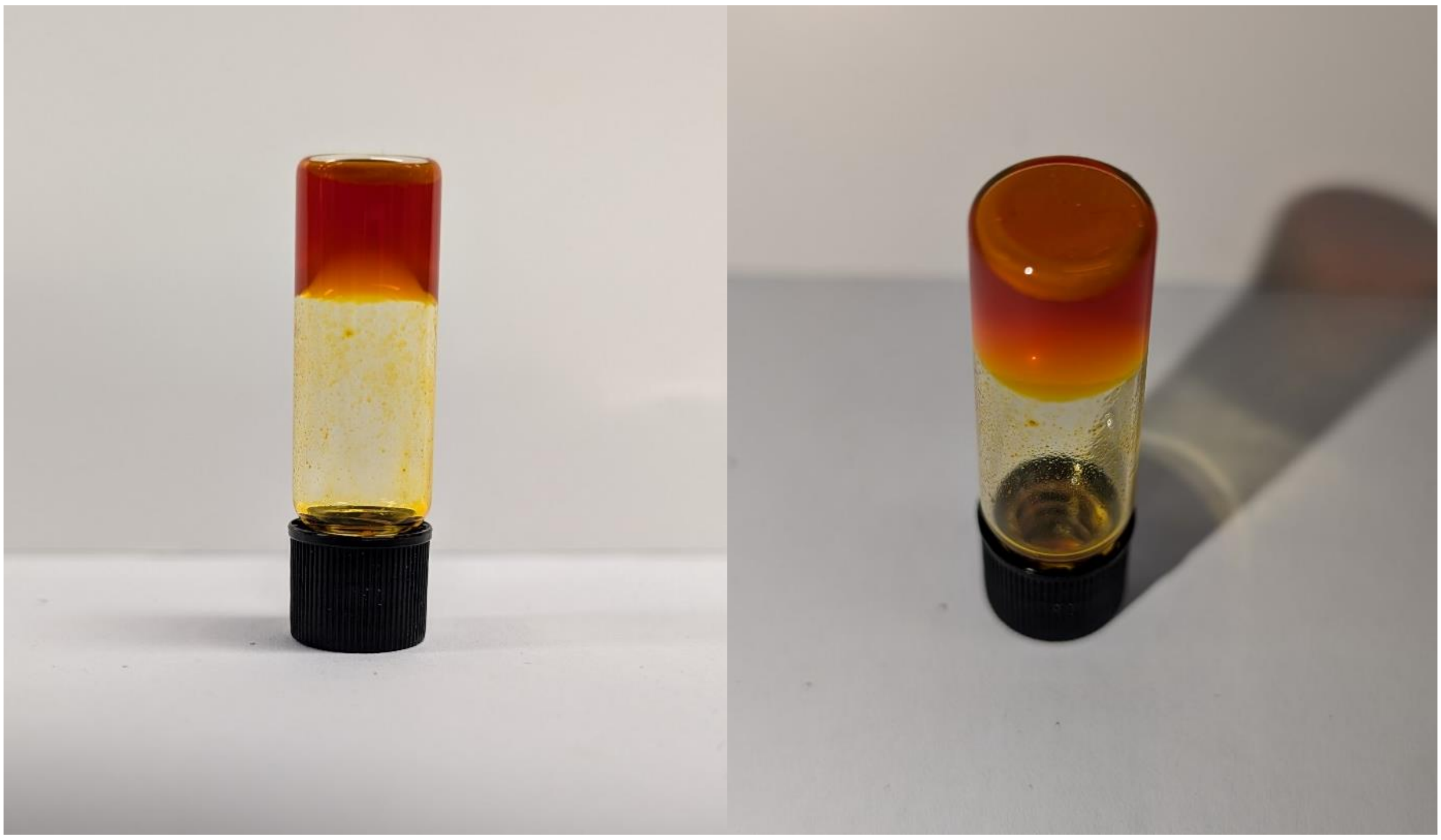

Figure S10: Picture of an metallogel formed in acetonitrile with 2.64 wt.% (g/mL) gelator **1** (1 eq.) and palladium tetrakisacetonitrile tetrafluoroborate (0.5 eq.). Some amount of **1** remains suspended at the bottom of the vial.

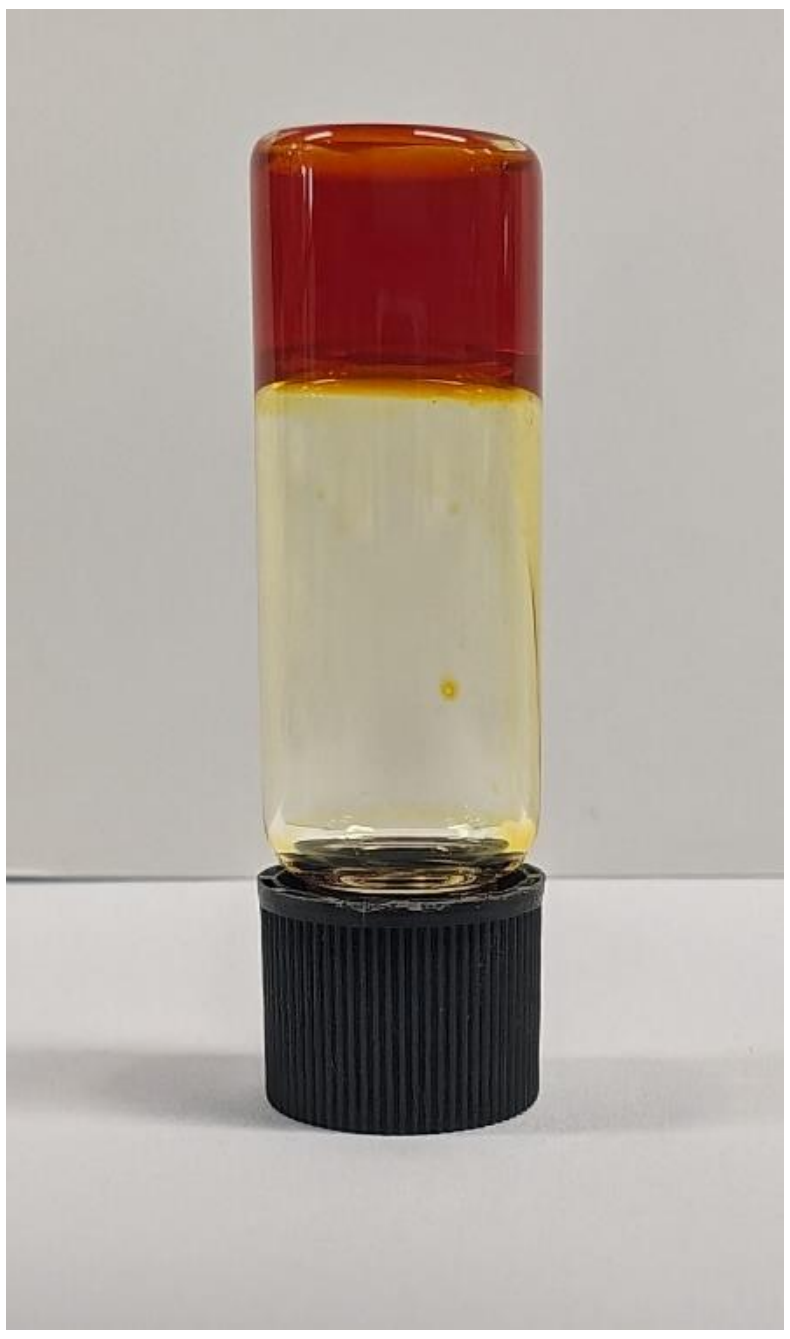

Figure S11: Picture of an metallogel formed in 3:7 v/v acetonitrile:tetrachloroethane with 3 wt.% (g/mL) **1** (1 eq.) and palladium tetrakisacetonitrile tetrafluoroborate (0.5 eq.).

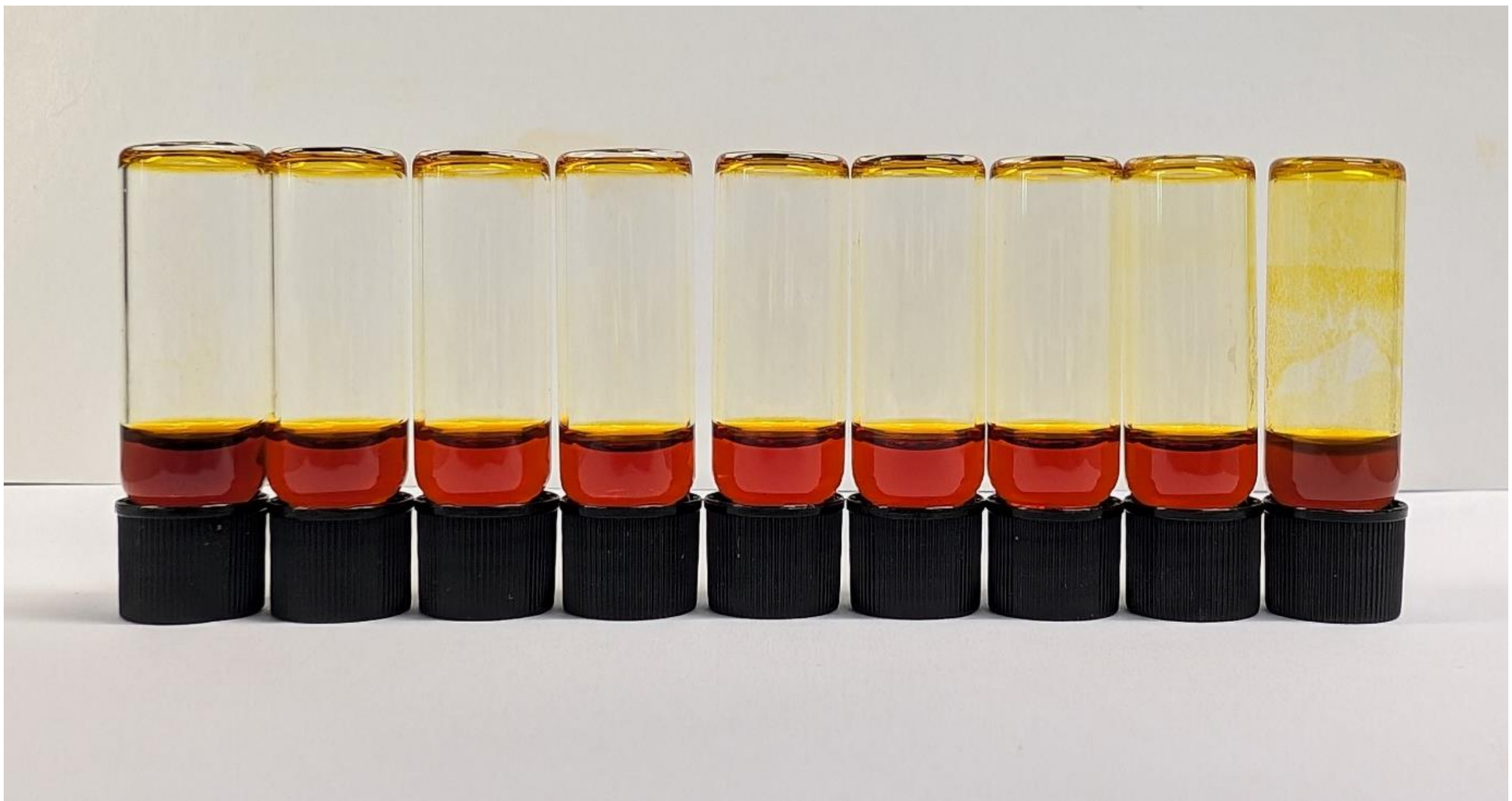

Figure S12: Pictured left to right; 9:1, 8:2, 7:3, 6:4, 1:1, 4:6, 3:7, 2:8, 1:9 acetonitrile: tetrachloroethane mixtures with 1 wt. % (g/mL) **1** (1 eq.) and palladium tetrakisacetonitrile tetrafluoroborate (0.5 eq.).

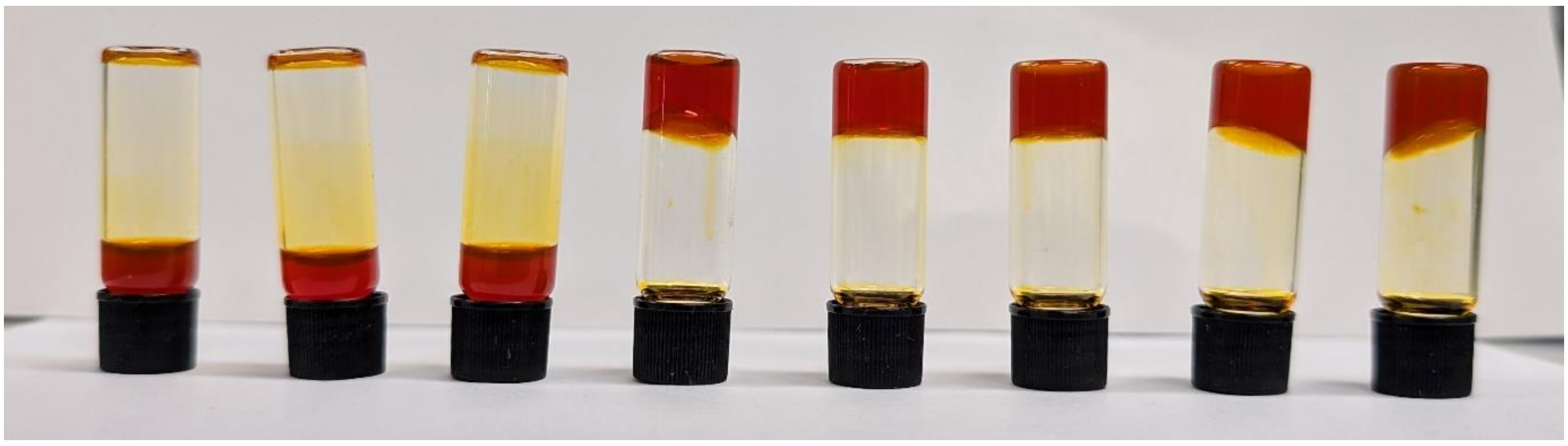

Figure S13: Pictured left to right; 8:2, 7:3, 6:4, 1:1, 4:6, 3:7, 2:8, 1:9 acetonitrile: tetrachloroethane mixtures with 2 wt. % (g/mL) **1** (1 eq.) and palladium tetrakisacetonitrile tetrafluoroborate (0.5 eq.).

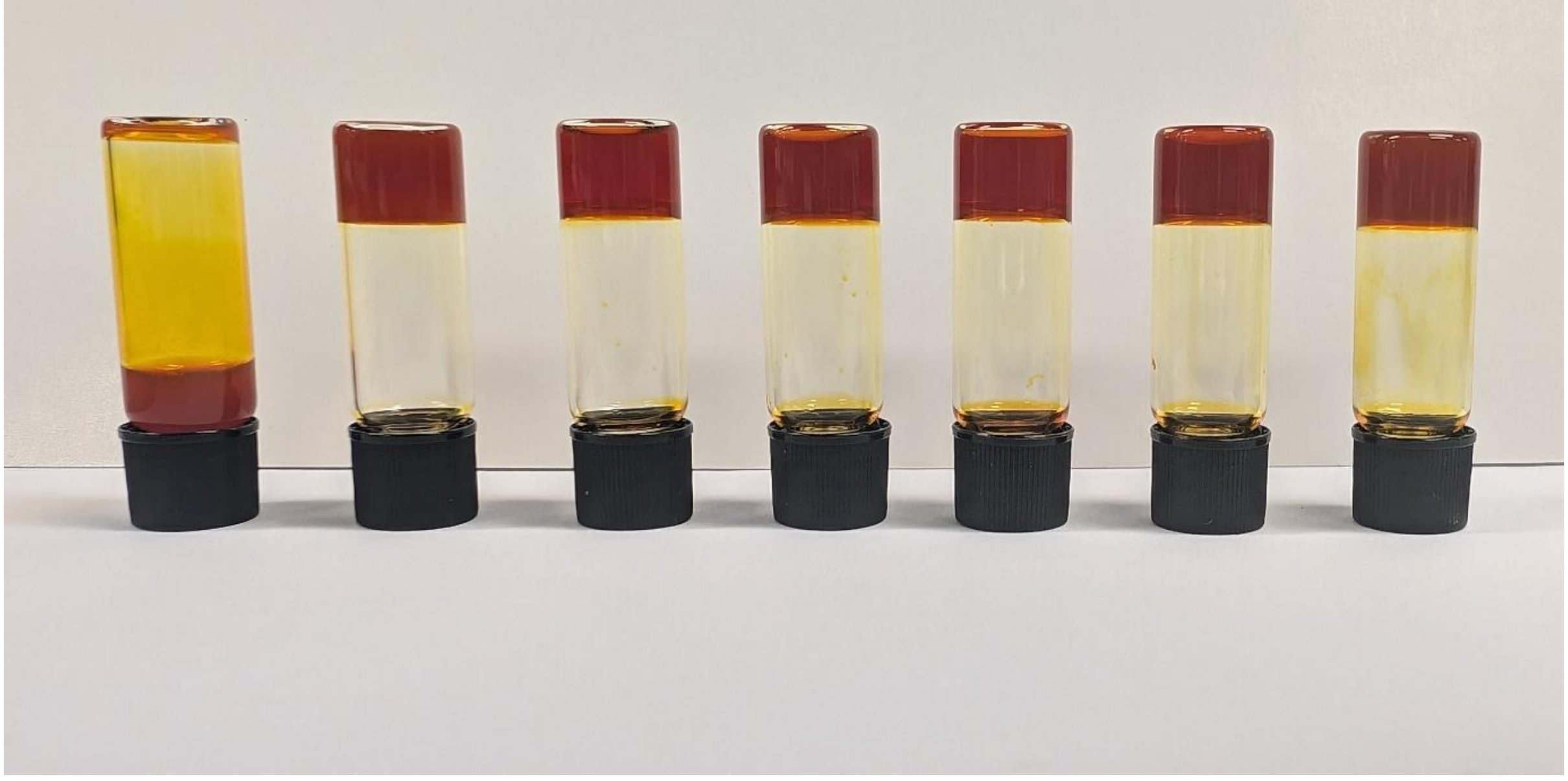

Figure S14: Pictured left to right; 7:3, 6:4, 1:1, 4:6, 3:7, 2:8, 1:9 acetonitrile: tetrachloroethane mixtures with 3 wt. % (g/mL) **1** (1 eq.) and palladium tetrakisacetonitrile tetrafluoroborate (0.5 eq.).

Table S1: Table showing solvent systems tried during metallogel optimization. Hybrid solvent systems are in a 3:7 ratio as written.

Solvent	Gel observed
Tetrachloroethane	No
Dichloromethane	No
Acetonitrile: Tetrachloroethane	Yes
Acetonitrile: Dichloromethane	Yes
Acetonitrile: Chloroform	Yes
Acetonitrile: Dichloroethane	Yes
Nitromethane: Tetrachloroethane	Yes
DMSO: Tetrachloroethane	Yes
DMF: Tetrachloroethane	Yes
Methanol: Tetrachloroethane	Yes
Acetone: Tetrachloroethane	No

Table S2: Table showing solvent systems foldamer: palladium ratios tried during metallogel optimization.

Ratio of 1 to palladium	Gel observed
**1:1**	No
**2:1**	Yes
**3:1**	Yes
**4:1**	No
**5:1**	No
**6:1**	No

Table S3: Table showing temperatures tried during metallogel optimization.

Temperature / °C	Gelation time / min
**30**	30
**40**	15
**50**	10
**60**	8
**70**	5
**80**	2

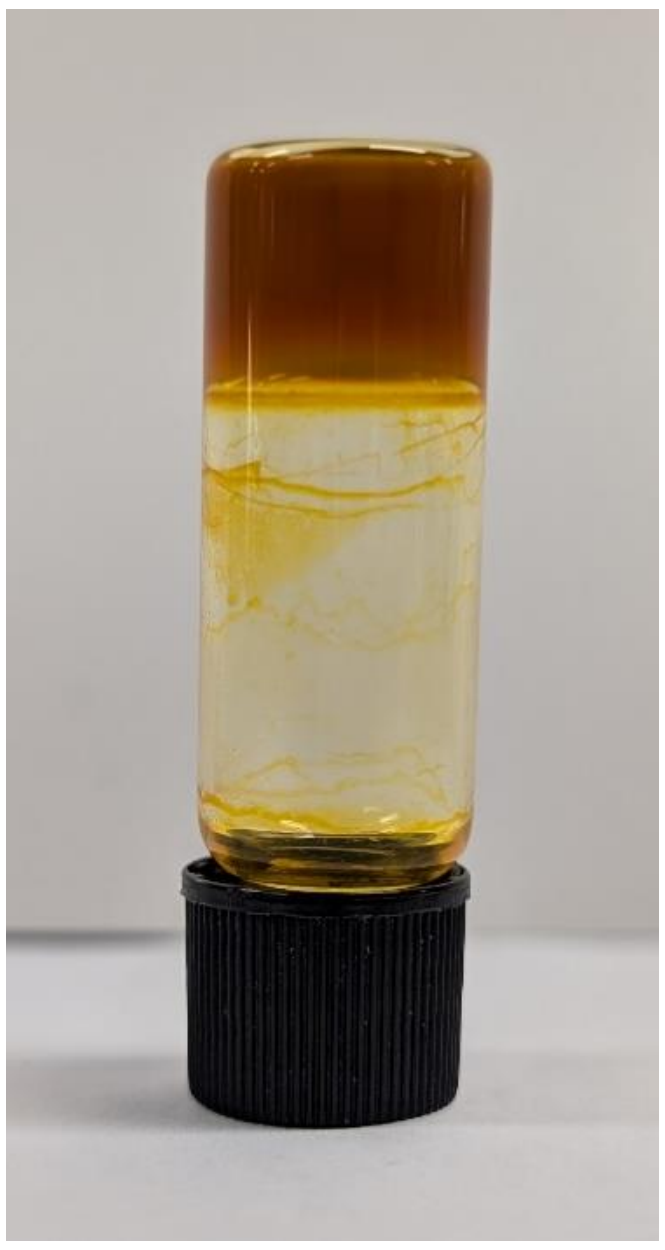

Figure S15: Picture of a metallogel formed in 3:7 acetonitrile: dichloromethane with 3 wt.% (g/mL) **1** (1 eq.) and palladium tetrakisacetonitrile tetrafluoroborate (0.5 eq.).

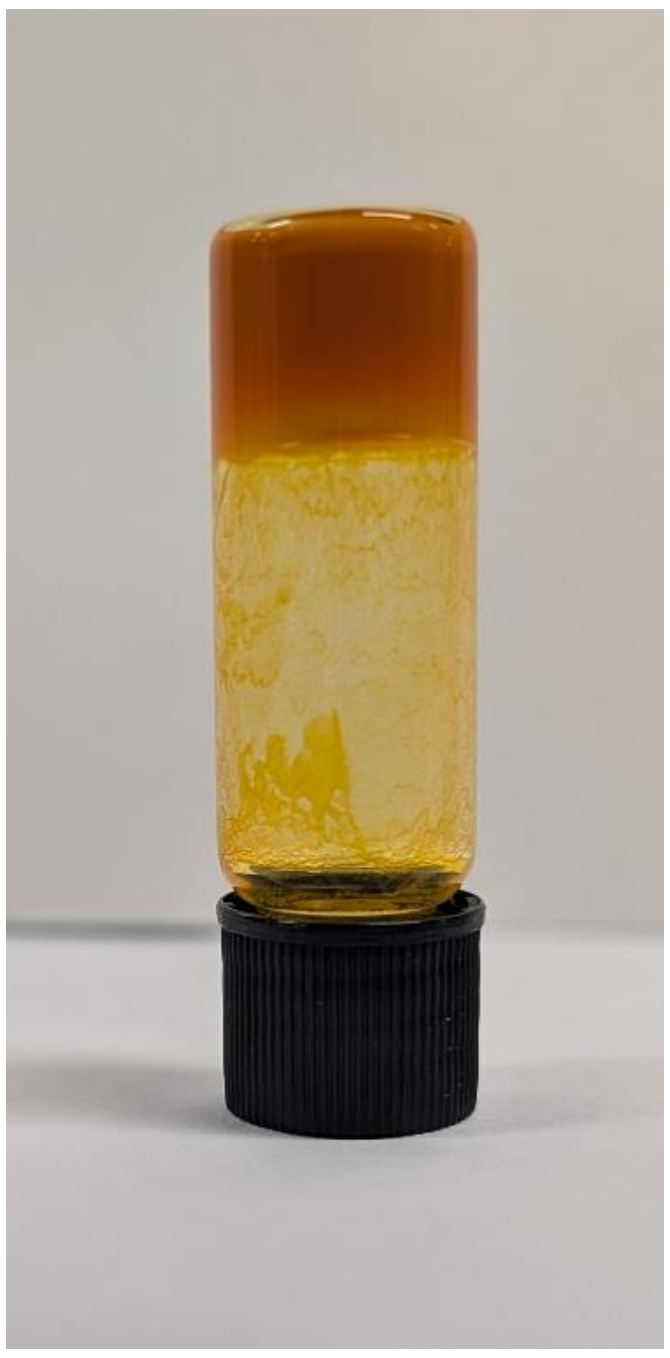

Figure S16: Picture of a metallogel formed in 3:7 acetonitrile: $CHCl_3$ with 3 wt.% (g/mL) **1** (1 eq.) and palladium tetrakisacetonitrile tetrafluoroborate (0.5 eq.).

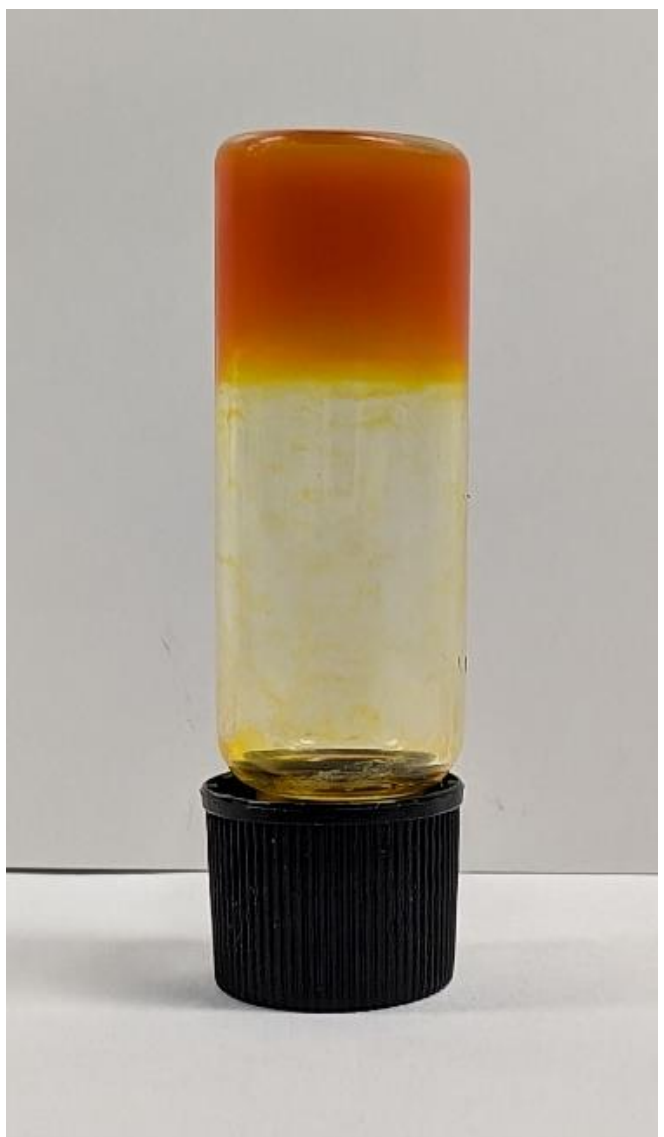

Figure S17: Picture of a metallogel formed in 3:7 acetonitrile: dichloroethane with 3 wt.% (g/mL) **1** (1 eq.) and palladium tetrakisacetonitrile tetrafluoroborate (0.5 eq.).

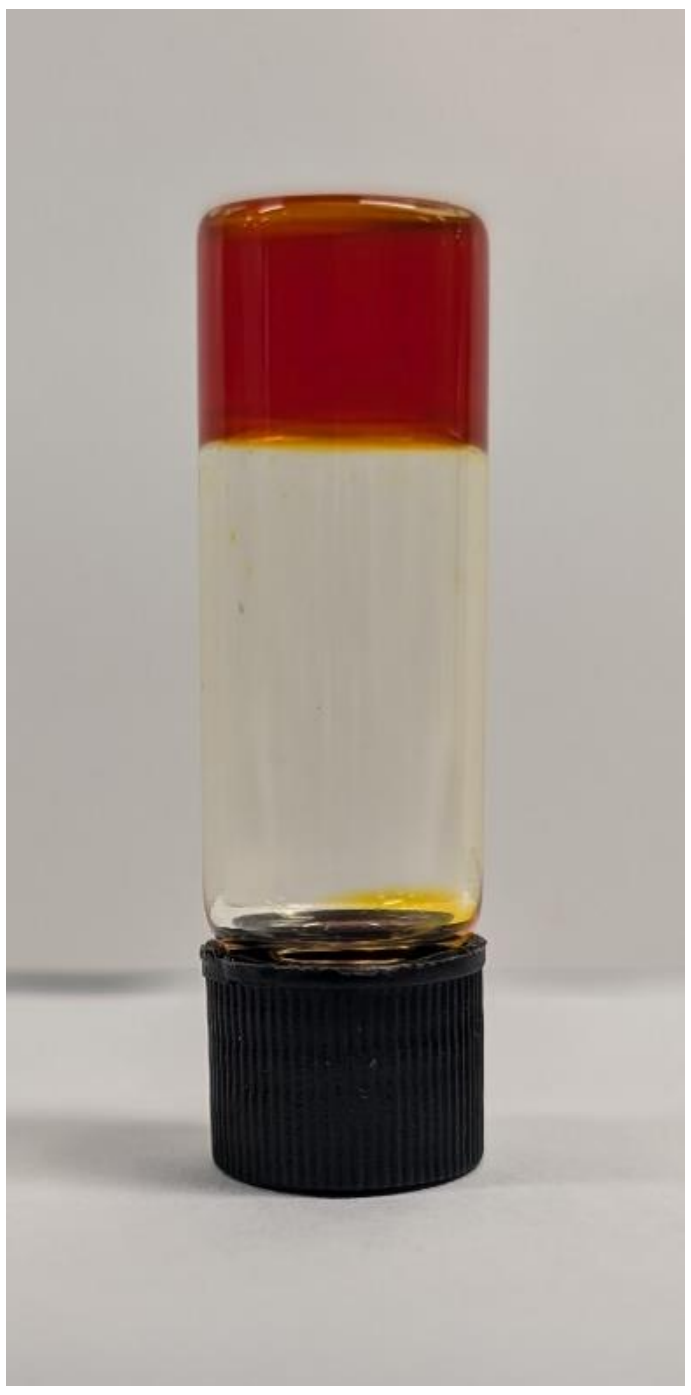

Figure S18: Picture of a metallogel formed in 3:7 nitromethane: tetrachloroethane with 3 wt.% (g/mL) **1** (1 eq.) and palladium tetrakisacetonitrile tetrafluoroborate (0.5 eq.).

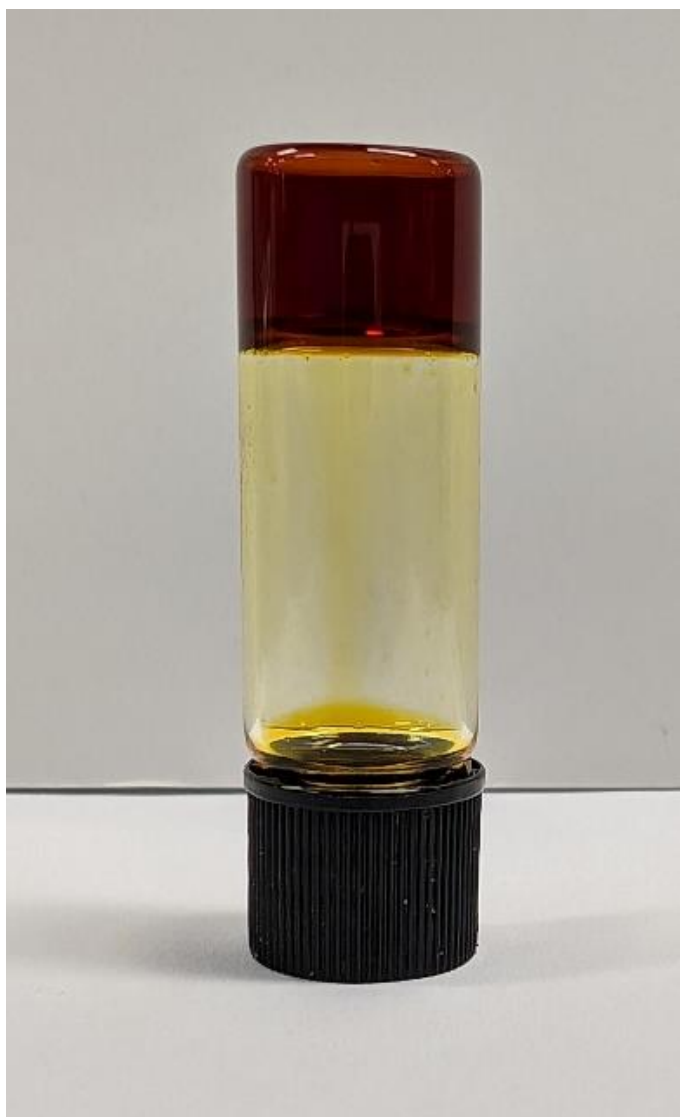

Figure S19: Picture of a metallogel formed in 3:7 DMSO:tetrachloroethane with 3 wt.% (g/mL) **1** (1 eq.) and palladium tetrakisacetonitrile tetrafluoroborate (0.5 eq.).

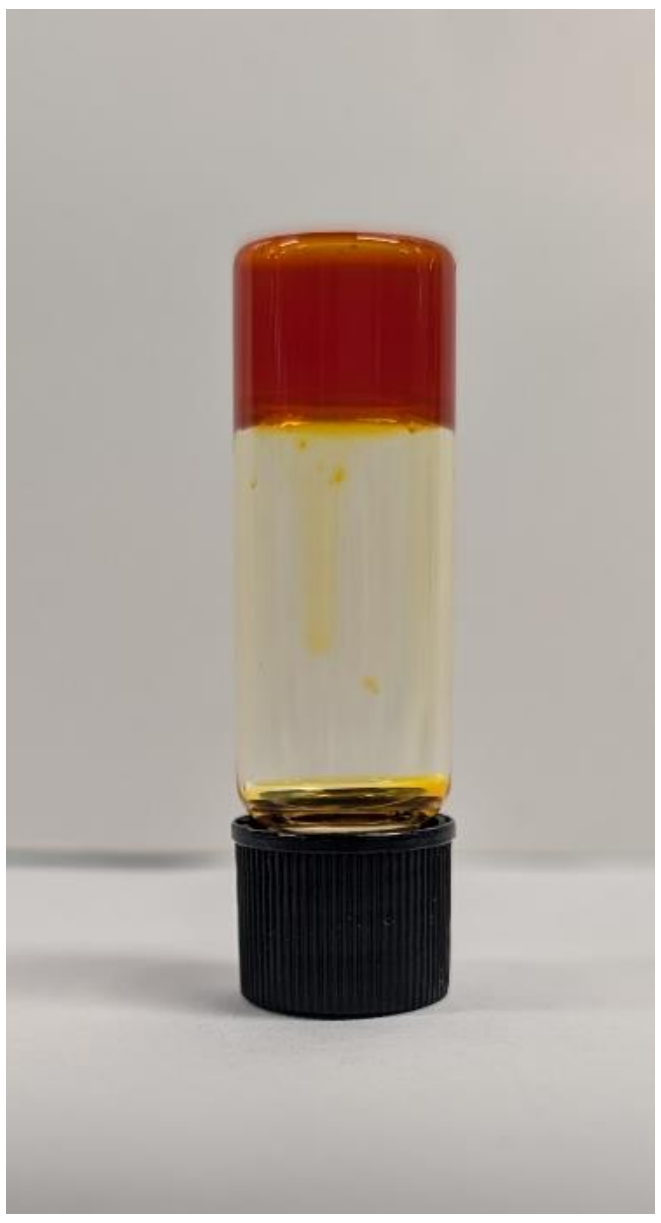

Figure S20: Picture of a metallogel formed in 3:7 DMF:tetrachloroethane with 3 wt.% (g/mL) **1** (1 eq.) and palladium tetrakisacetonitrile tetrafluoroborate (0.5 eq.).

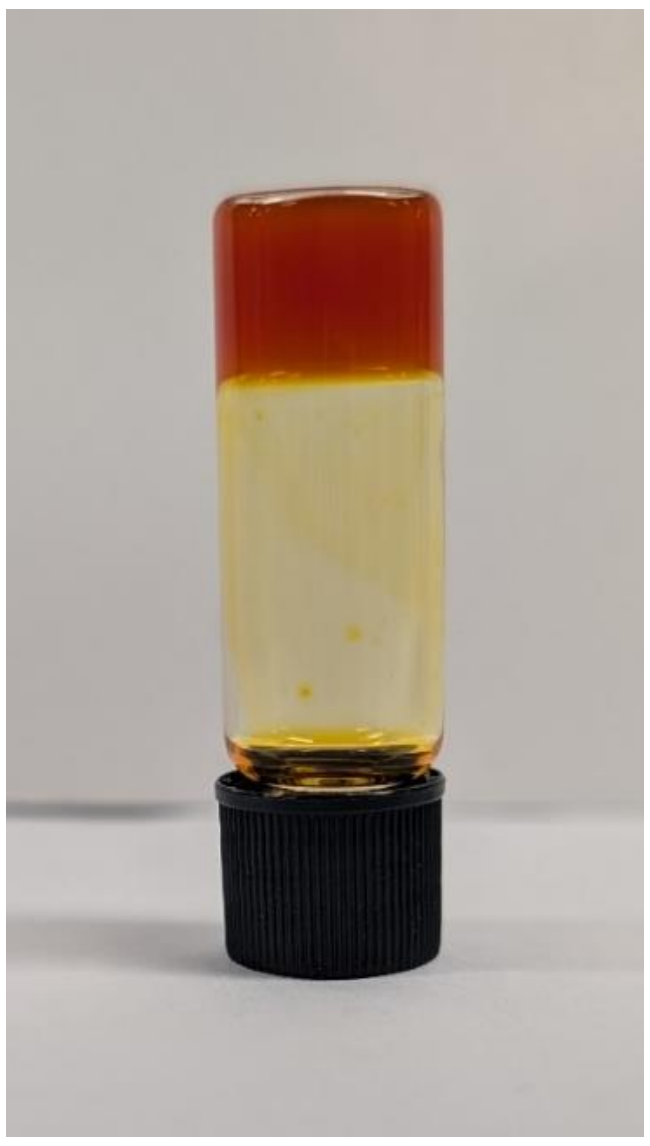

Figure S21: Picture of a metallogel formed in 3:7 methanol:tetrachloroethane with 3 wt.% (g/mL) **1** (1 eq.) and palladium tetrakisacetonitrile tetrafluoroborate (0.5 eq.).

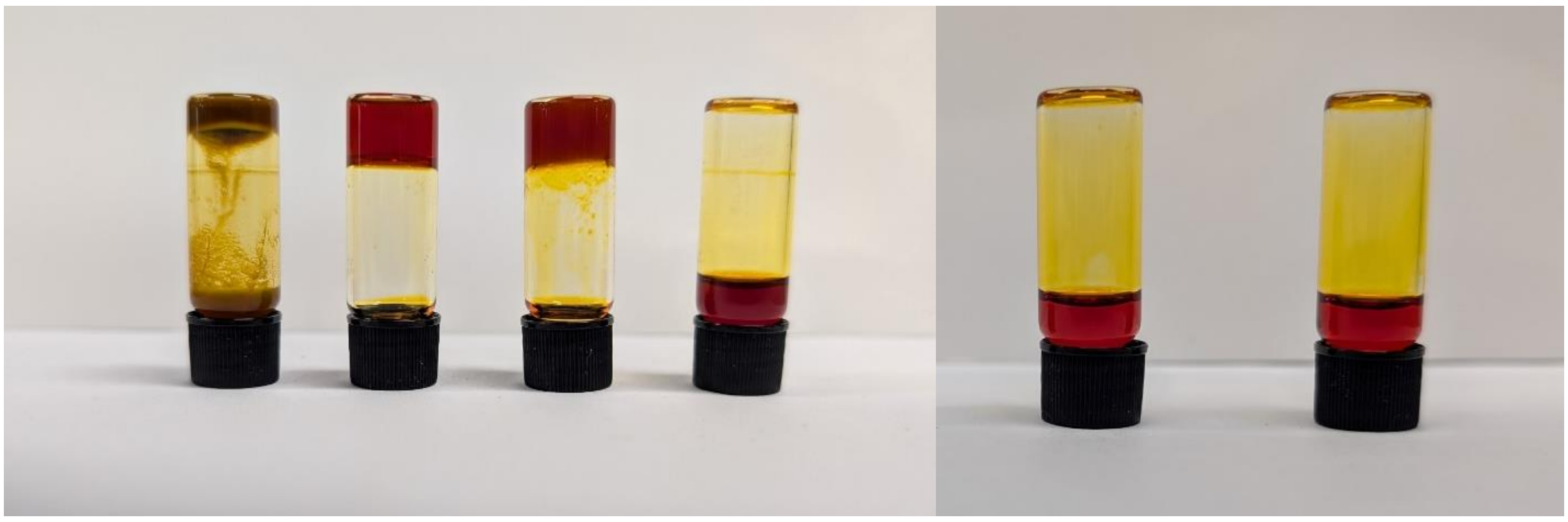

Figure S22: Pictured left to right: metallogel experiments in 3:7 acetonitrile: tetrachloroethane with 3 wt.% (g/mL) **1** (1 eq.) and palladium tetrakisacetonitrile tetrafluoroborate (1 eq.), (0.5 eq.), (0.33 eq.), (0.25 eq.), (0.2 eq.) and (0.17 eq.).

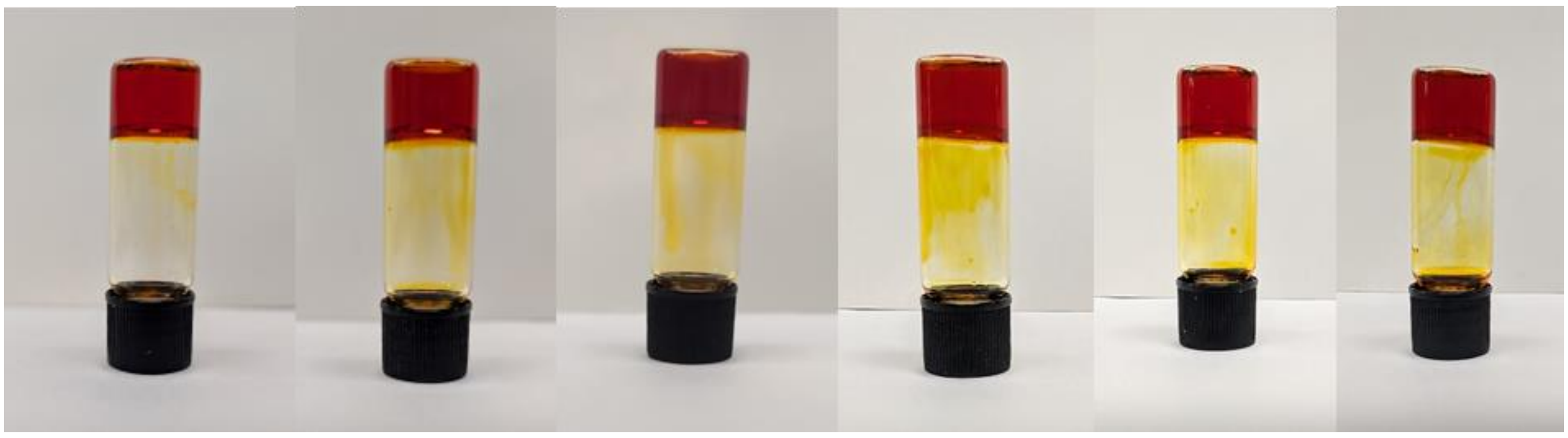

Figure S23: Pictured left to right: metallogel experiments in 3:7 acetonitrile:tetrachloroethane with 3 wt.% (g/mL) **1** (1 eq.) and palladium tetrakisacetonitrile tetrafluoroborate (0.5 eq.), at 30, 40, 50, 60, 70 and 80 °C.

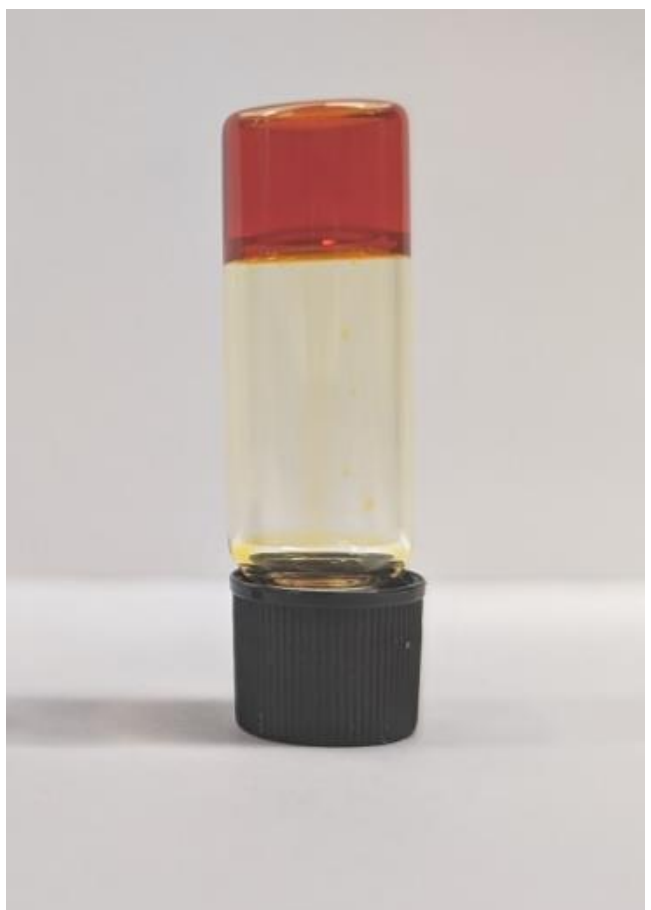

Figure S24: Picture of a metallogel formed in 3:7 acetonitrile:tetrachloroethane with 3 wt.% (g/mL) **1** (1 eq.) and palladium tetrakisacetonitrile triflate (0.5 eq.).

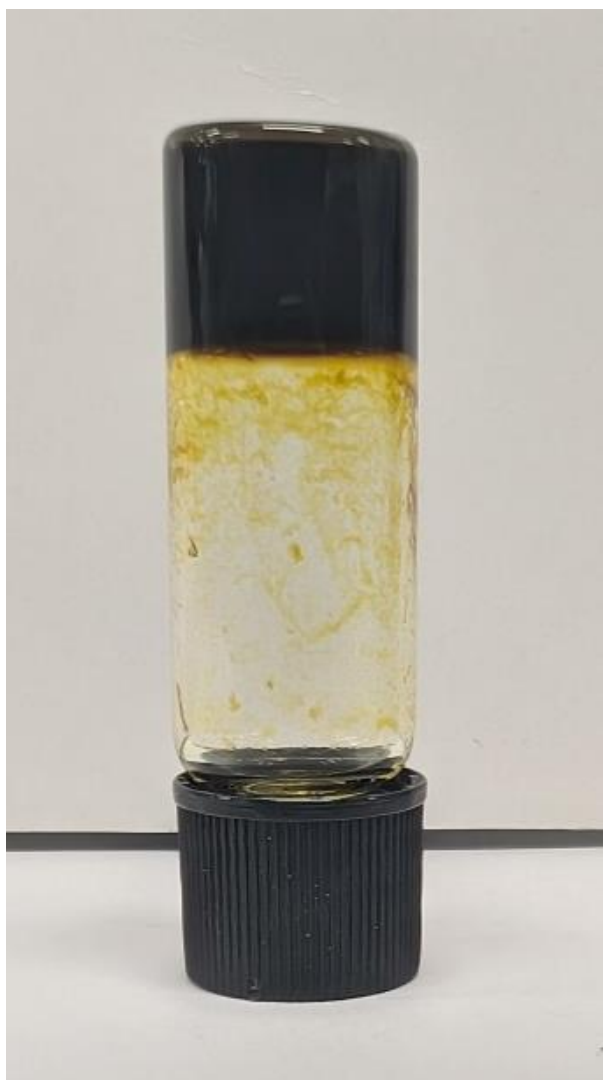

Figure S25: Picture of a metallogel formed in 3:7 DMSO:tetrachloroethane with 3 wt.% (g/mL) **1** (1 eq.) and palladium tetrakisacetonitrile hexafluoroantimonate (0.5 eq.).

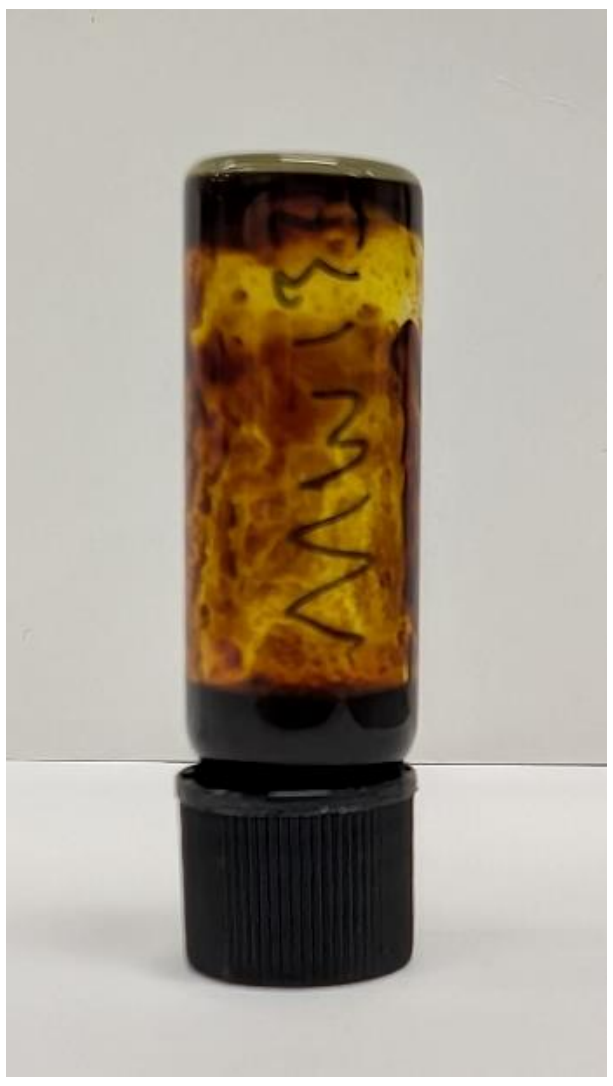

Figure S26: Picture of a metallogel formed in 3:7 DMSO:tetrachloroethane with 3 wt.% (g/mL) **1** (1 eq.) and palladium tetrakisacetonitrile hexafluorophosphate (0.5 eq.).

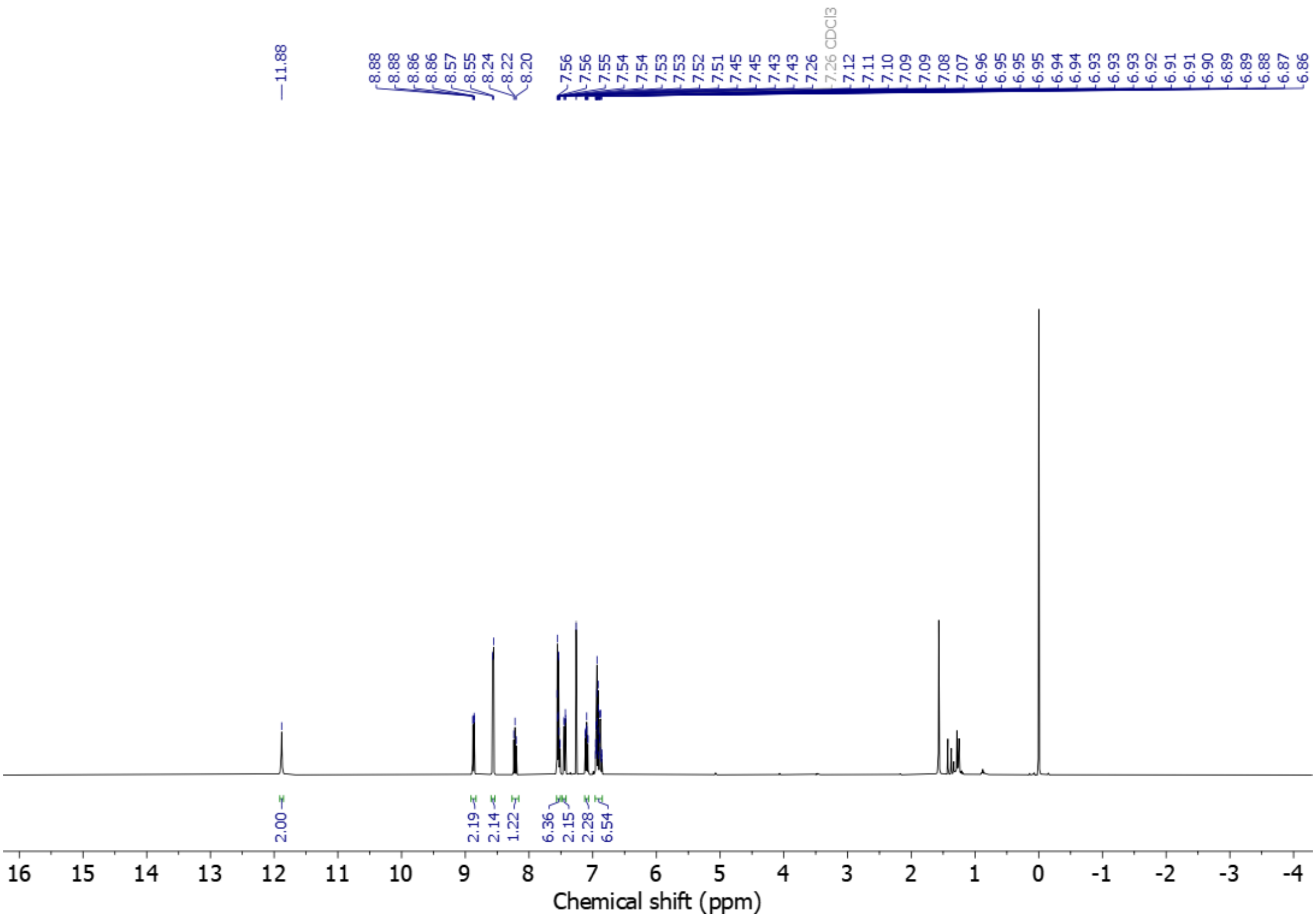

Figure S27: ^{1}H NMR spectrum of **2** (400 MHz, $CDCl_3$, 298 K).

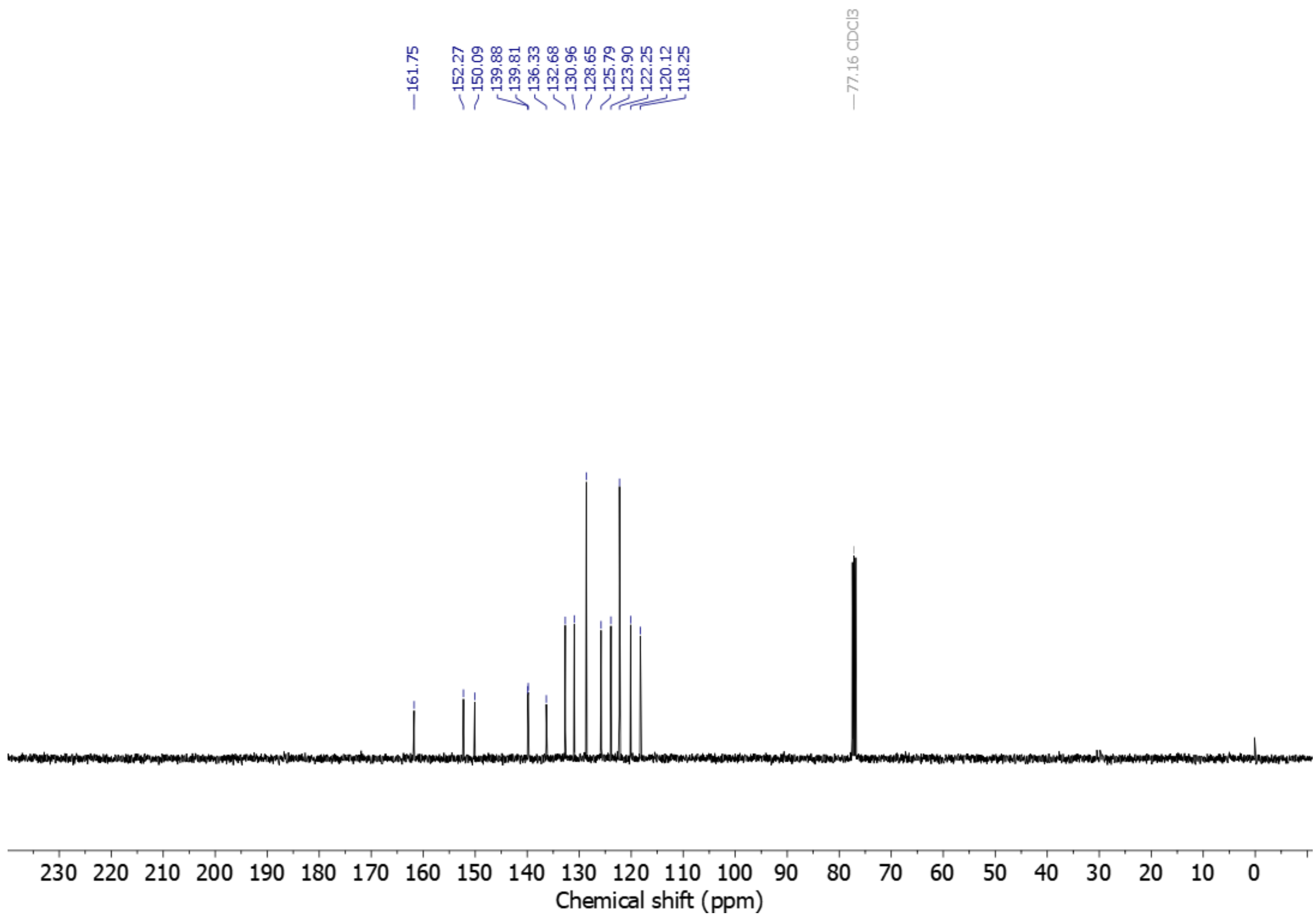

Figure S28: ^{13}C NMR spectrum of **2** (400 MHz, $CDCl_3$, 298 K).

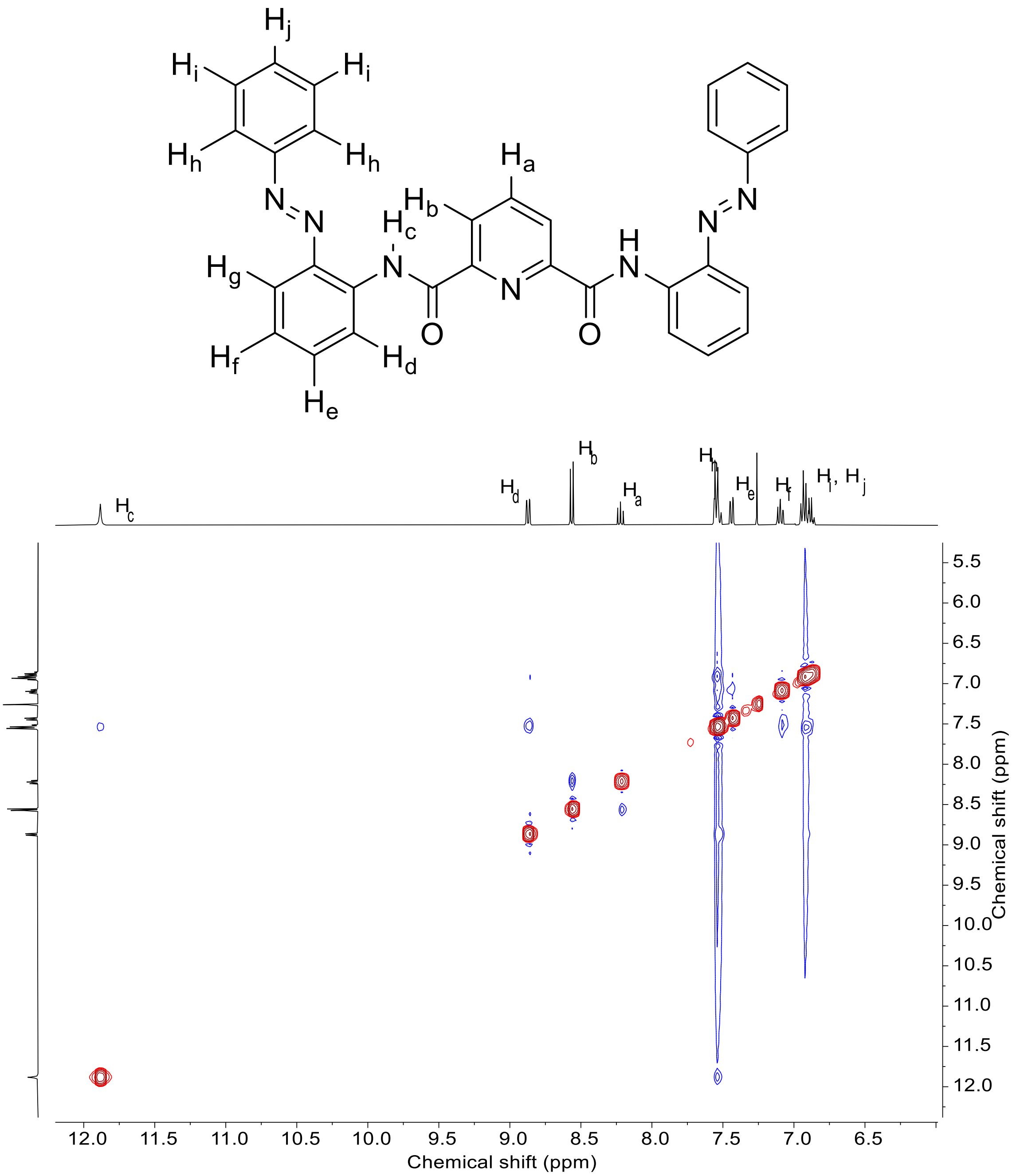

Figure S29: Partial NOESY NMR spectrum of **2** (400 MHz, $CDCl_3$, 298 K). Correlation between protons H_c and H_h indicate the helical *syn*-*syn* conformation.

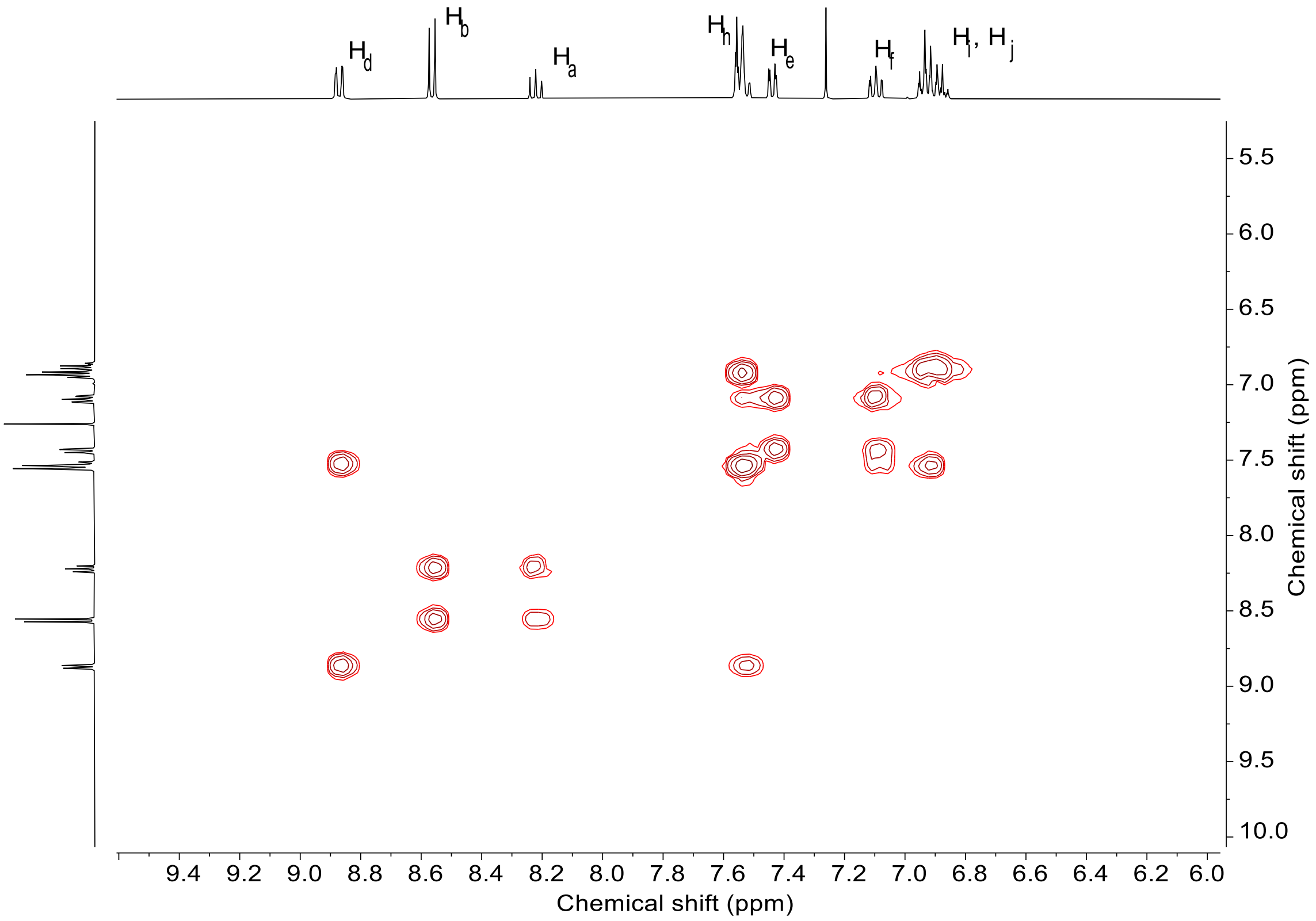

Figure S30: Partial COSY NMR spectrum of **2** (400 MHz, $CDCl_3$, 298 K).

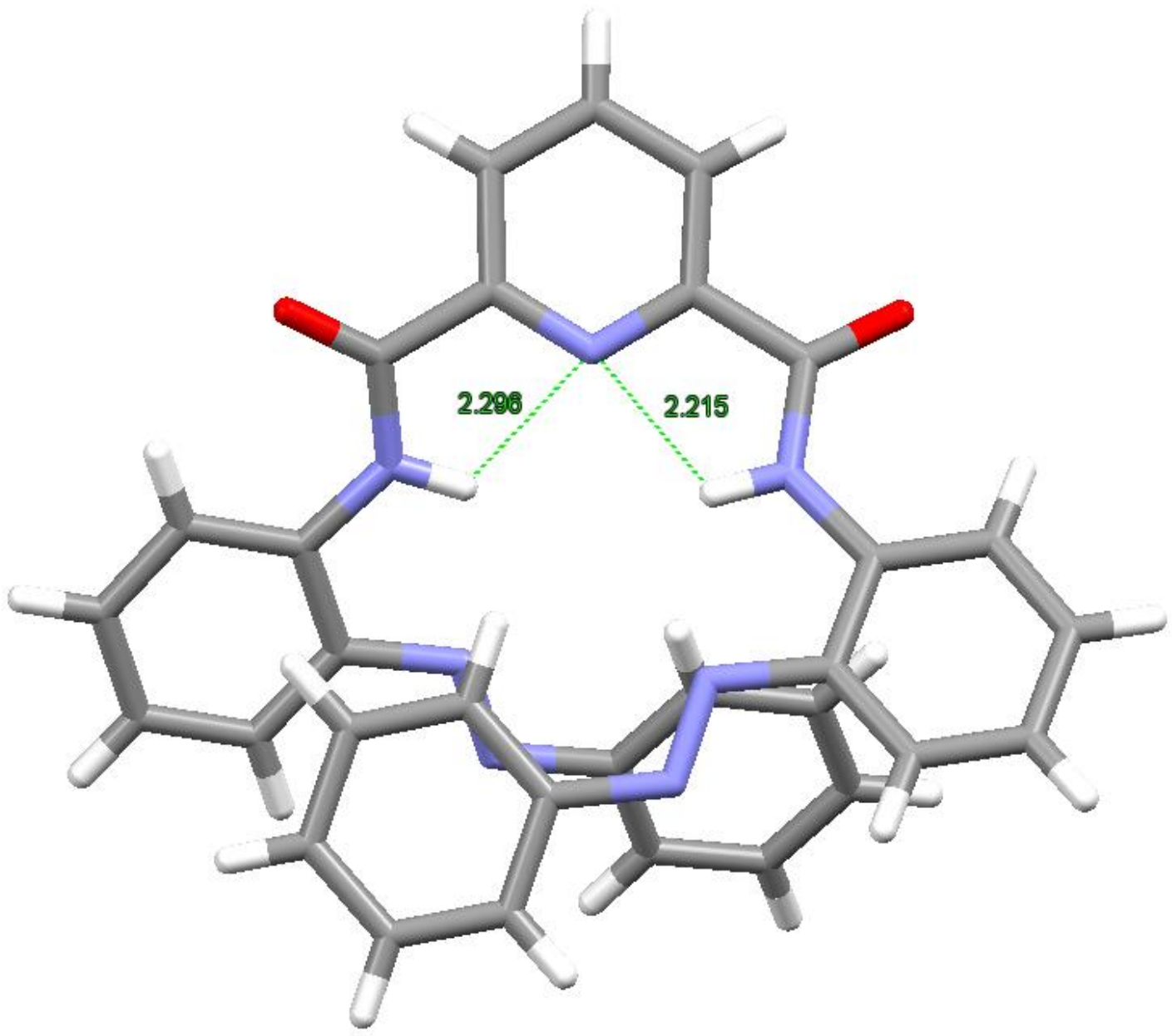

Figure S31: Crystal structure of **2** showing the intramolecular bifurcated hydrogen bonding motif between the N atom of the central pyridine unit and adjacent amide N-H's.

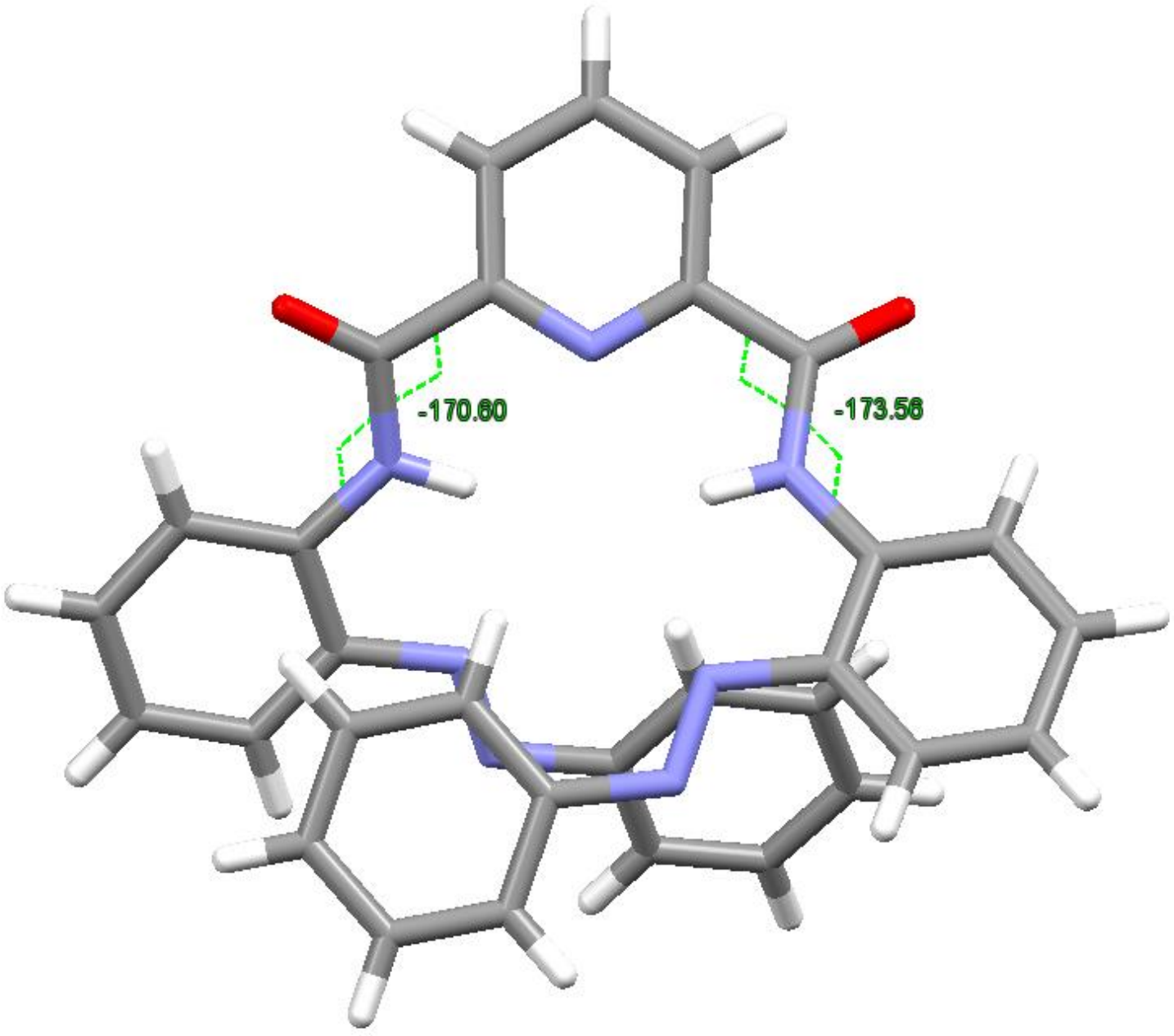

Figure S32: Crystal structure of **2** showing the torsion angles of the internal peptide bonds.

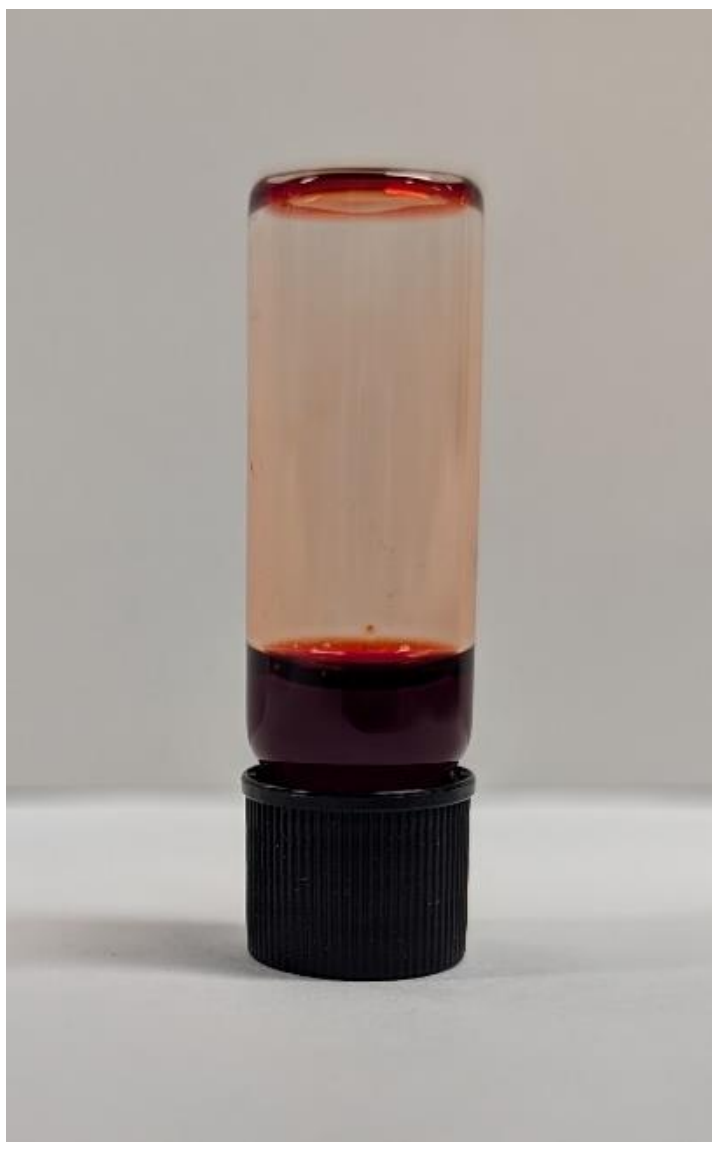

Figure S33: Picture of the resulting solution formed from 3:7 acetonitrile:tetrachloroethane with 3 wt.% (g/mL) negative control, **2** (1 eq.) and palladium tetrakisacetonitrile tetrafluoroborate (0.5 eq.).

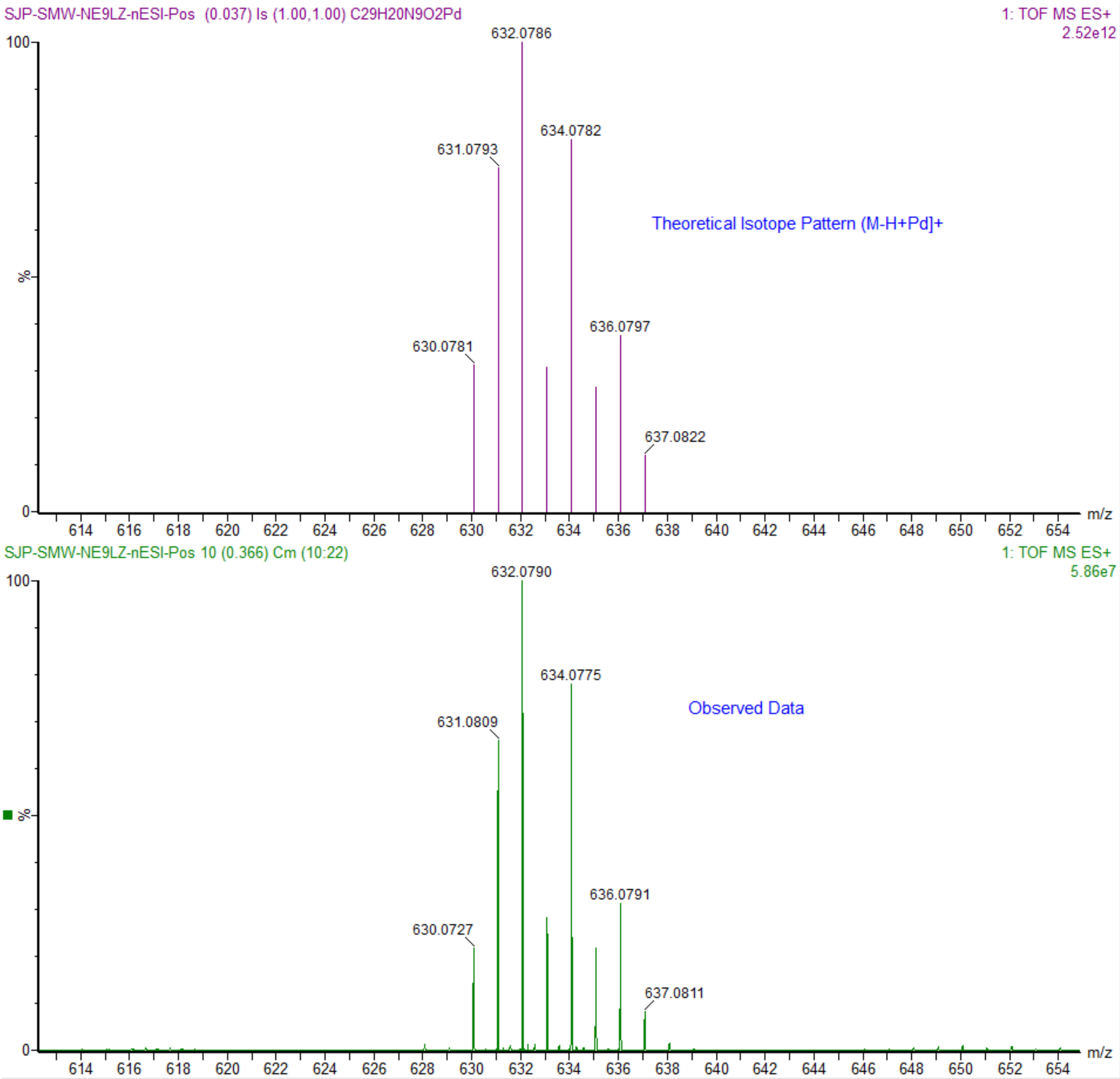

Figure S34: Electrospray ionization mass spectrum of a metallogel sample diluted in acetonitrile. Molecular ion peaks matching the theoretical isotope pattern for a $[M\text{-}H\text{+}Pd]^{+}$ species is observed.

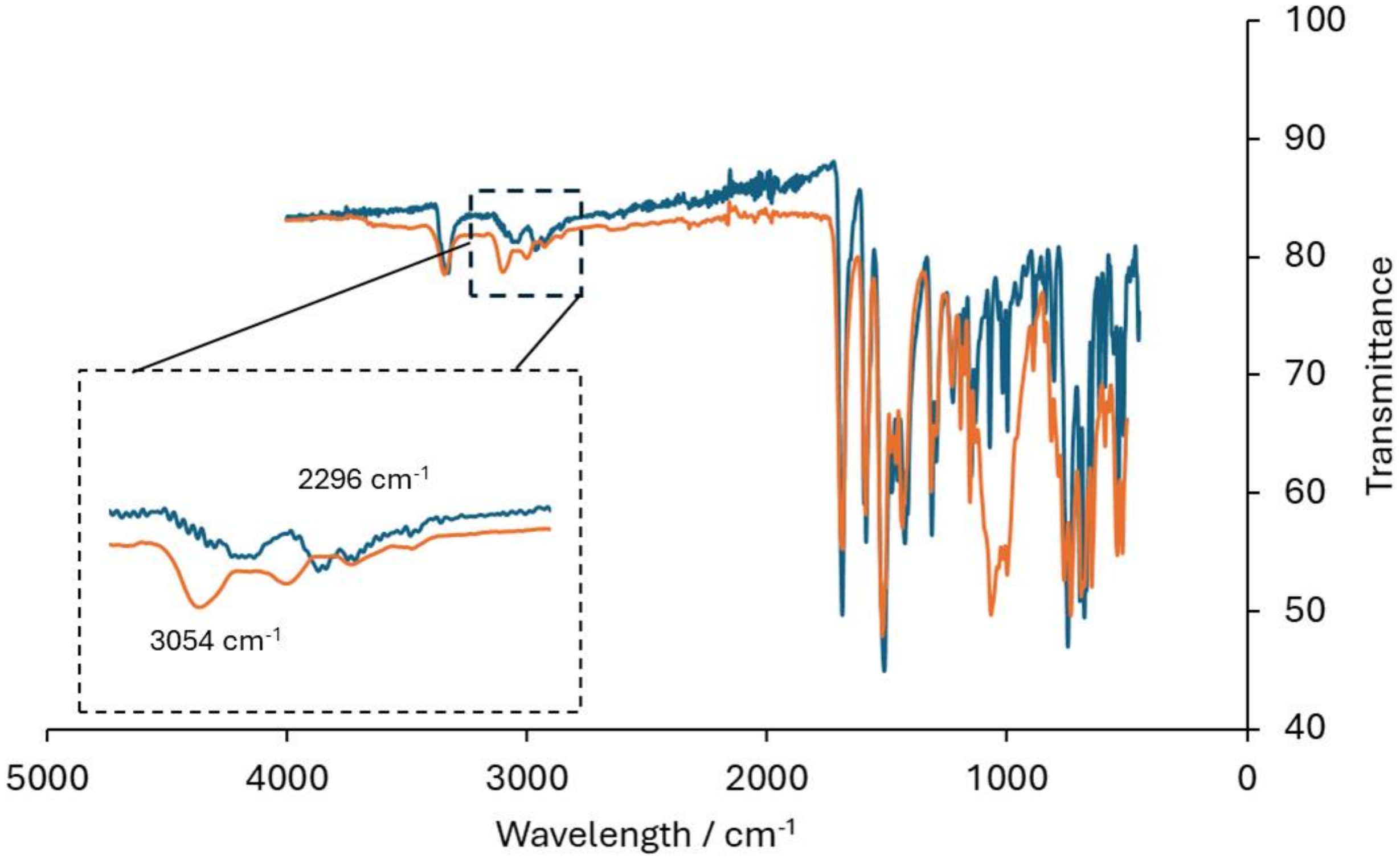

Figure S35: Infrared spectrum comparing foldamer **1** (blue) to the metallogel (orange). A shift in frequencies for aromatic C-H signals is observed.

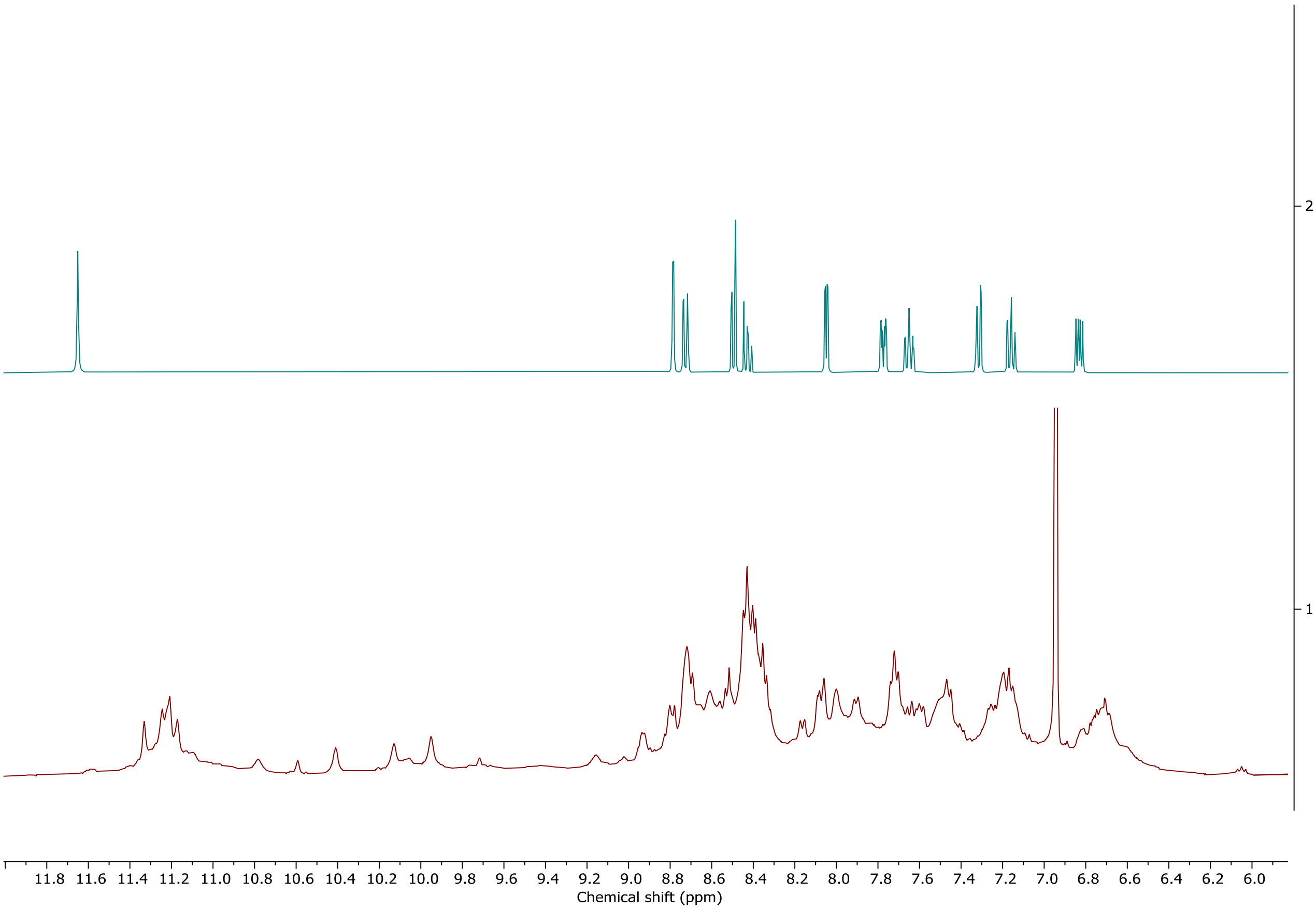

Figure S36: ^{1}H NMR spectra stack of the foldamer (top) and metallogel (bottom) (400 MHz, DMSO-d_6, 298 K).

Table S4: Table summarizing experimental parameters for all solid-state MAS DNP measurements reported in this work. For full experimental details, see Methods. CP = cross-polarization; T_{DNP} = ^{1}H build-up time measured under microwave irradiation; $\varepsilon_{C\ CP}$ = DNP enhancement factor measured via ^{1}H-^{13}C CP on the carbon signals of the foldamer or solvent, as indicated.

**Sample**	**Experiment**	**Micro-waves**	**^{1}H T_{DNP} (s)**	**MAS rate (kHz)**	**Recycle delay (s)**	**Number of scans**	**$\varepsilon_{C\ CP}$** (foldamer)	**$\varepsilon_{C\ CP}$** (solvent)
Foldamer	^{1}H-^{13}C CP	OFF	40.2	10	52.0	24	24	29
	^{1}H-^{13}C CP	ON				32		
	^{1}H-^{15}N CP	ON		8		456		
Organogel	^{1}H-^{13}C CP	OFF	6.5	10	8.5	96	72	66
	^{1}H-^{13}C CP	ON				128		
	^{1}H-^{15}N CP	ON		14		2408		
UV-illuminated organogel	^{1}H-^{13}C CP	OFF	4.4	10	6.0	96	28	29
	^{1}H-^{13}C CP	ON				104		
	^{1}H-^{15}N CP	ON		14		17048		
Thermally healed organogel	^{1}H-^{13}C CP	OFF	3.9	10	5.2	16	23	23
	^{1}H-^{13}C CP	ON				128		
	^{1}H-^{15}N CP	ON		14		26576		
Hydrogel	^{1}H-^{13}C CP	OFF	2.1	10	2.6	200	85	88
	^{1}H-^{13}C CP	ON				128		
	^{1}H-^{15}N CP	ON		14		8144		
Organogel with 16 mM TEKPol in gel pores	^{1}H-^{13}C CP	OFF	1.8	10	2.4	128	15	19
	^{1}H-^{13}C CP	ON				128		

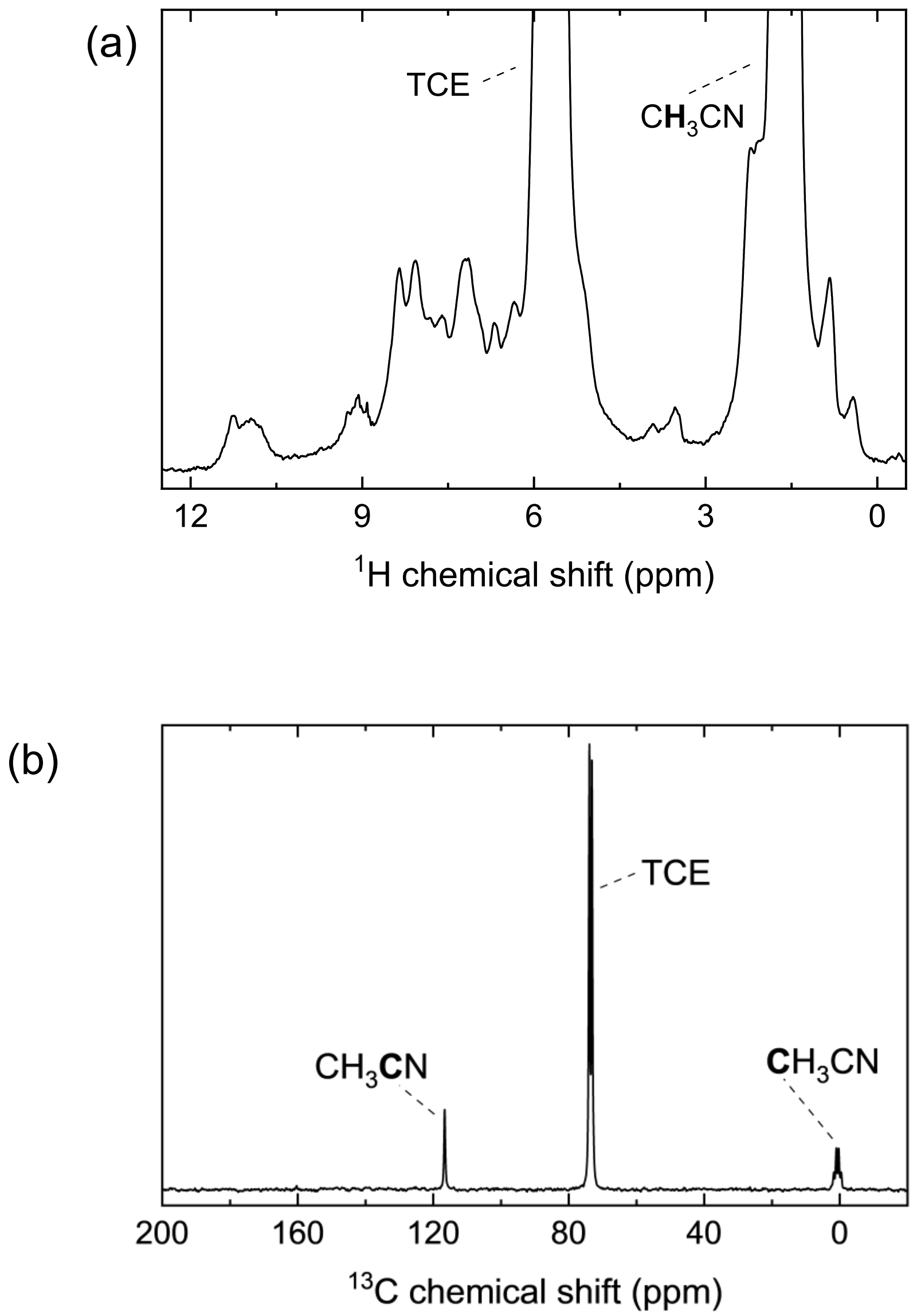

Figure S37: (a) ^{1}H and (b) ^{13}C MAS NMR of the organogel inside a Kel-F insert in a 4 mm ZrO_2 rotor. 9.4 T, 3 kHz MAS, 293 K, direct excitation with echo detection, recycle delay: 5 s, total experiment time: 14 hours.

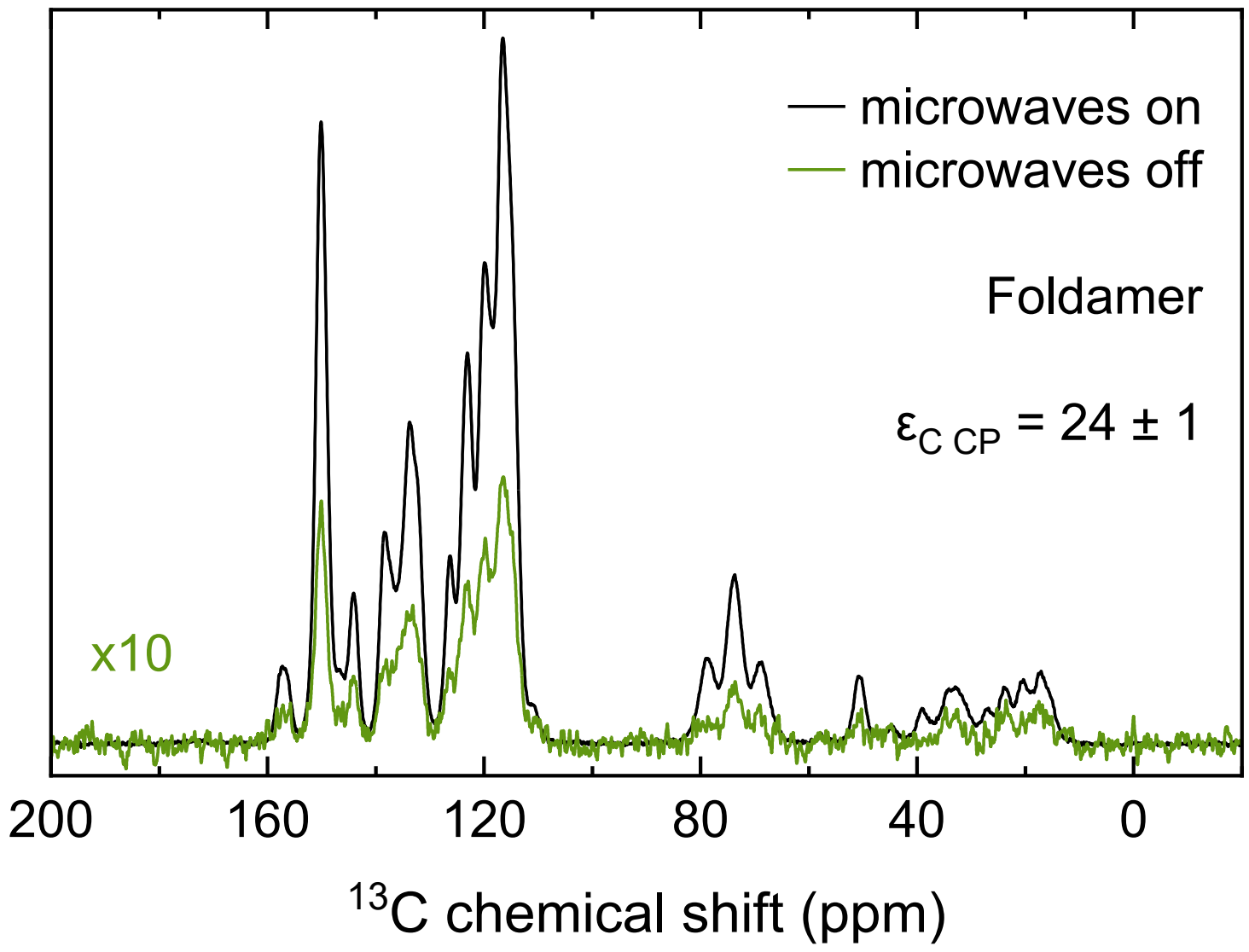

Figure S38: ^{1}H-^{13}C CP MAS DNP NMR spectra of the pristine foldamer with and without DNP (i.e. with and without microwave irradiation). The DNP enhancement factor, $\varepsilon_{C\ CP}$, is calculated by taking the ratio of the most intense signal corresponding to the foldamer with and without DNP, adjusting for the number of scans accumulated for each spectrum (**Table S1**). ^{1}H decoupling is applied during these measurements.

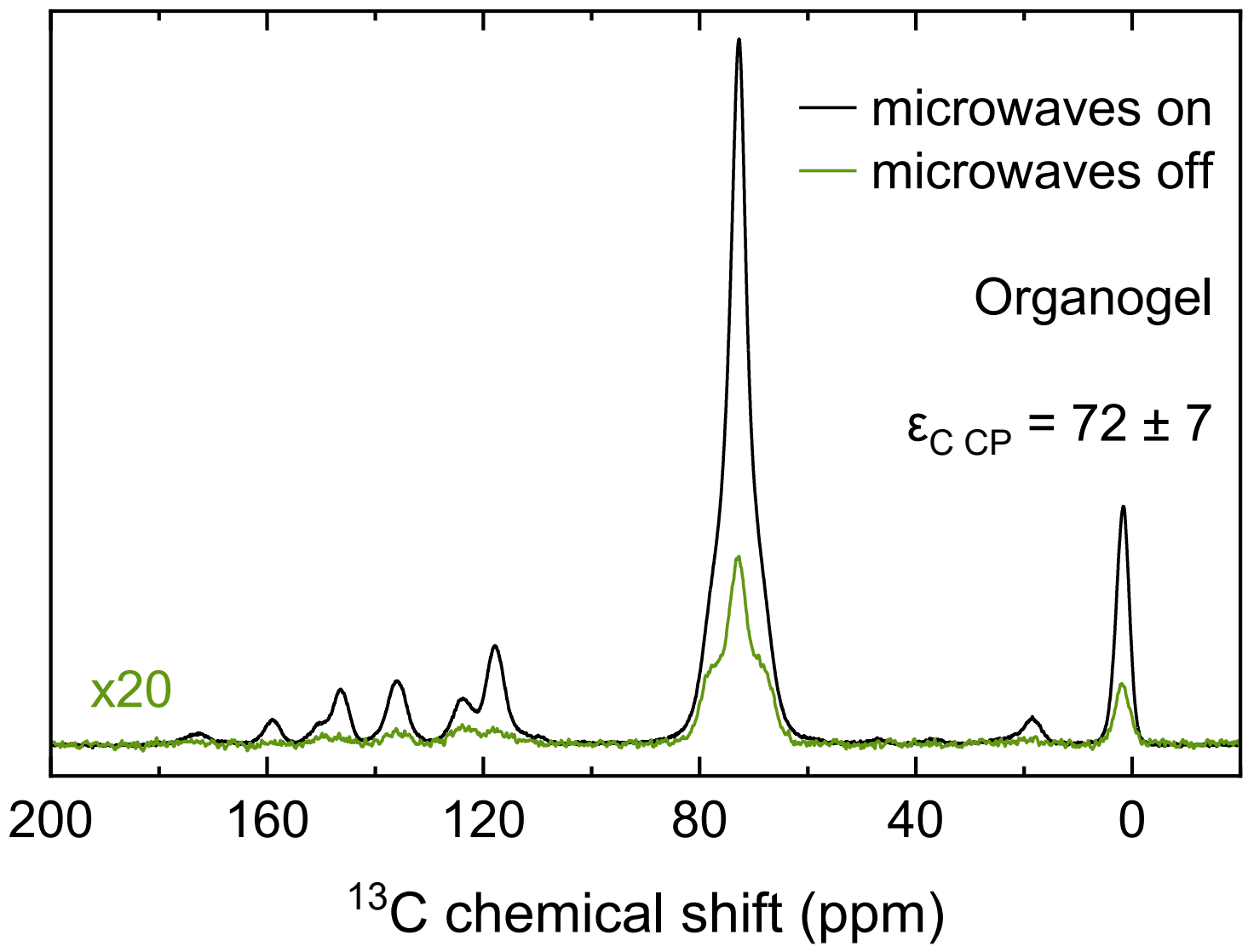

Figure S39: ^{1}H-^{13}C CP MAS DNP NMR spectra of the pristine metallogel with and without DNP (i.e. with and without microwave irradiation). The DNP enhancement factor, $\varepsilon_{C\ CP}$, is calculated by taking the ratio of the most intense signal corresponding to the foldamer with and without DNP, adjusting for the number of scans accumulated for each spectrum (**Table S1**). ^{1}H decoupling is applied during these measurements.

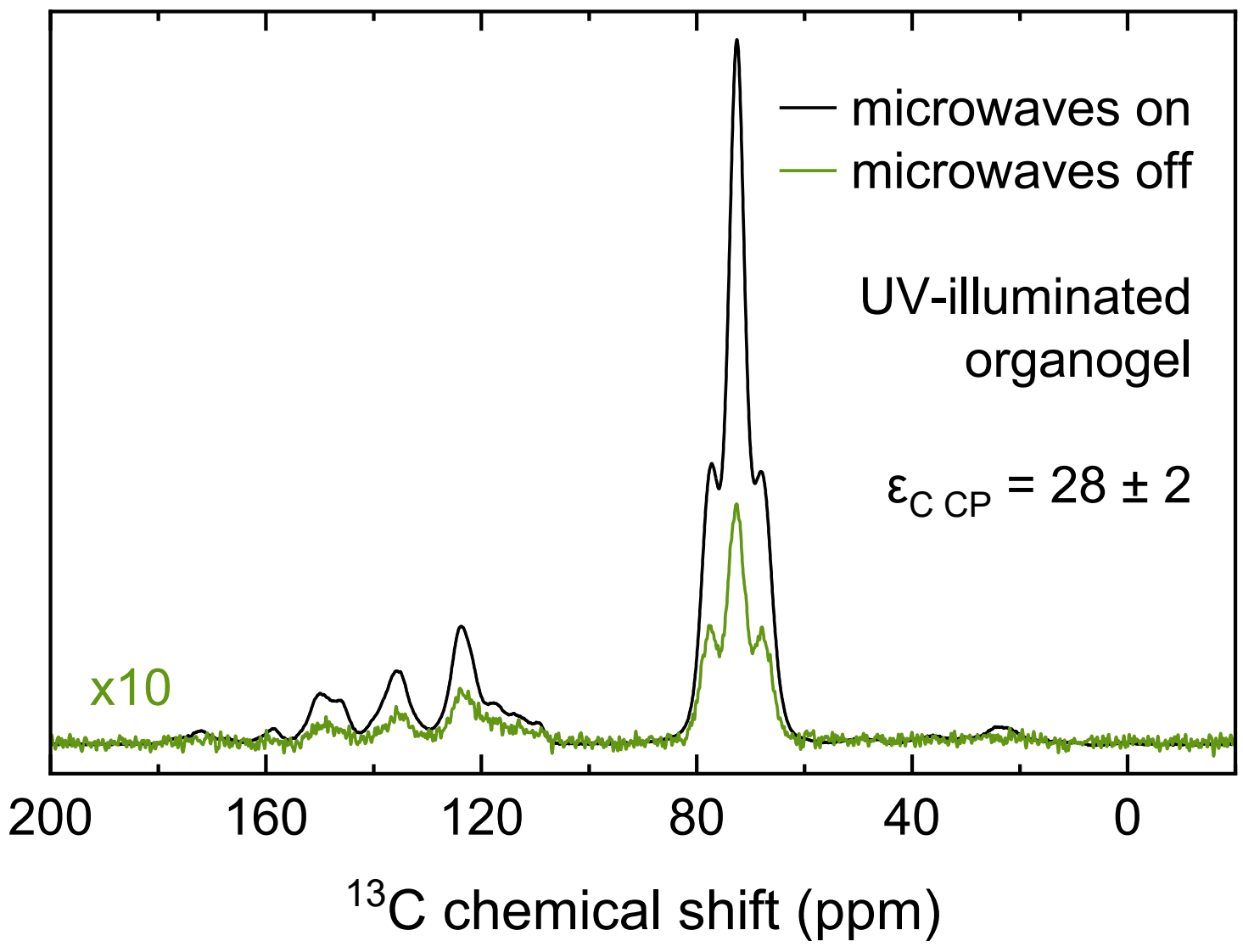

Figure S40: ^{1}H-^{13}C CP MAS DNP NMR spectra of the UV-illuminated metallogel (sol) with and without DNP (i.e. with and without microwave irradiation). The DNP enhancement factor, $\varepsilon_{C\ CP}$, is calculated by taking the ratio of the most intense signal corresponding to the foldamer with and without DNP, adjusting for the number of scans accumulated for each spectrum (**Table S1**). ^{1}H decoupling is applied during these measurements.

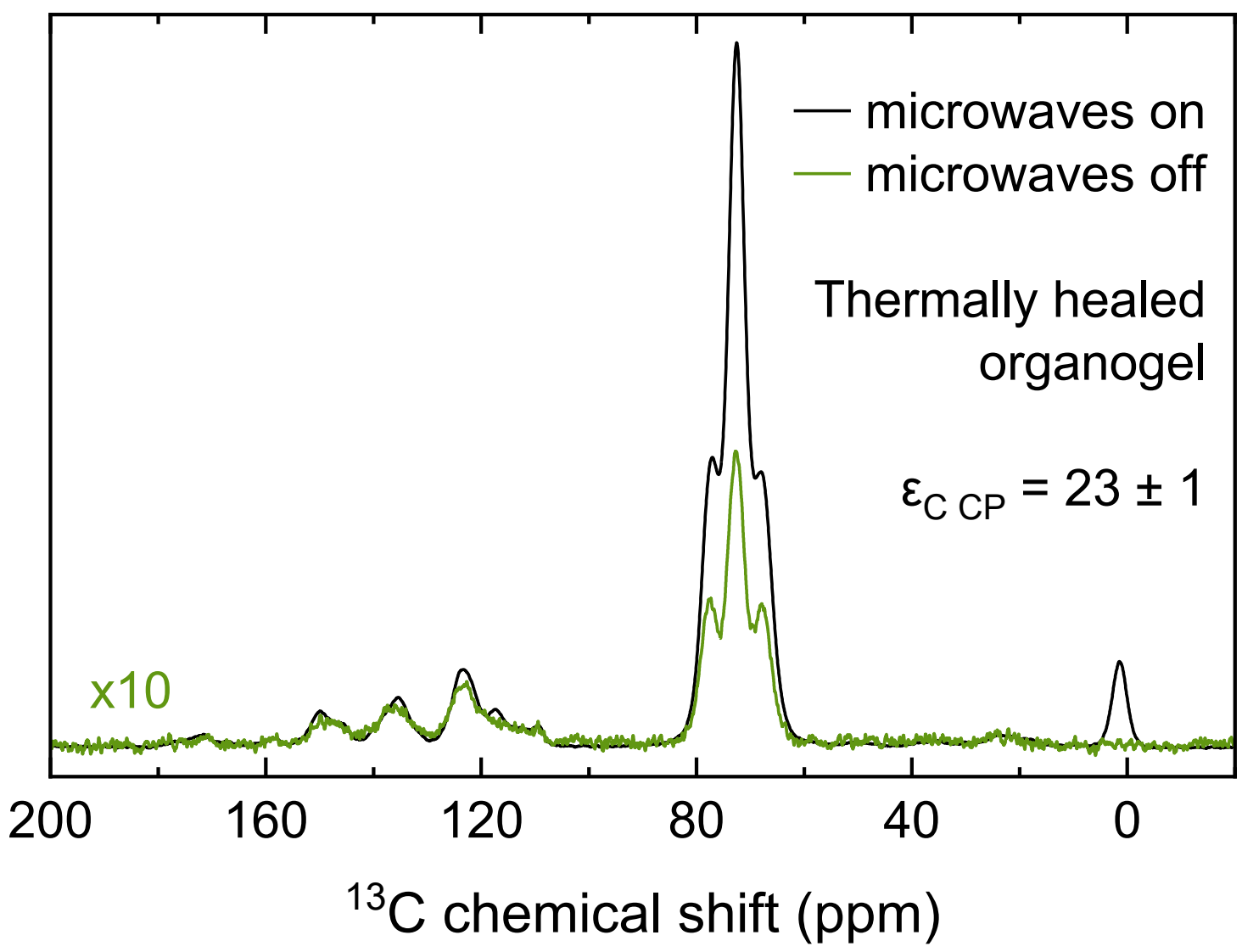

Figure S41: ^{1}H-^{13}C CP MAS DNP NMR spectra of the thermally healed metallogel with and without DNP (i.e. with and without microwave irradiation). The DNP enhancement factor, $\varepsilon_{C\ CP}$, is calculated by taking the ratio of the most intense signal corresponding to the foldamer with and without DNP, adjusting for the number of scans accumulated for each spectrum (**Table S1**). ^{1}H decoupling is applied during these measurements.

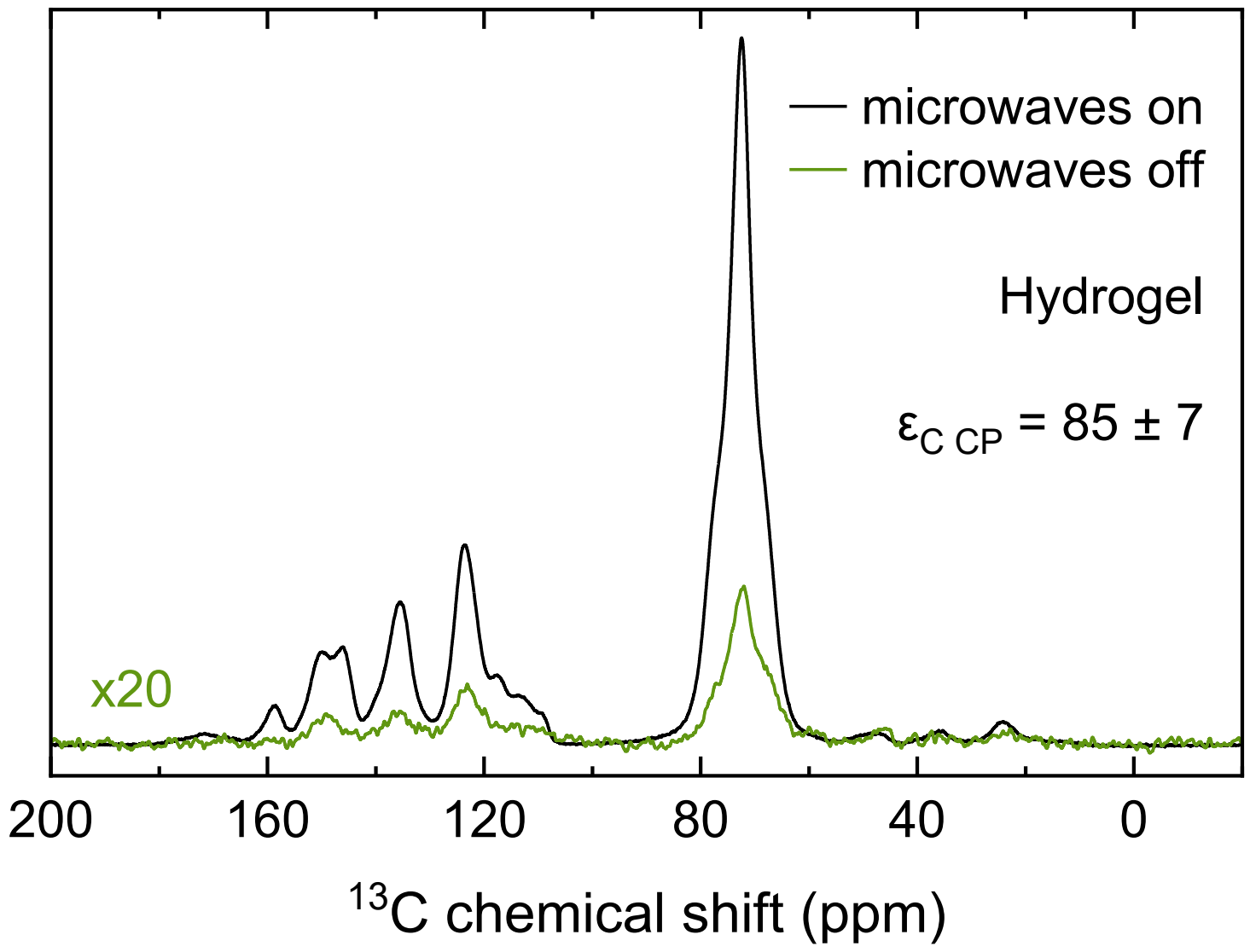

Figure S42: ^{1}H-^{13}C CP MAS DNP NMR spectra of the hydrogel with and without DNP (i.e. with and without microwave irradiation). The DNP enhancement factor, $\varepsilon_{C\ CP}$, is calculated by taking the ratio of the most intense signal corresponding to the foldamer with and without DNP, adjusting for the number of scans accumulated for each spectrum (**Table S1**). ^{1}H decoupling is applied during these measurements.

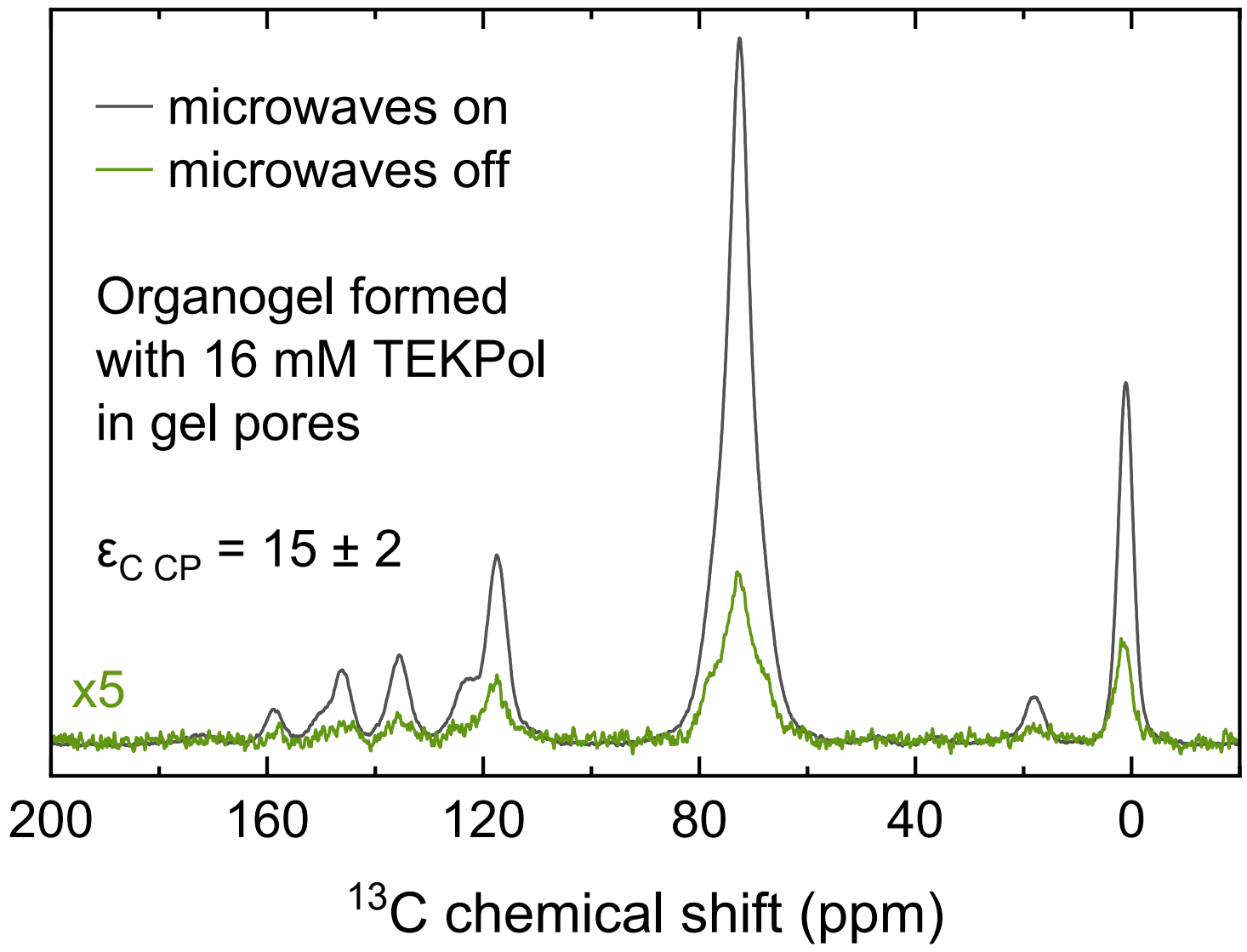

Figure S43: ^{1}H-^{13}C CP MAS DNP NMR spectra of the pristine metallogel formed with 16 mM TEKPol dissolved in the gel pores (containing 7:3 v/v TCE:MeCN) with and without DNP (i.e. with and without microwave irradiation). The DNP enhancement factor, $\varepsilon_{C\ CP}$, is calculated by taking the ratio of the most intense signal corresponding to the foldamer with and without DNP, adjusting for the number of scans accumulated for each spectrum (**Table S1**). ^{1}H decoupling is applied during these measurements.

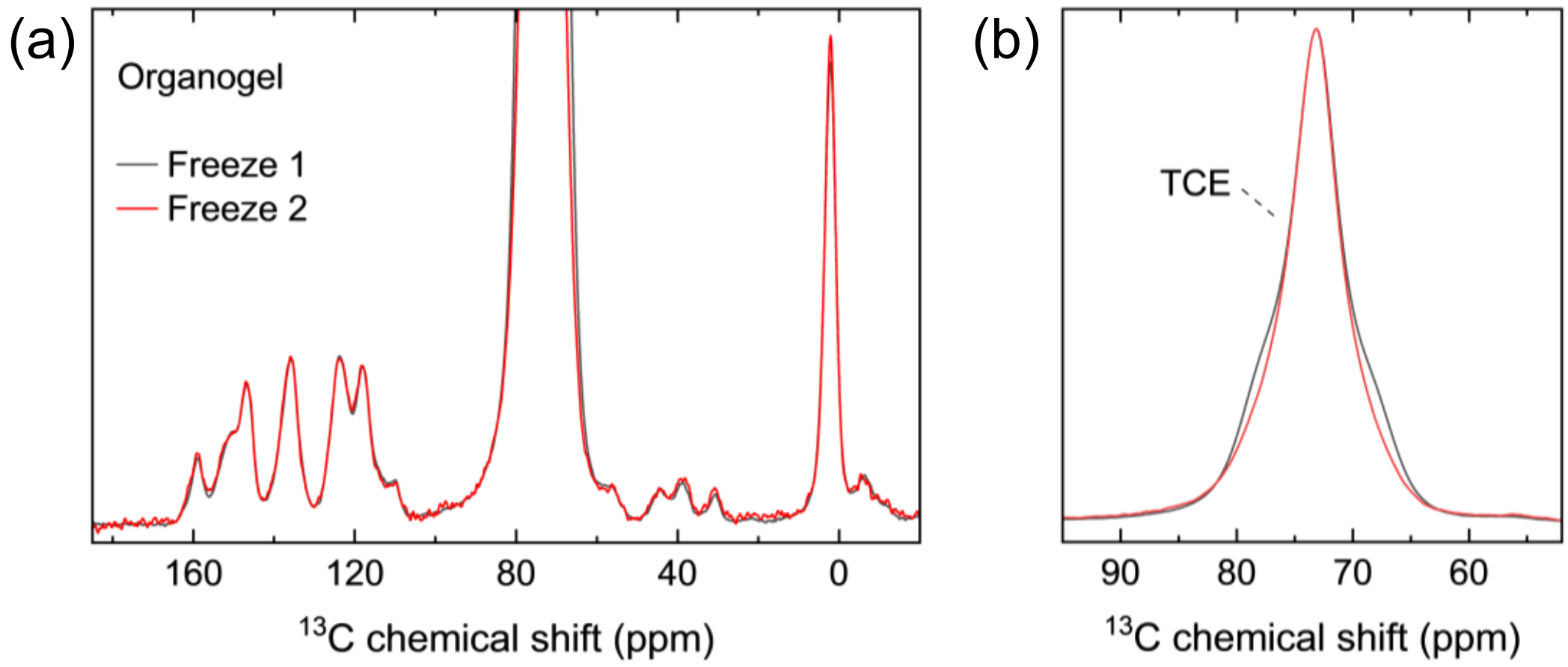

Figure S44: ^{1}H-^{13}C CP MAS DNP NMR spectra (9.4 T, 8 kHz, 100 K) of the pristine organogel. The same sample was rapidly frozen to 100 K to form a glass, NMR measured, thawed` at RT (gel reforms), then rapidly frozen to 100 K again to form a glass and NMR measured for a second time. (a) Full spectrum, and (b) the TCE signal; note that no splitting of the TCE peak by ^{13}C-$^{35/37}Cl$ residual dipolar coupling is observed which indicates in both cases TCE forms a glass. The signals corresponding to the gel are unaffected.

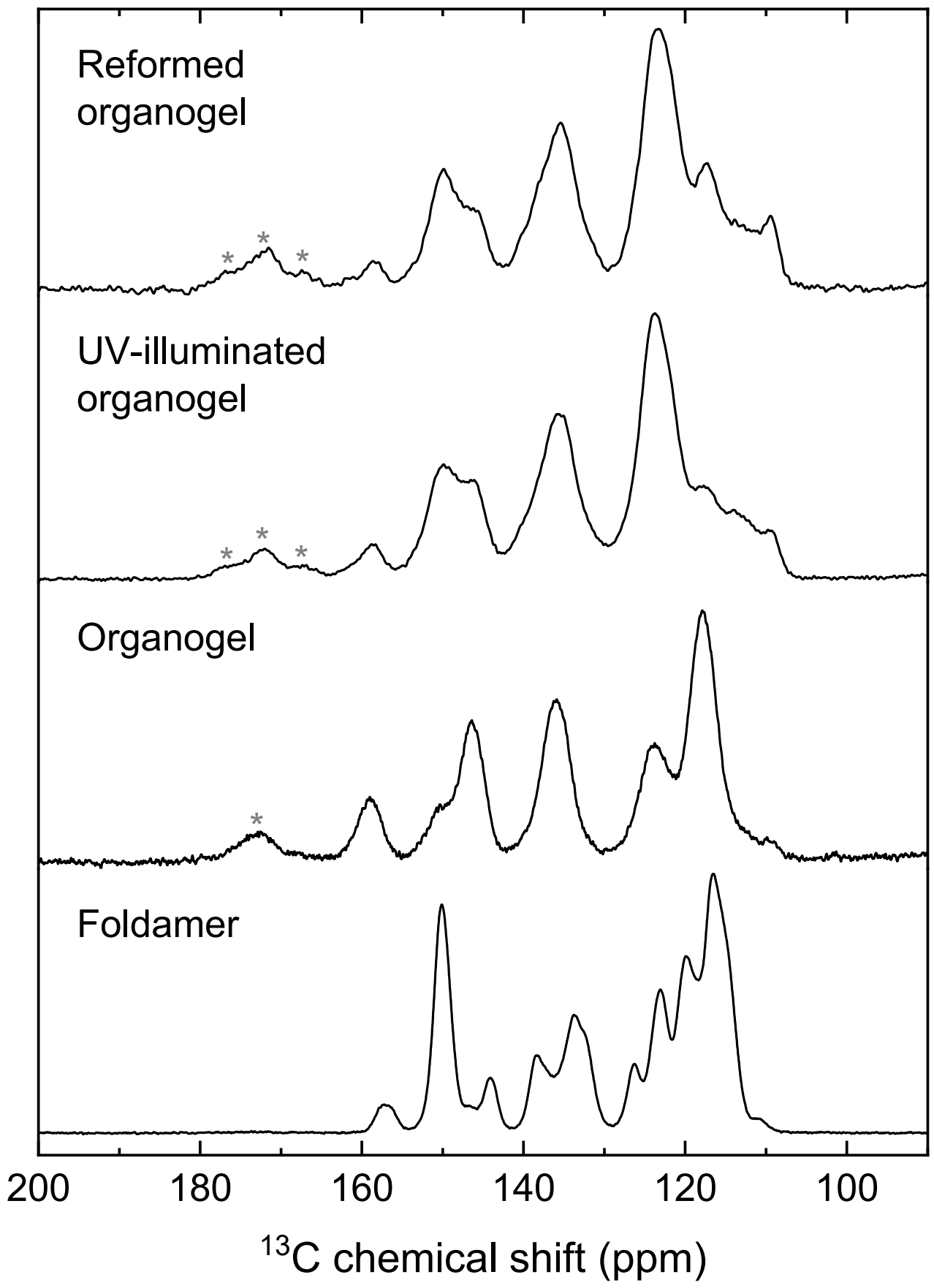

Figure S45: ^{1}H-^{13}C cross-polarisation magic-angle spinning dynamic nuclear polarisation nuclear magnetic resonance (CP MAS DNP NMR) spectroscopy (9.4 T, 10 kHz) of the foldamer, metallogel, UV-illuminated metallogel and the thermally healed metallogel. ^{1}H decoupling is applied during these measurements. For full experimental details, see Methods and **Table S1**. Asterisks denote spinning sidebands.

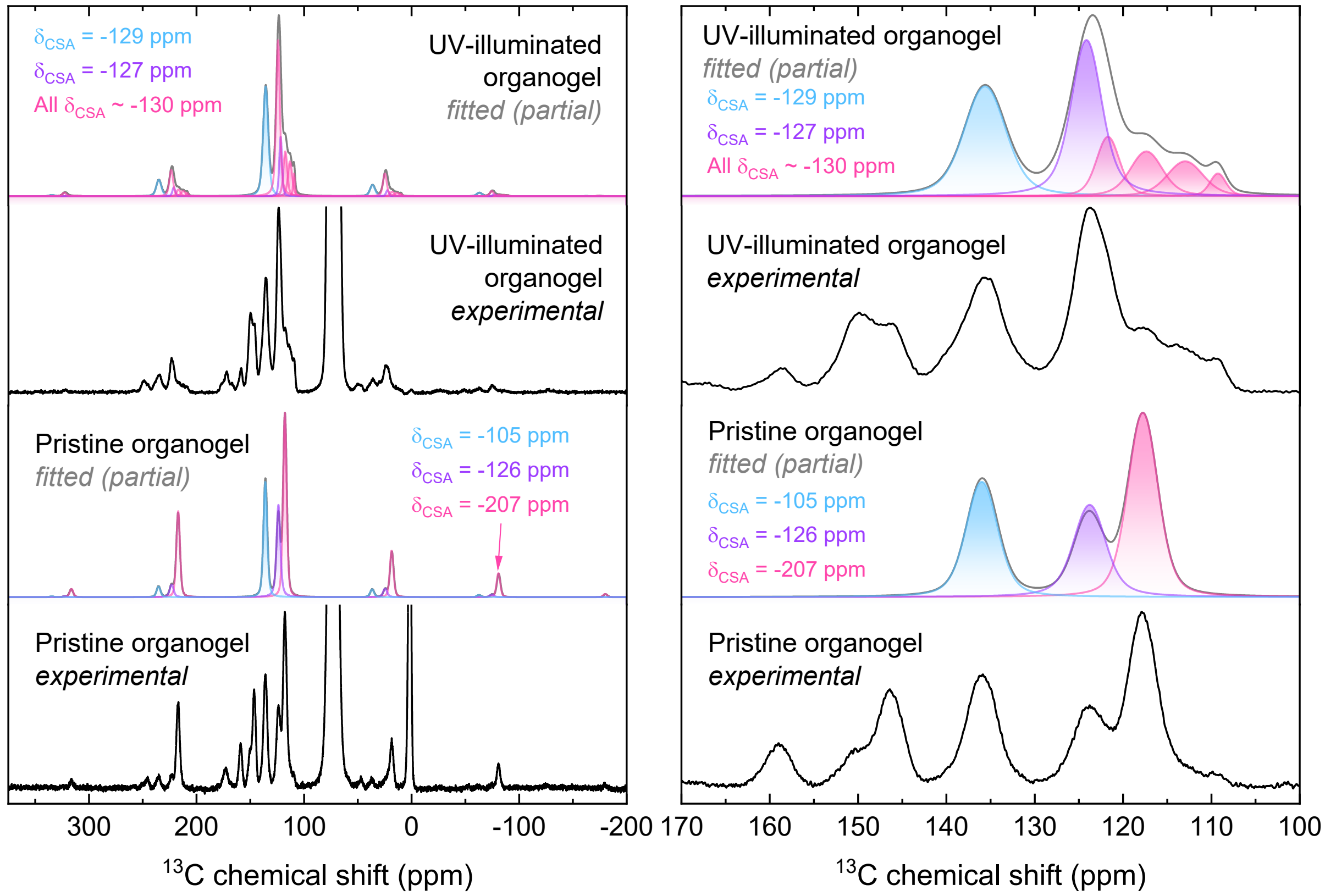

Figure S46: ^{1}H-^{13}C cross-polarisation magic-angle spinning dynamic nuclear polarisation nuclear magnetic resonance (CP MAS DNP NMR) spectroscopy (9.4 T, 10 kHz) of the metallogel and UV-illuminated metallogel. Selected signals are fitted using the sola function in Topspin 4.3.0, and fitted chemical shift anisotropy (δ_{CSA}) values are given on the figure. The value of CSA asymmetry (η_{CSA}) for the signal with δ_{CSA} = -207 ppm is η_{CSA} = 0.208. For all other signals, η_{CSA} < 0.1. For full experimental details, see Methods and **Table S1**. Asterisks denote spinning sidebands.

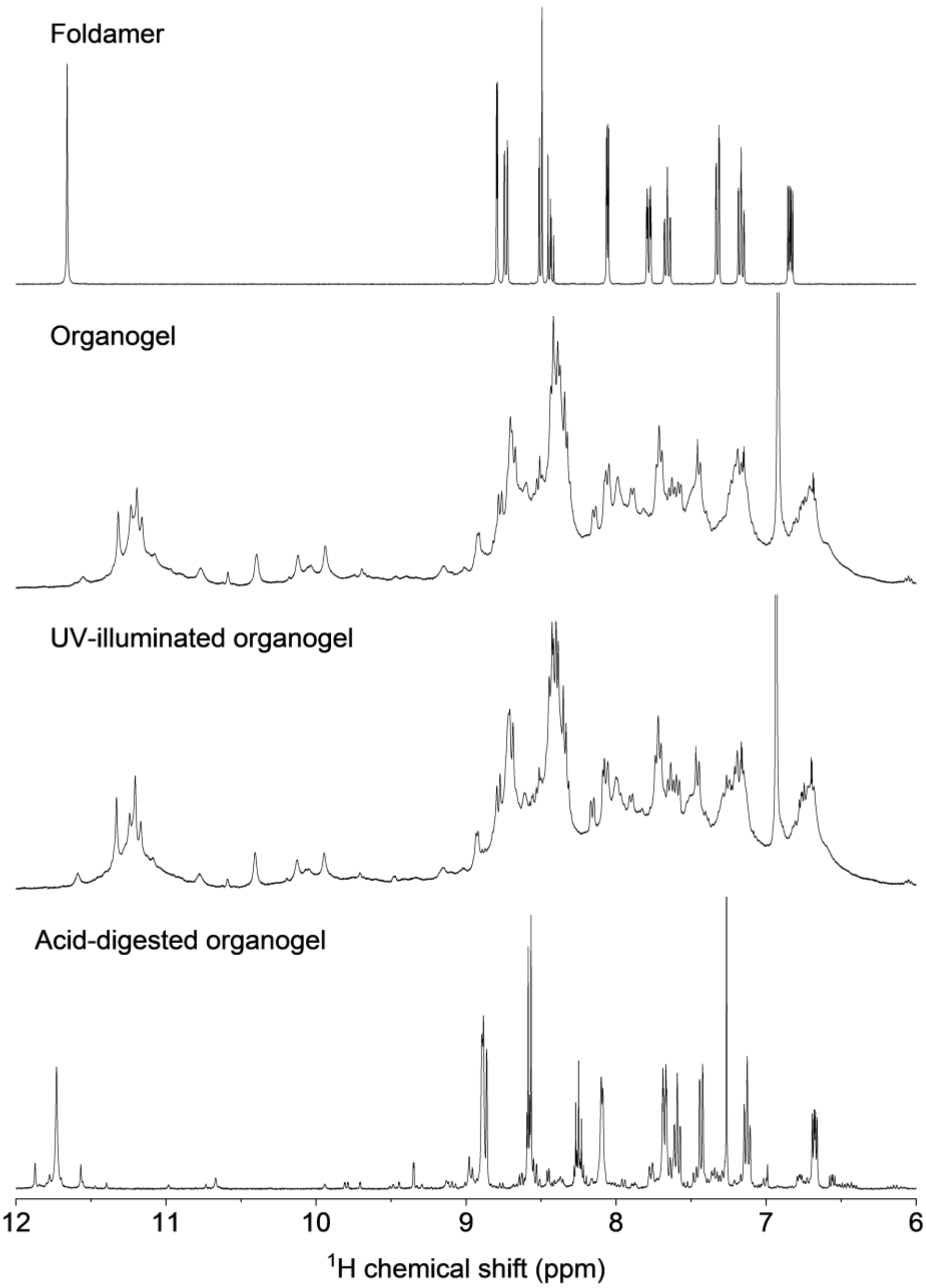

Figure S47: Liquid-state ^{1}H NMR of foldamer, organogel, UV-irradiated organogel (sol) and acid-digested organogel dissolved in DMSO-d_6. Note, presence of acetonitrile and tetrachloroethane from gel cause slight changes in ^{1}H chemical shifts compared to those expected in pure DMSO. Similarly, acid-digested organogel contains substantial remnant aqueous HCl, causing differences in ^{1}H shifts.

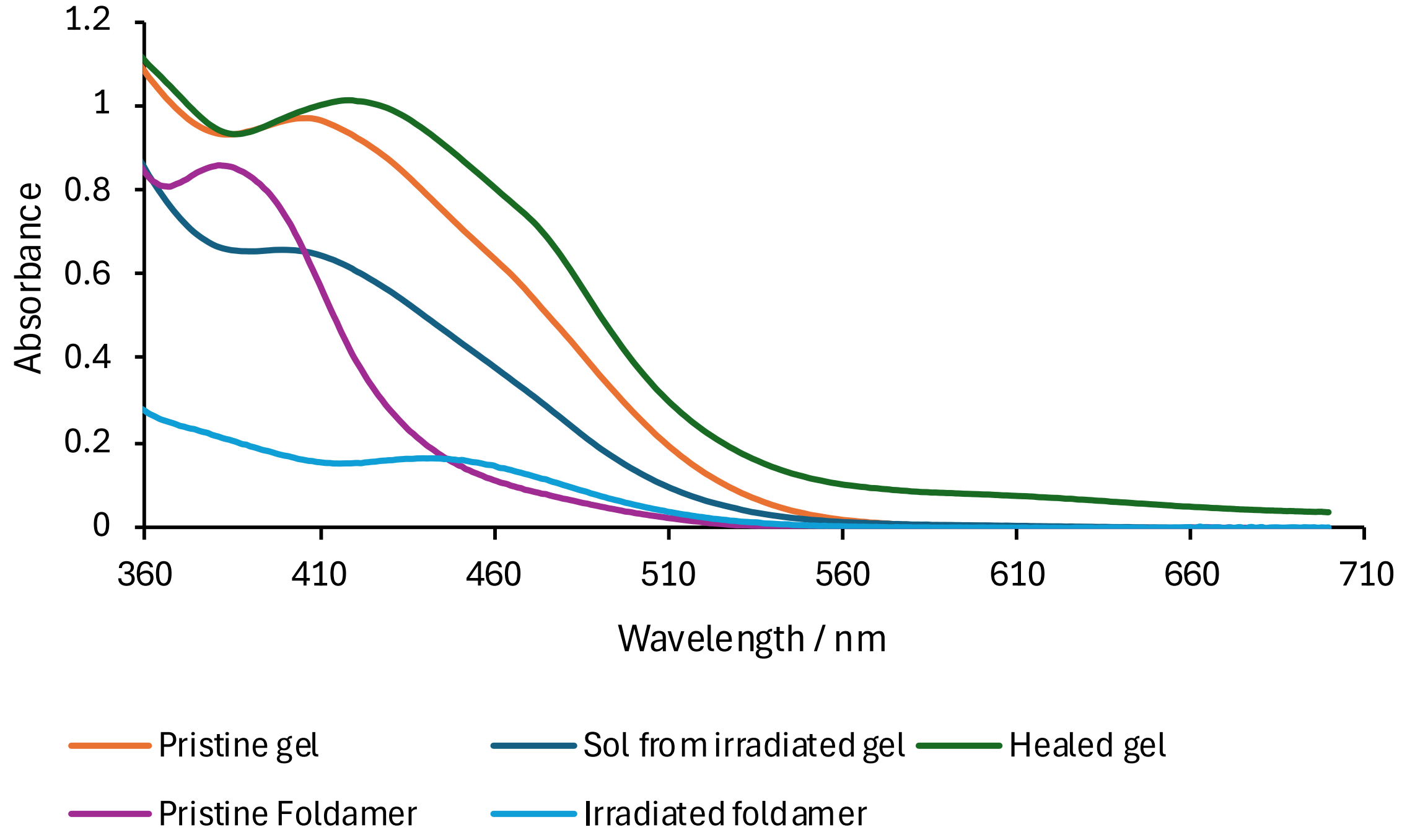

Figure S48: UV-vis spectra for the pristine gel, sol from the irradiated gel and the thermally healed gel. The solution data for the pristine foldamer and irradiated foldamer are also shown for reference. The large width of the gel spectra masks the *E* and *Z* fingerprint regions at 400 and 450 nm respectively, precluding determination of the E/Z ratio.

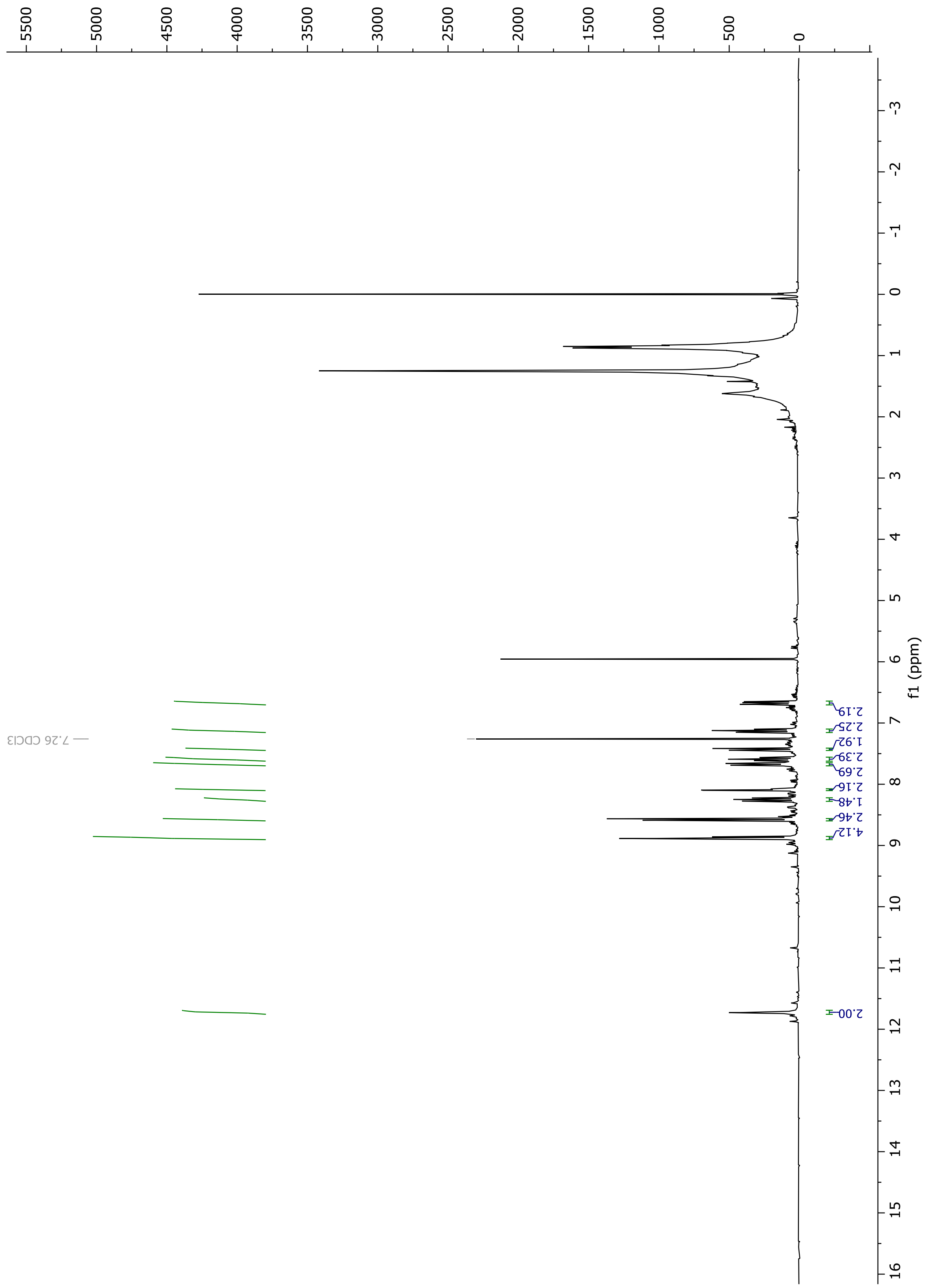

Figure S49: ^1H NMR spectrum (400 MHz, $CDCl_3$, 298 K) of 6 wt.% (g/mL) metallogel formed in 3:7 acetonitrile: tetrachloroethane after overnight degradation in 1M HCl and attempted purification via column chromatography.

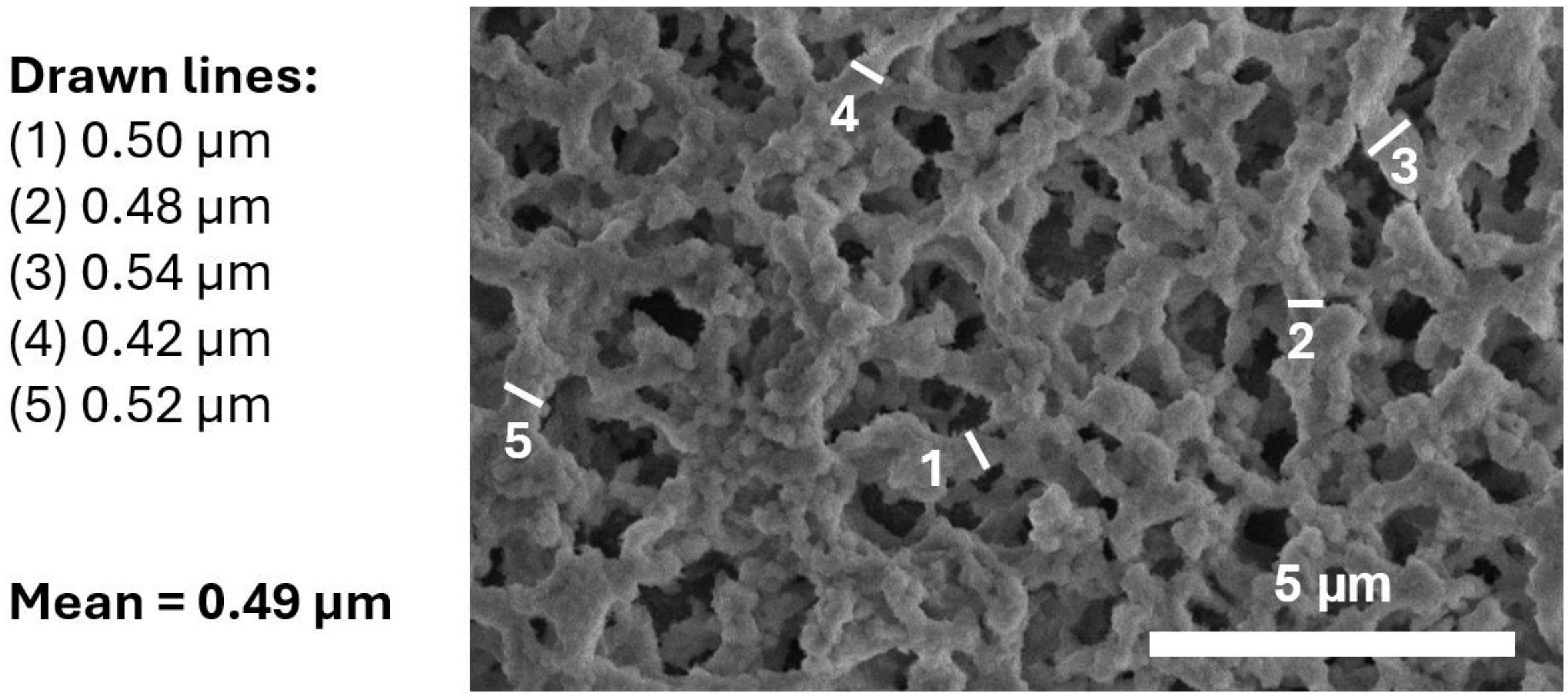

Figure S50: Measurement of fiber sizes within the hydrogel and calculation of the mean fiber size of 0.49 µm.

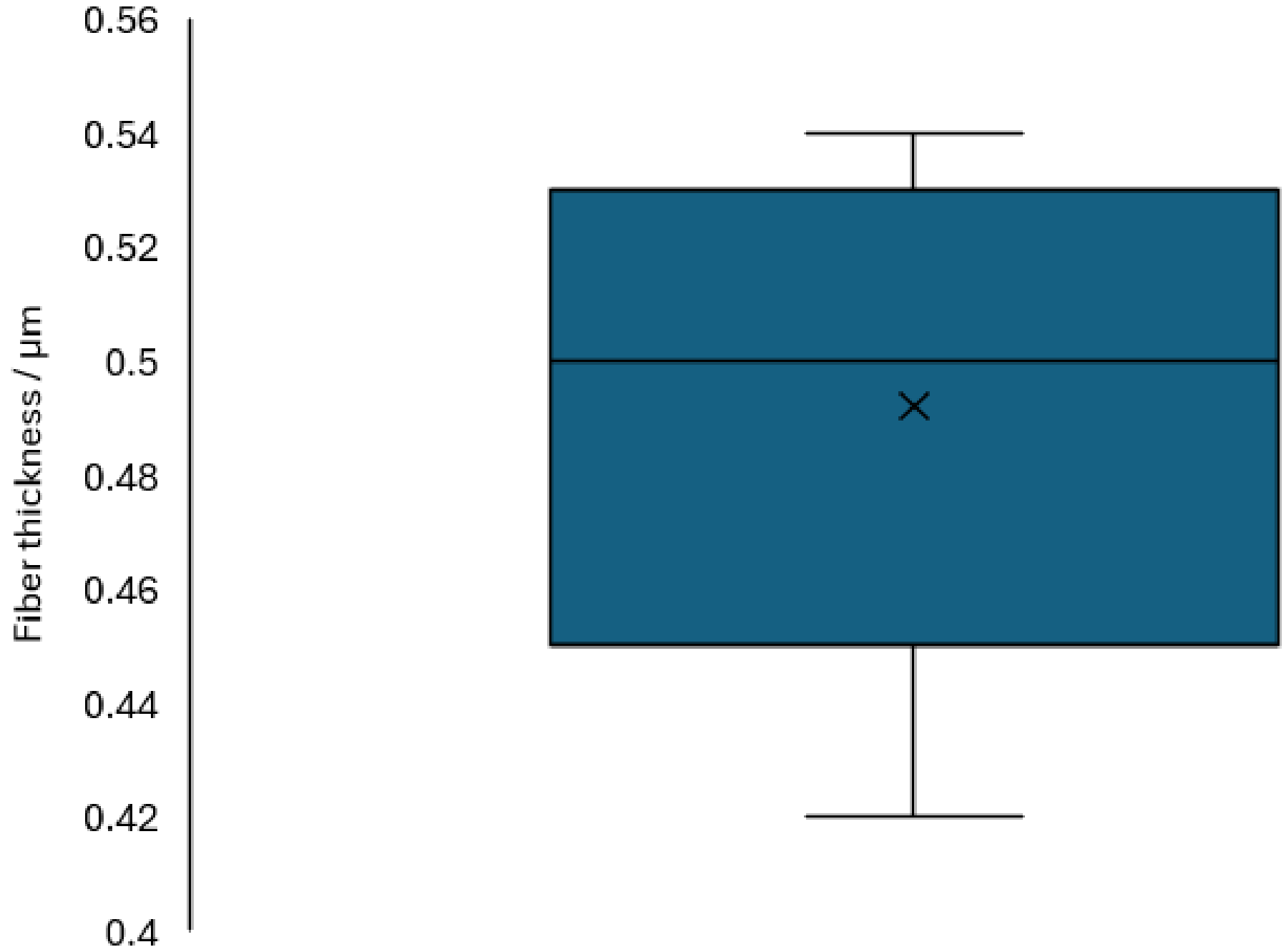

Figure S51: Box and whisker plot showing the distribution of fiber sizes used to calculate the mean.

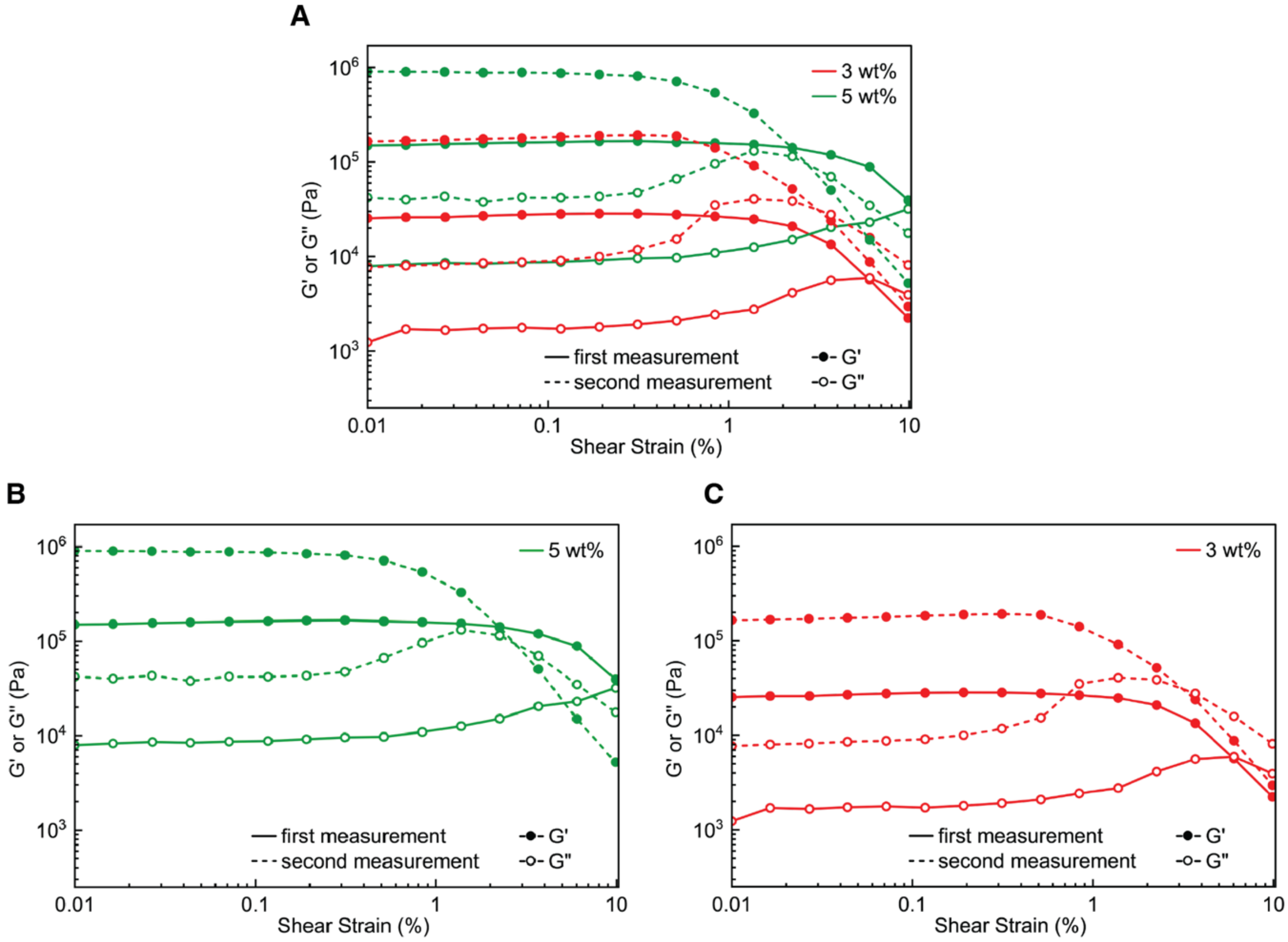

Figure S52: (A) Amplitude sweep tests of the hydrogels at 3 and 5 wt.% to determine the Linear Viscoelastic Region (LVER). The storage modulus (G') and loss modulus (G'') are plotted against shear strain (0.1-10%); (B) and (C) - same dataset, plotted for each sample separately.

Appendix A: Liquid-state ^{1}H, ^{13}C NMR spectra

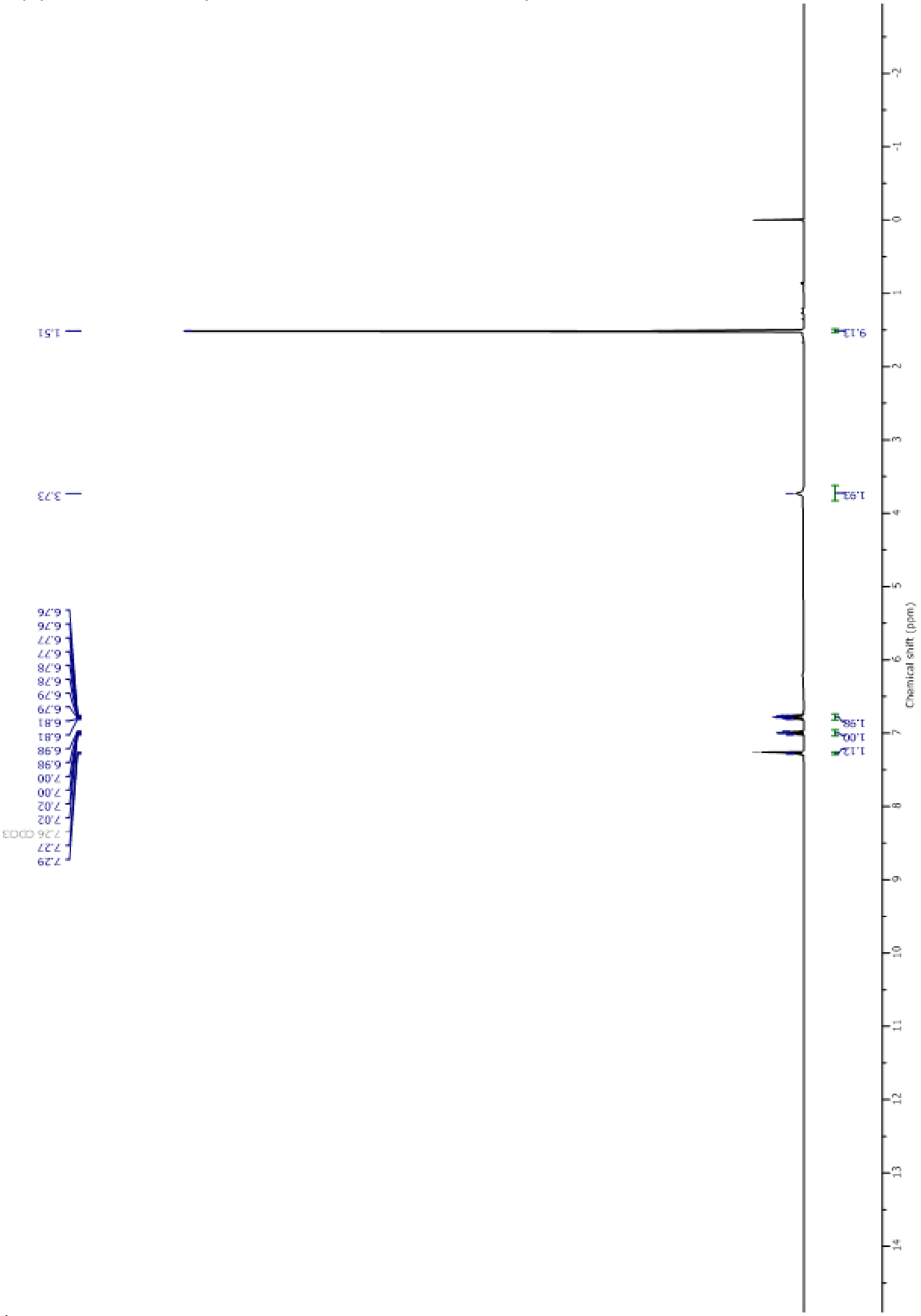

^{1}H NMR spectrum (400 MHz, $CDCl_3$, 298 K) of 3

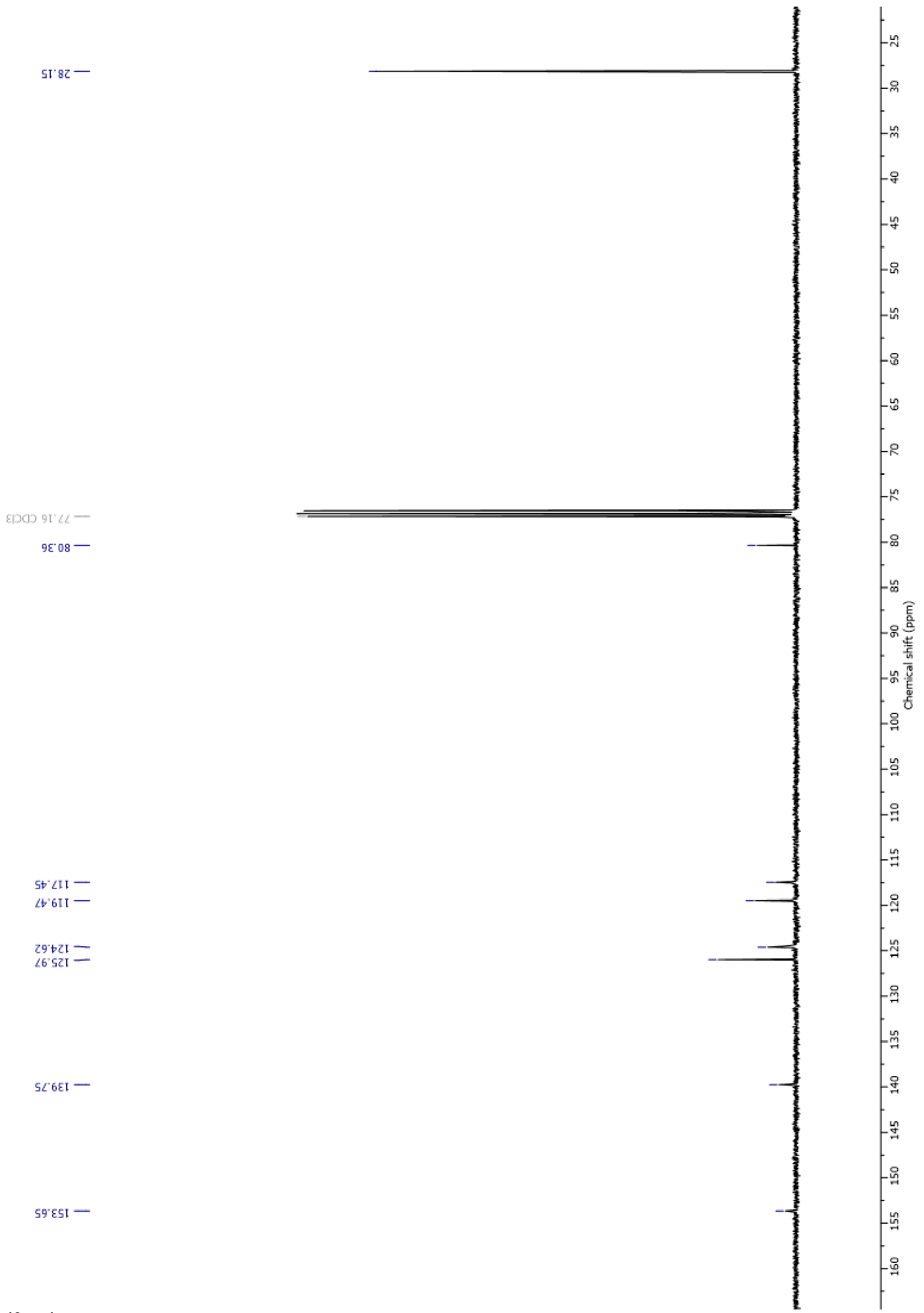

$^{13}C\{^1H\}$ NMR spectrum (101 MHz, $CDCl_3$, 298 K) of 3

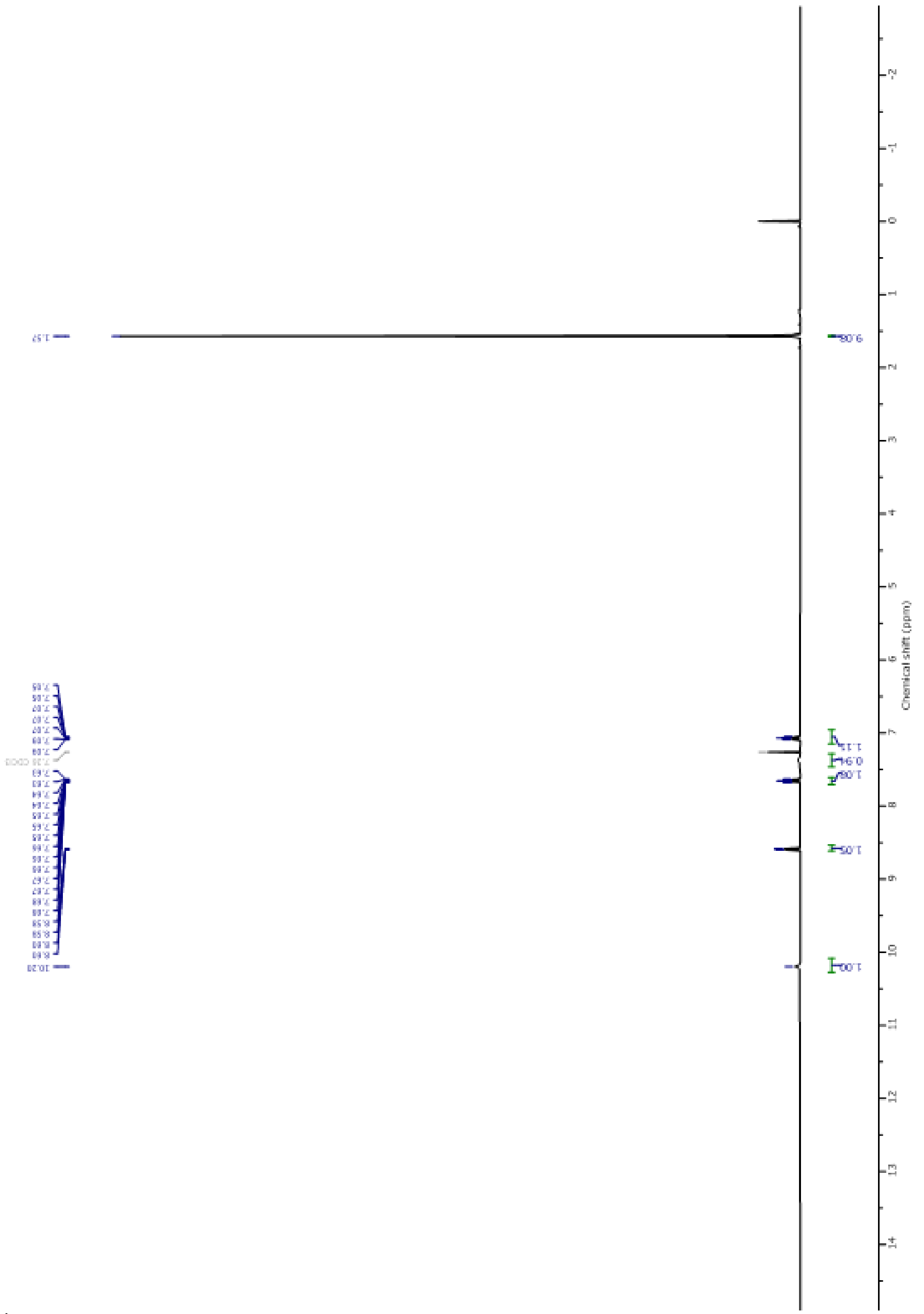

^{1}H NMR spectrum (400 MHz, $CDCl_3$, 298 K) of 4

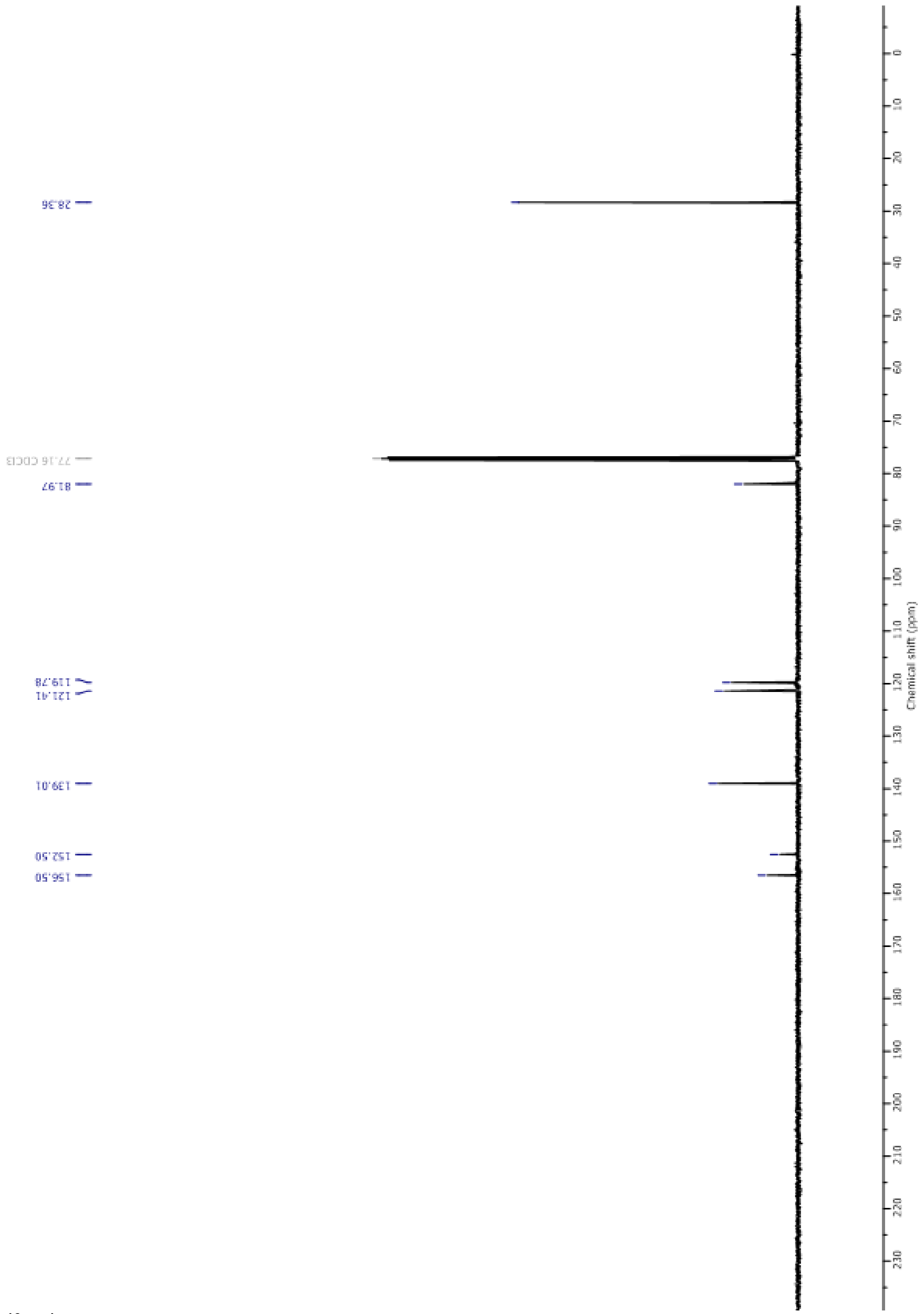

$^{13}C\{^{1}H\}$ NMR spectrum (101 MHz, $CDCl_3$, 298 K) of 4

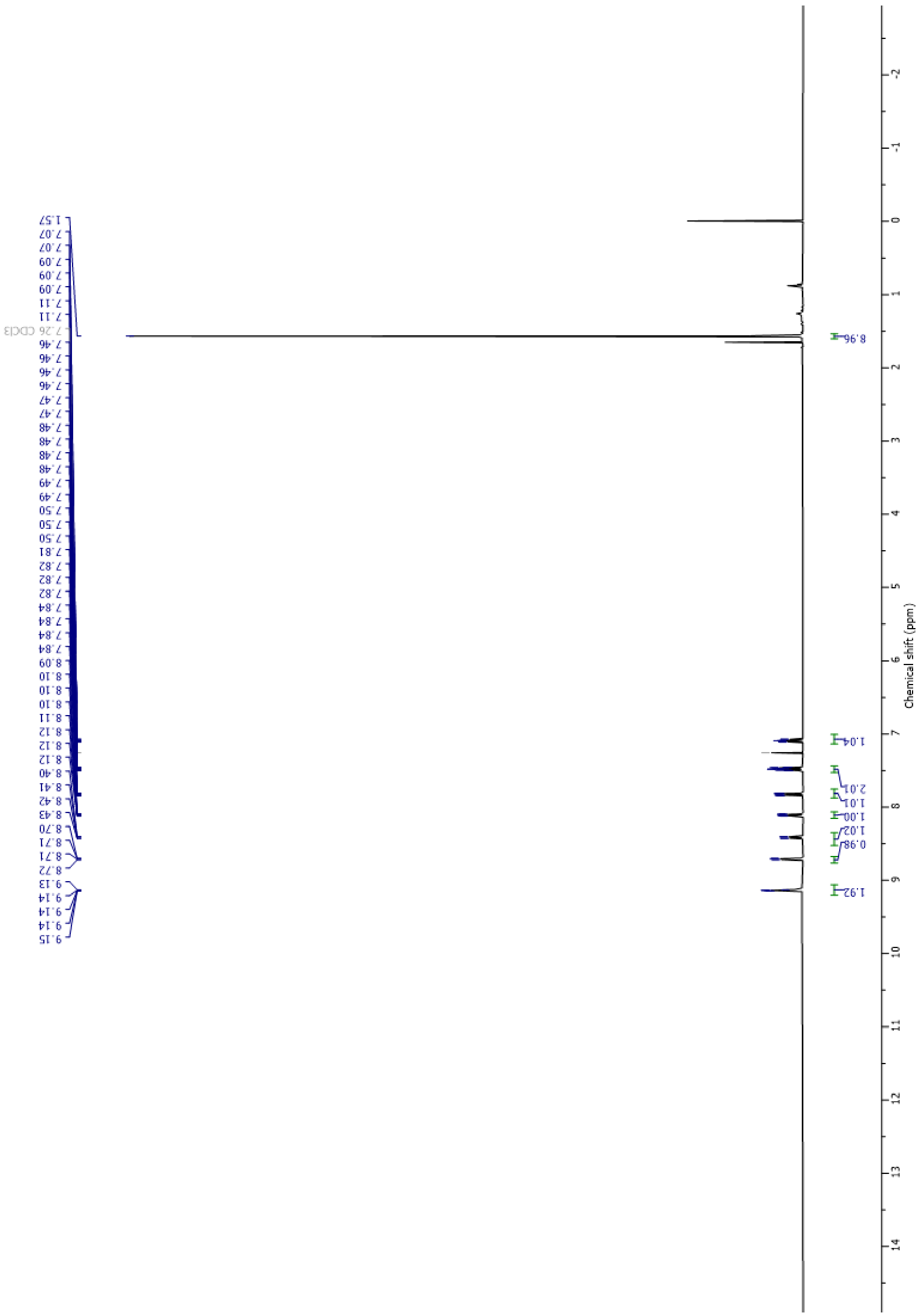

^{1}H NMR spectrum (400 MHz, $CDCl_3$, 298 K) of 5

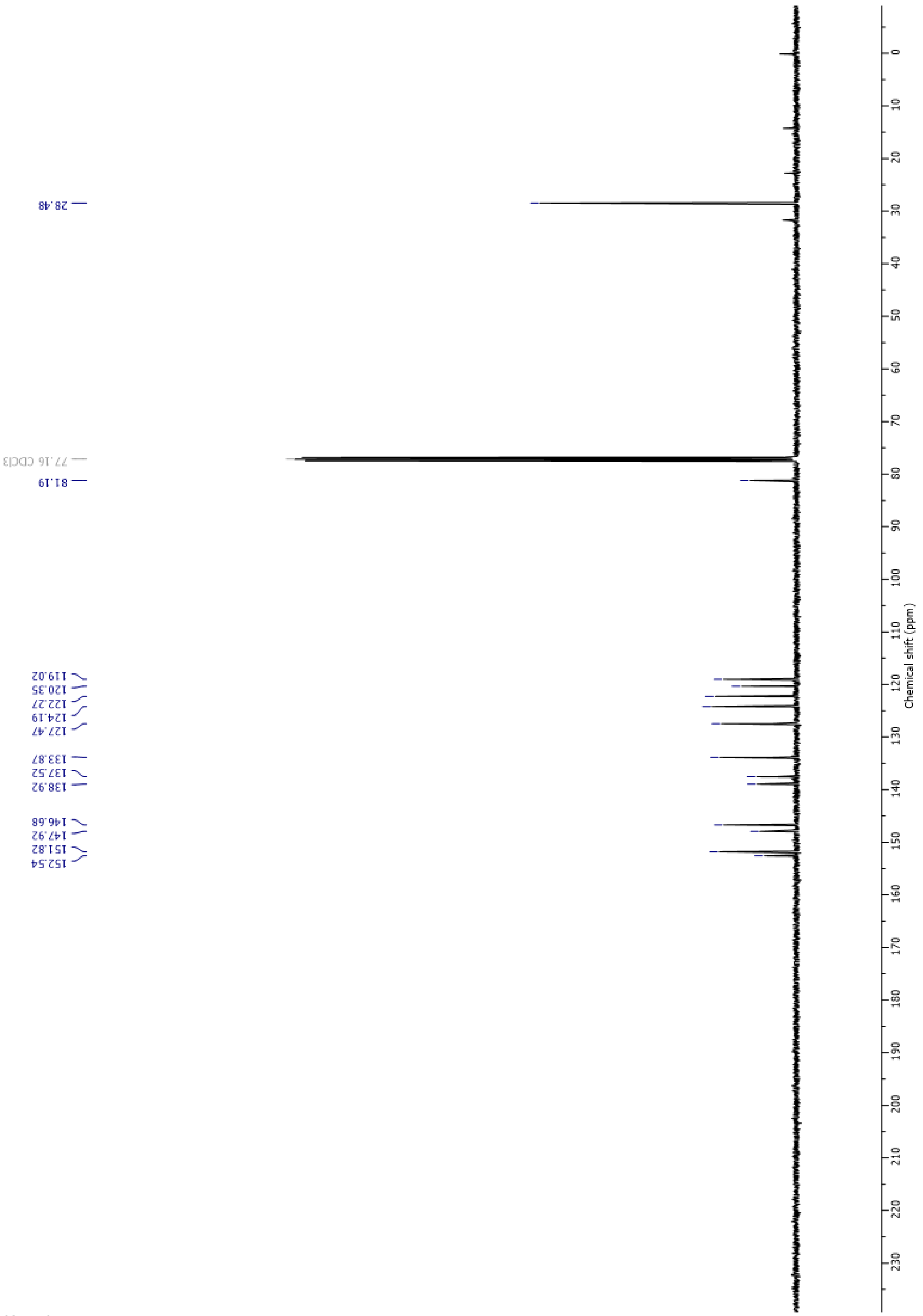

$^{13}C\{^{1}H\}$ NMR spectrum (101 MHz, $CDCl_3$, 298 K) of 5

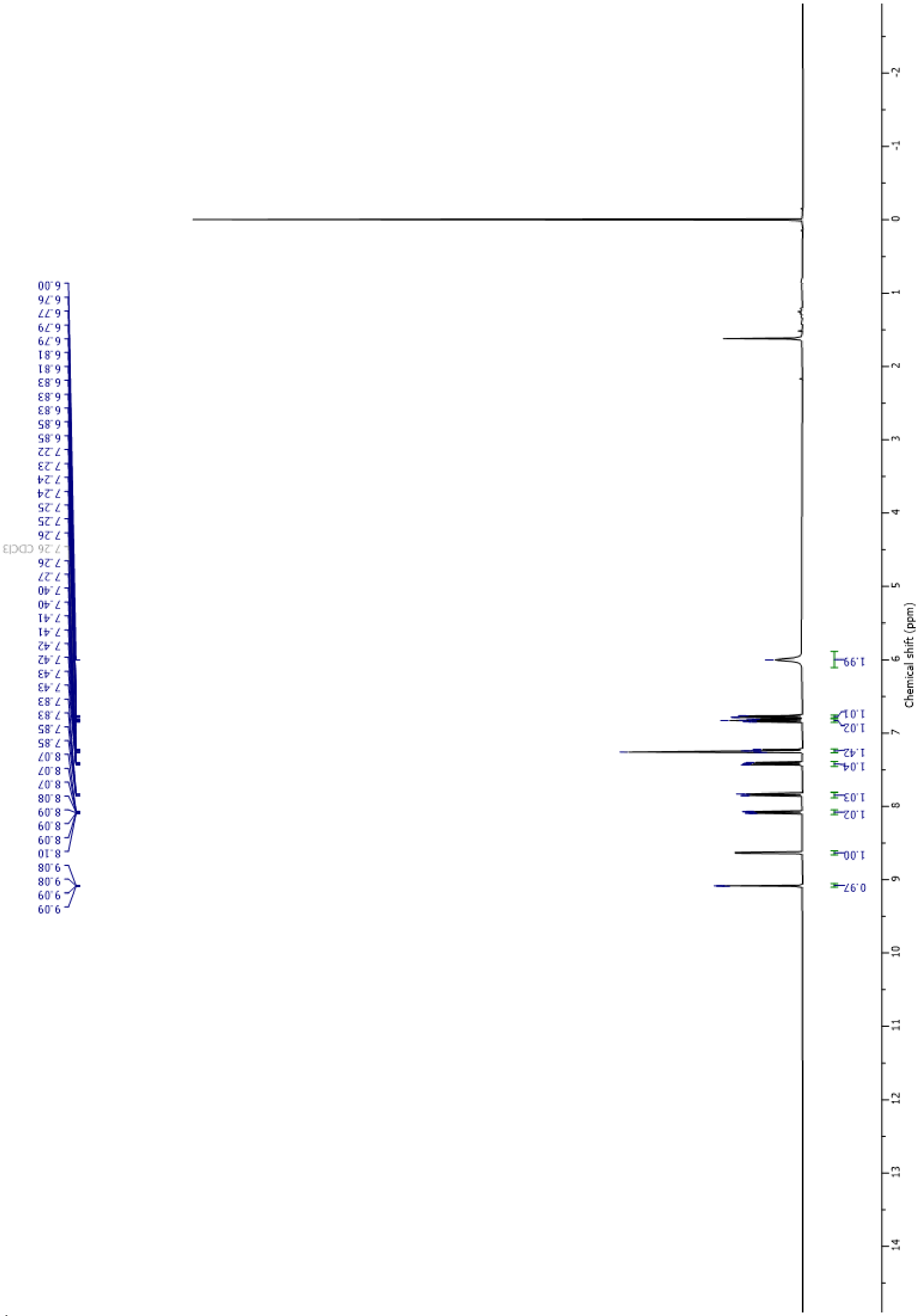

^{1}H NMR spectrum (400 MHz, $CDCl_3$, 298 K) of 6

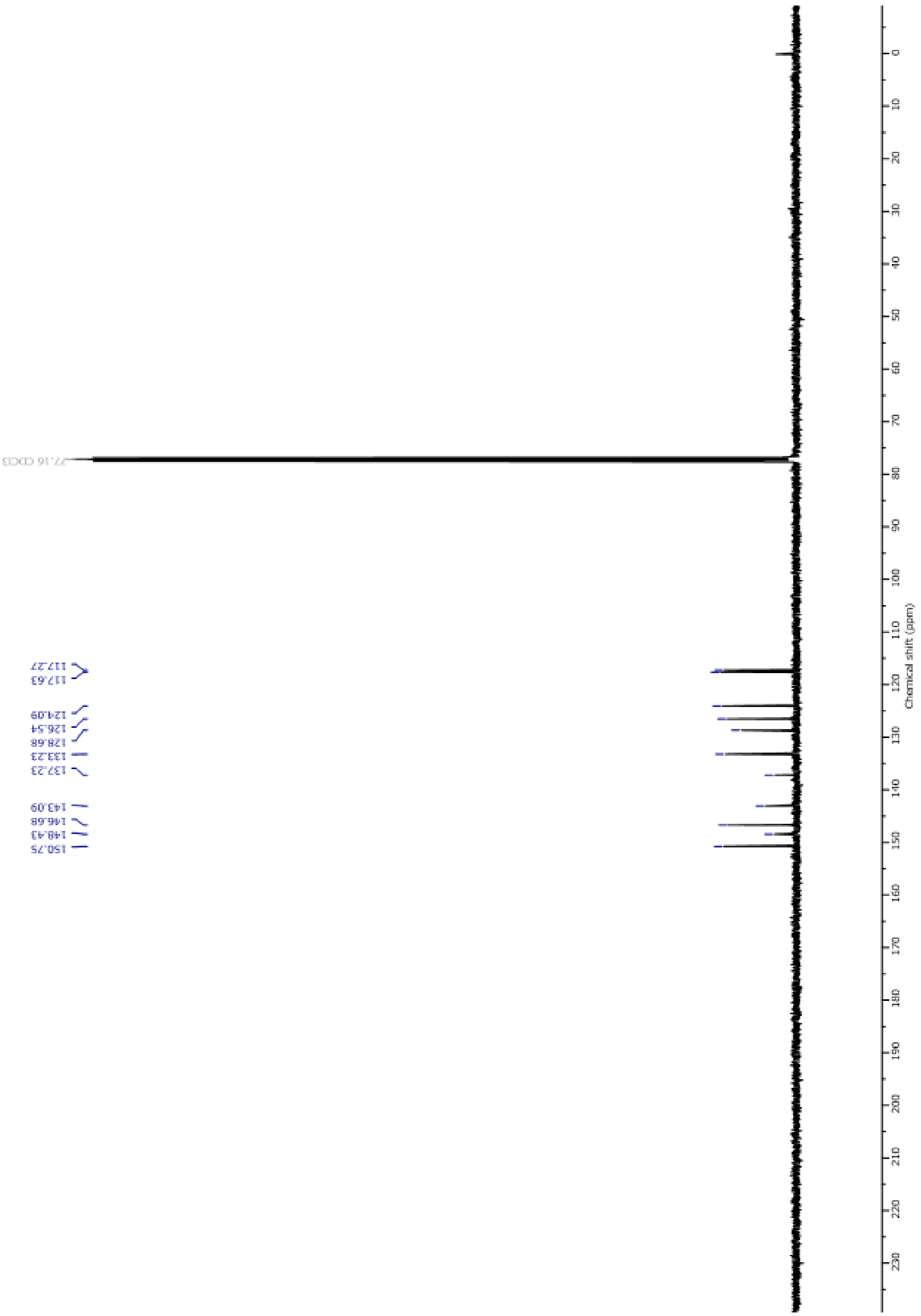

$^{13}C\{^{1}H\}$ NMR spectrum (101 MHz, $CDCl_3$, 298 K) of 6

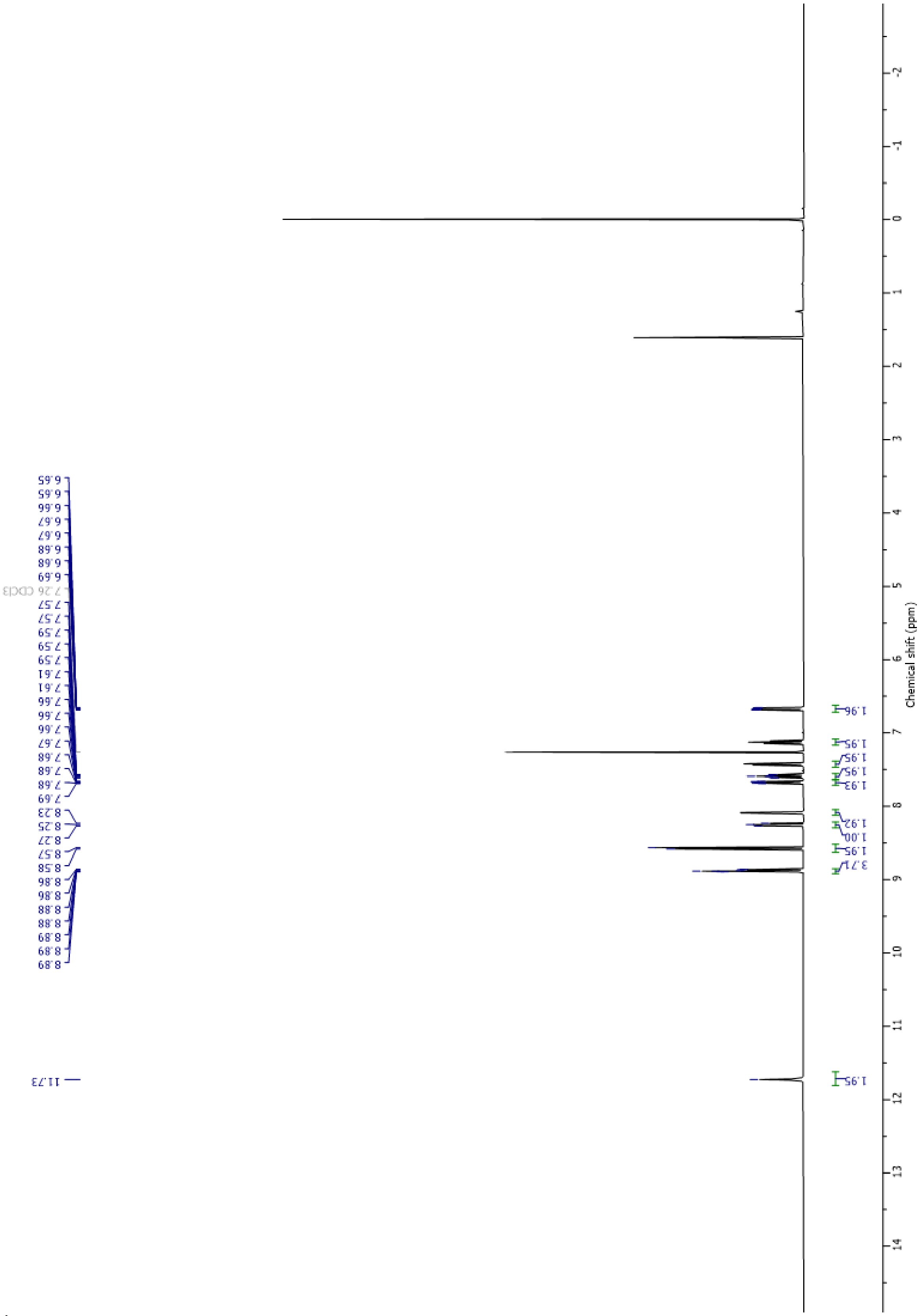

^{1}H NMR spectrum (400 MHz, $CDCl_3$, 298 K) of 1

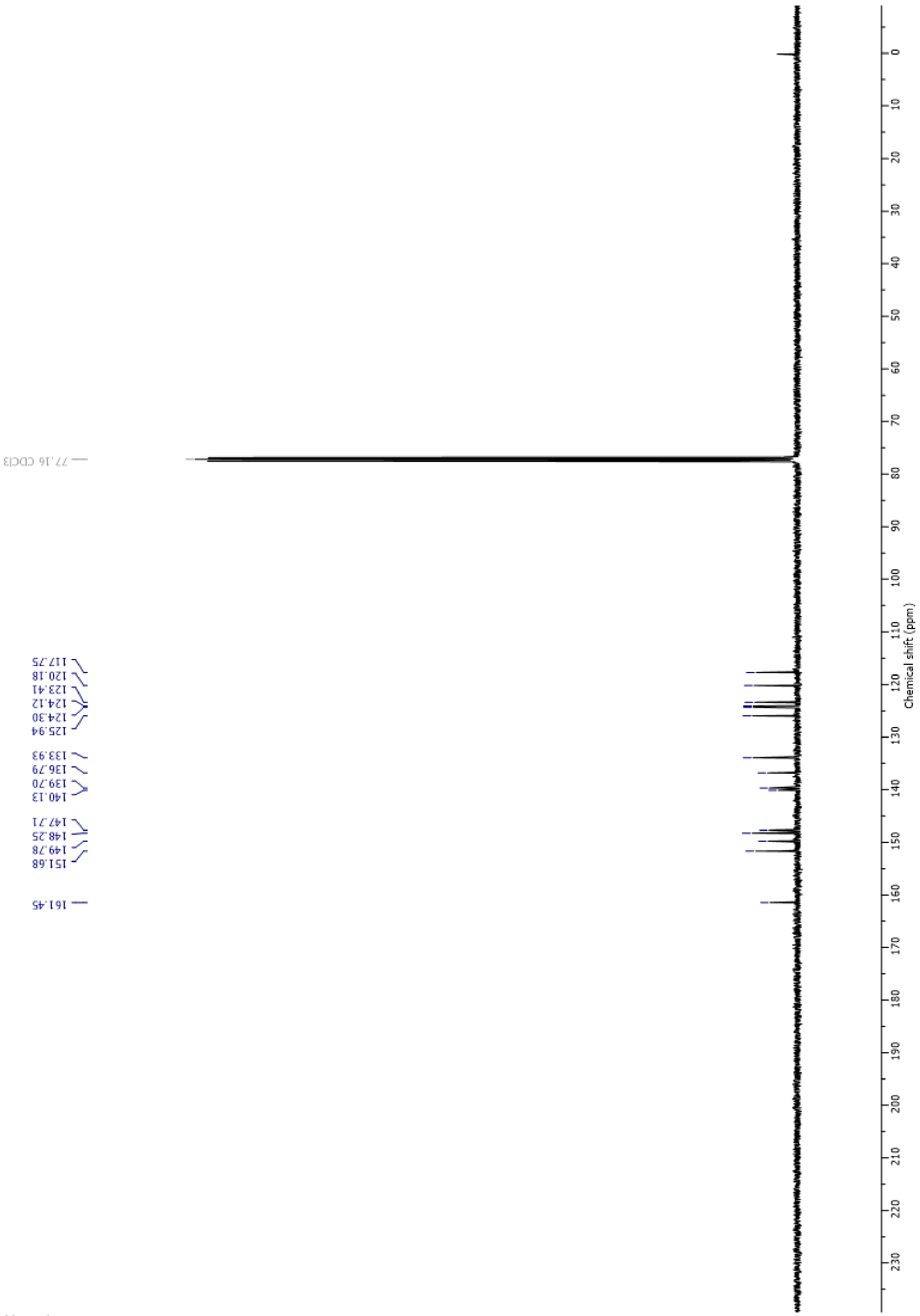

$^{13}C\{^1H\}$ NMR spectrum (101 MHz, $CDCl_3$, 298 K) of 1

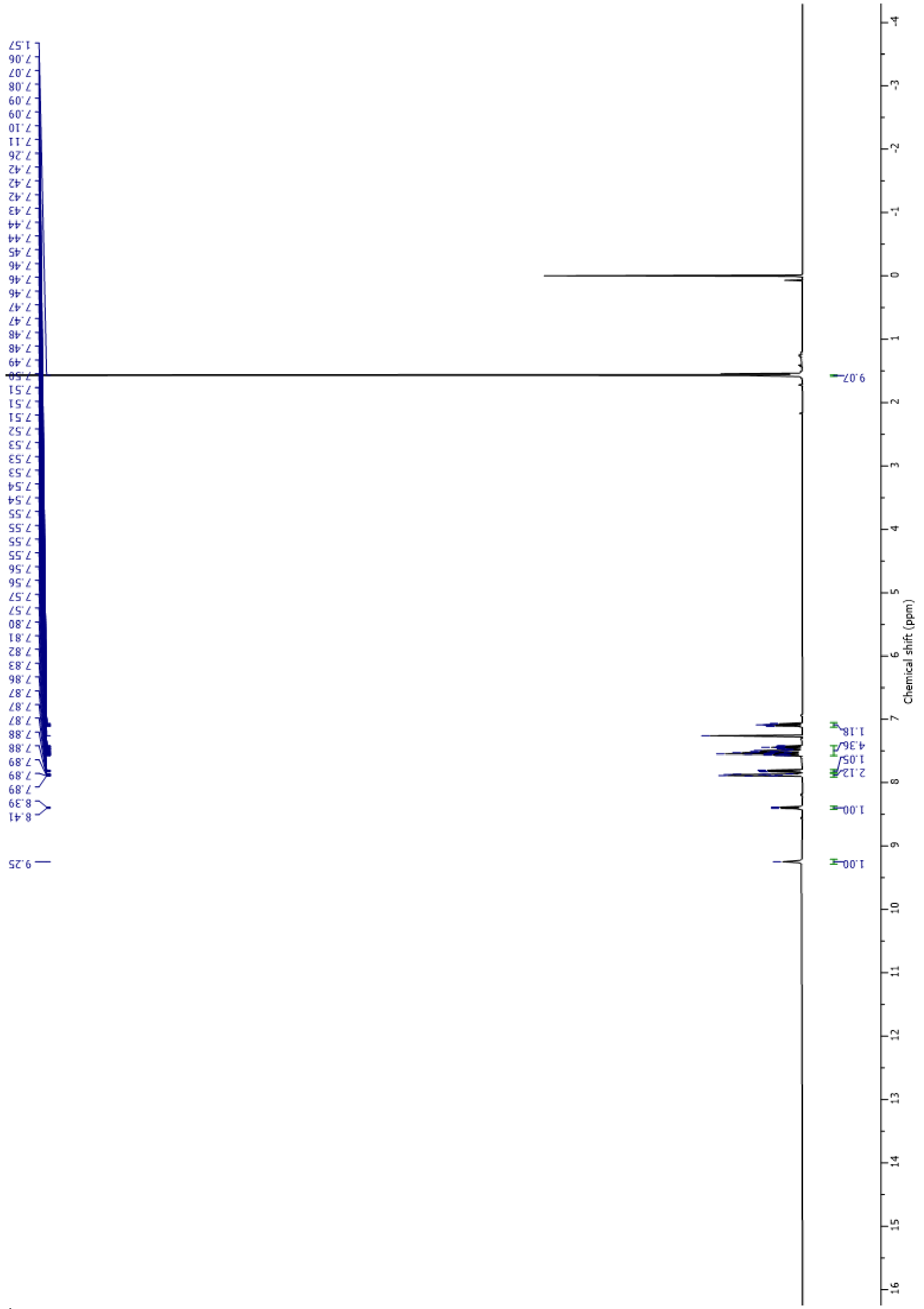

^{1}H NMR spectrum (400 MHz, $CDCl_3$, 298 K) of 7

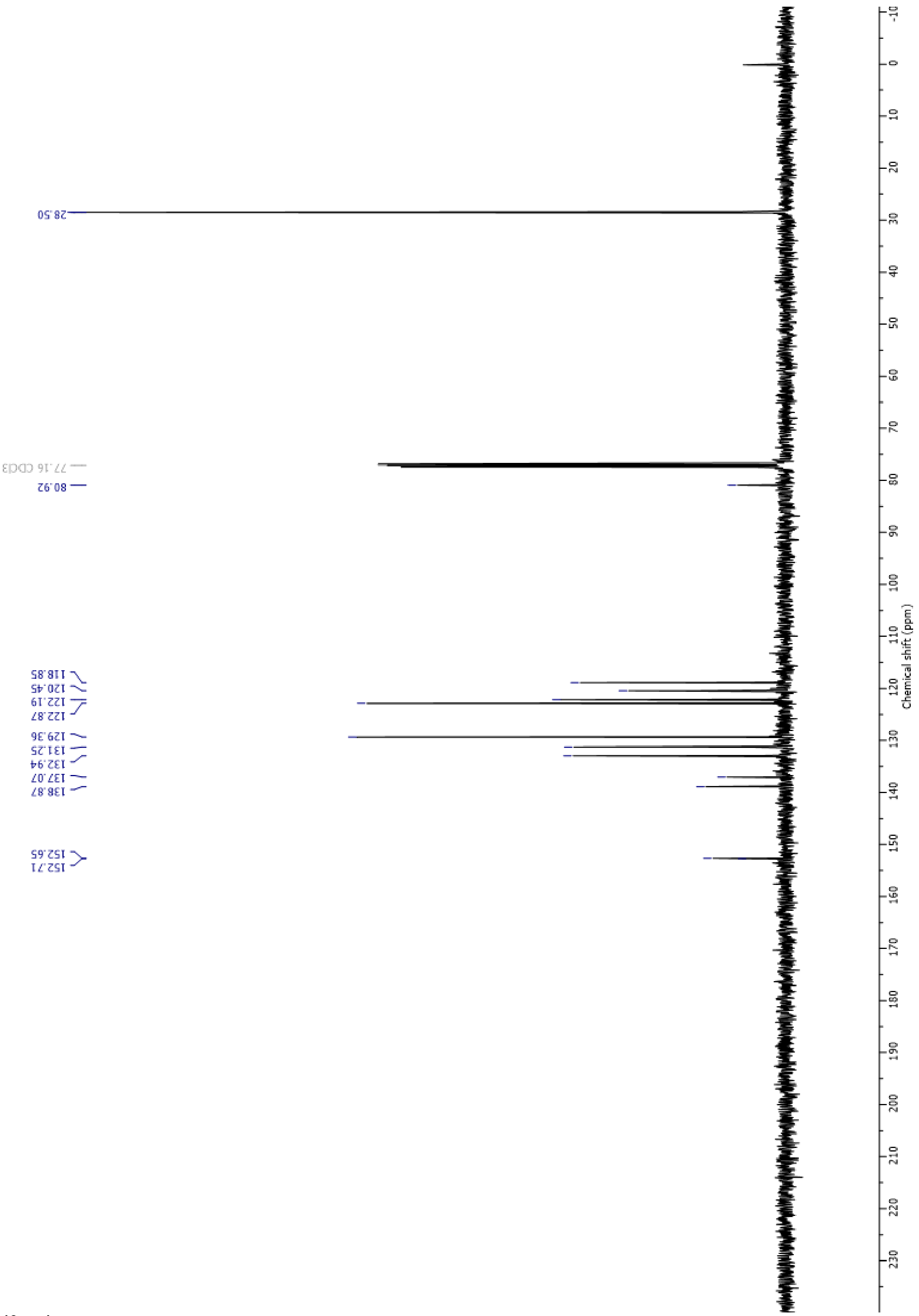

$^{13}C\{^{1}H\}$ NMR spectrum (101 MHz, $CDCl_3$, 298 K) of 7

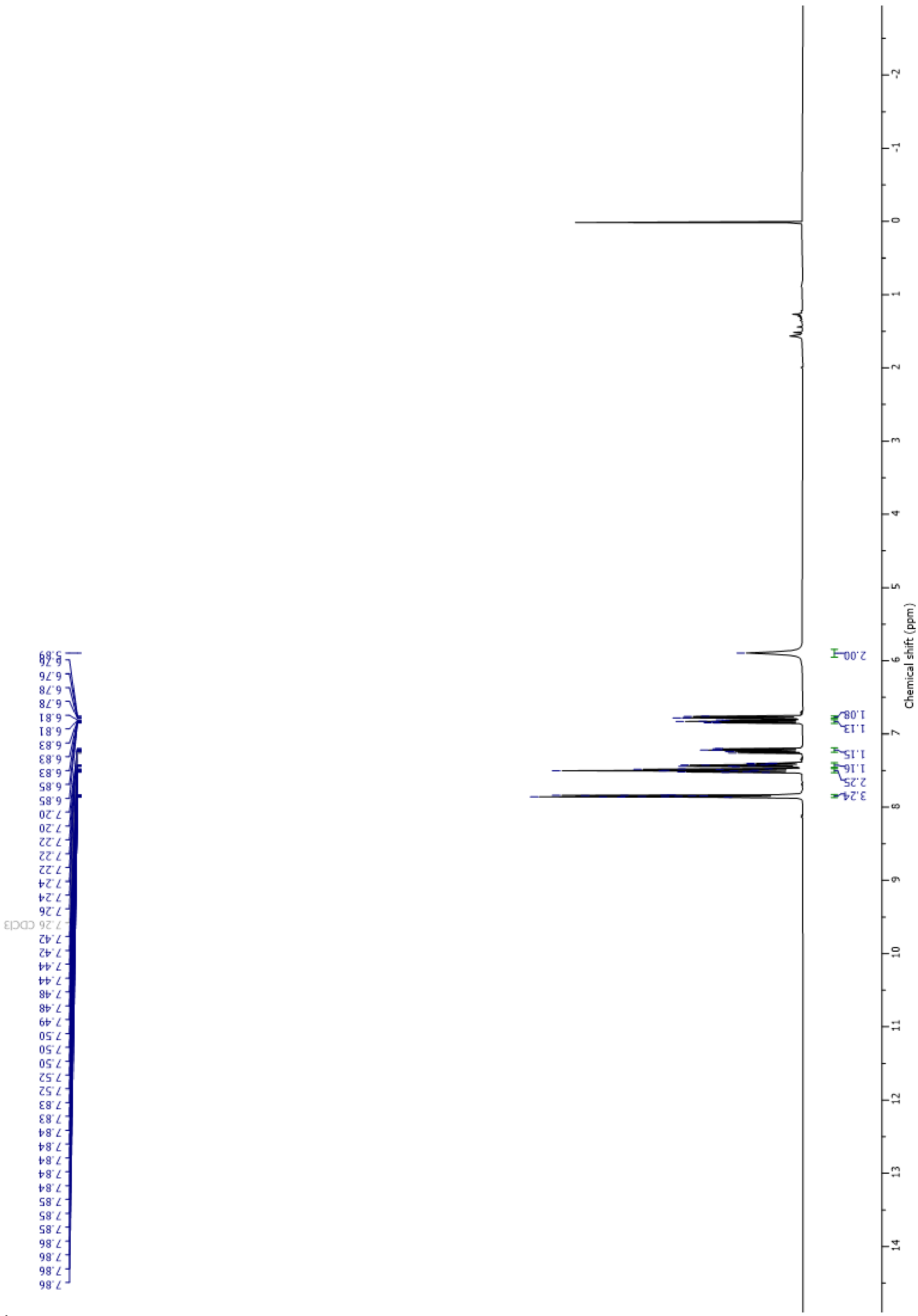

^{1}H NMR spectrum (400 MHz, $CDCl_3$, 298 K) of 8

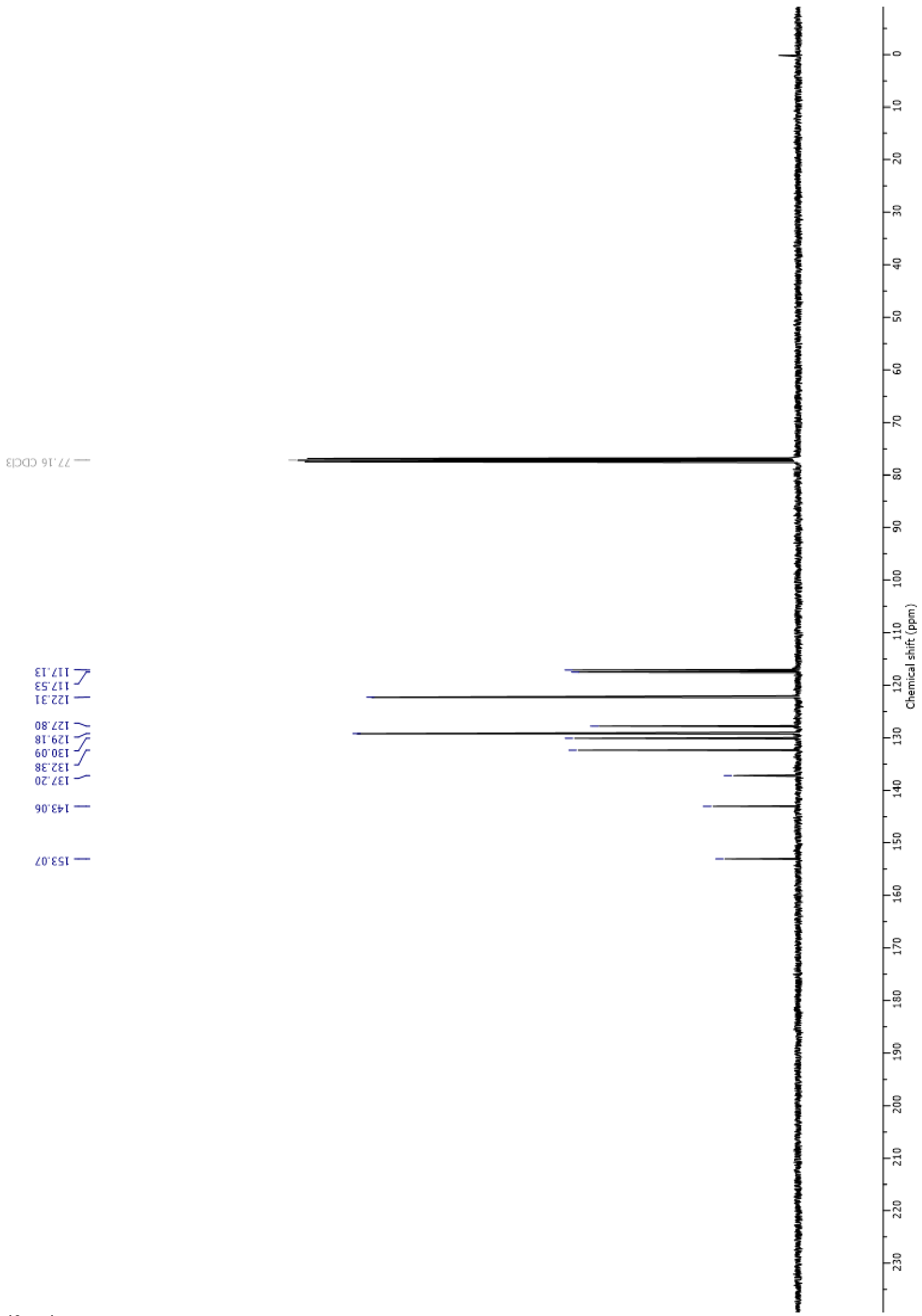

$^{13}C\{^{1}H\}$ NMR spectrum (101 MHz, $CDCl_3$, 298 K) of 8

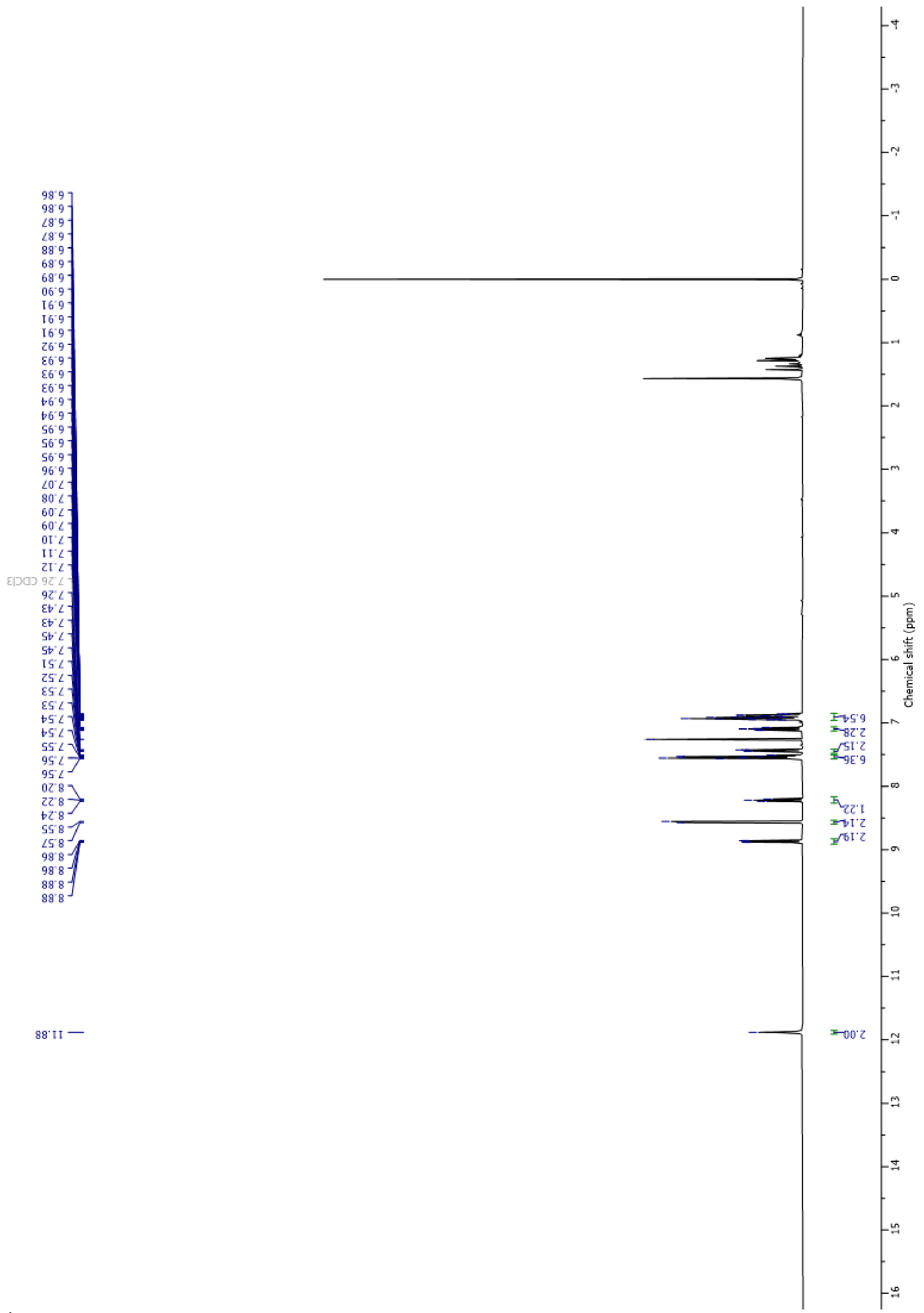

^{1}H NMR spectrum (400 MHz, $CDCl_3$, 298 K) of 2

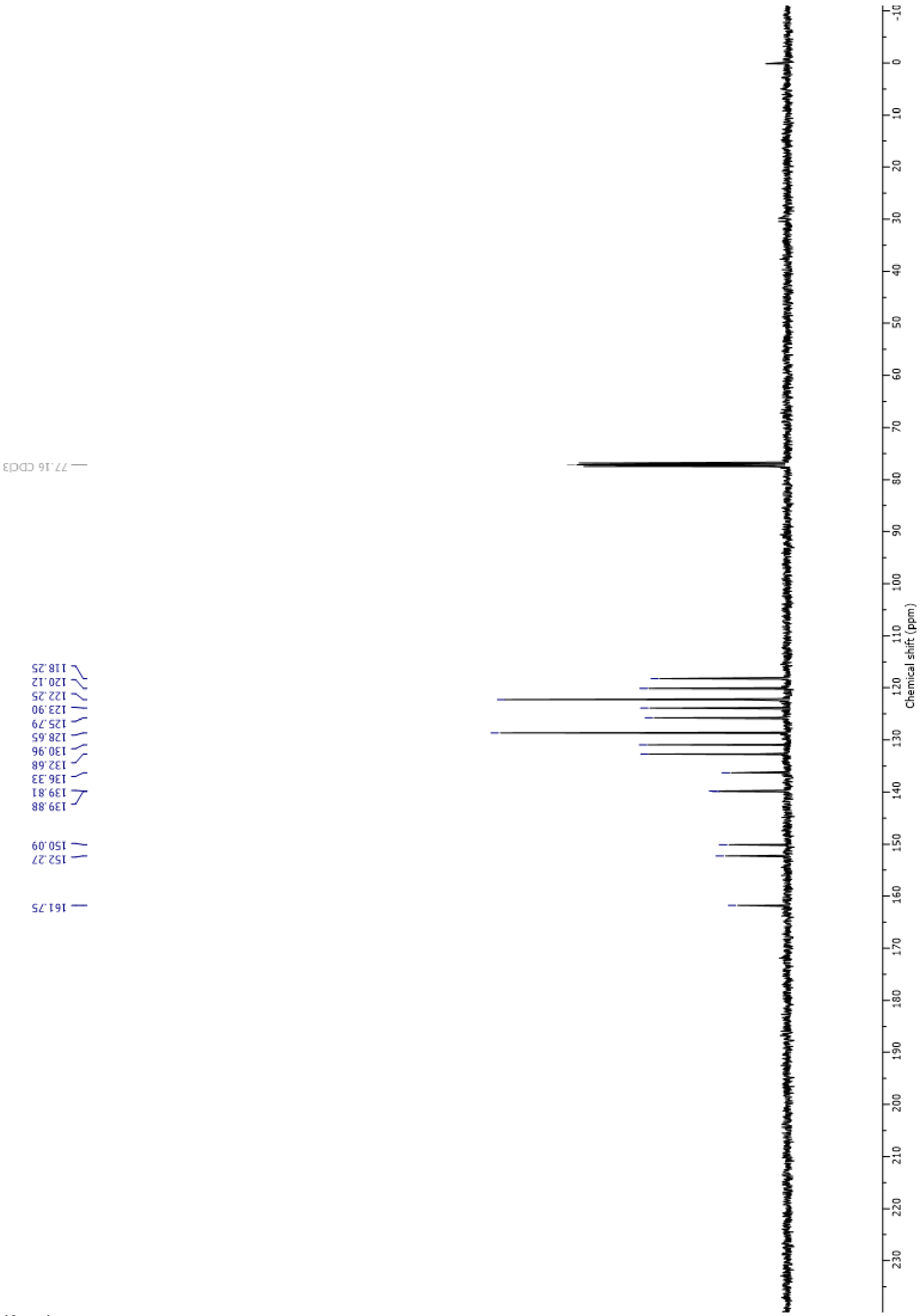

$^{13}C\{^{1}H\}$ NMR spectrum (101 MHz, $CDCl_3$, 298 K) of 2

References

1 D. Kalia, S. P. Pawar and J. S. Thopate, *Ang. Chem. Int. Ed.*, 2017, **56**, 1885–1889.
2 S. J. Pike, A. Heliot and C. C. Seaton, *CrystEngComm*, 2020, **22**, 5040–5048.
3 T. Hu, G. Zheng, D. Xue, S. Zhao, F. Li, F. Zhou, F. Zhao, L. Xie, C. Tian, T. Hua, S. Zhao, Y. Xu, G. Zhong, Z.-J. Liu, A. Makriyannis, R. C. Stevens and H. Tao, *J. Med. Chem.*, 2021, **64**, 13752–13765.
4 Q. Liu, H. Chu, J. Mai, H. Yang, M.-H. Shen and H.-D. Xu, *Org. Biomol. Chem.*, 2024, **22**, 954–958.
5 CrysAlisPro, Rigaku Oxford Diffraction, 2020 & 2021.
6 O. V. Dolomanov, L. J. Bourhis, R. J. Gildea, J. A. K. Howard and H. Puschmann, *J. Appl. Crystallogr.*, 2009, **42**, 339–341.
7 G. M. Sheldrick, *Acta Crystallogr. A Found. Adv.*, 2015, **71**, 3–8.
8 G. M. Sheldrick, *Acta Crystallogr. C Struct. Chem.*, 2015, **71**, 3–8.
9 T. R. Schulte, J. J. Holstein, L. Schneider, A. Adam, G. Haberhauer and G. H. Clever, *Angew. Chem. Int. Ed.*, 2020, **59**, 22489–22493.
10 A. Zagdoun, G. Casano, O. Ouari, M. Schwarzwälder, A. J. Rossini, F. Aussenac, M. Yulikov, G. Jeschke, C. Copéret, A. Lesage, P. Tordo and L. Emsley, *J. Am. Chem. Soc.*, 2013, **135**, 12790–12797.
11 A. Kumar, B. J. Walder, A. Kunhi Mohamed, A. Hofstetter, B. Srinivasan, A. J. Rossini, K. Scrivener, L. Emsley and P. Bowen, *The Journal of Physical Chemistry C*, 2017, **121**, 17188–17196.
12 B. Gogoi, S. Khargharia, R. Kar, B. J. Borah and P. Das, *J. Mol. Struct.*, 2024, **1295**, 136678.
